# A crystalline phosphorus-substituted borinium ion: double dihydrogen activation by an ambiphilic inorganic allene

**DOI:** 10.1039/d5sc07702h

**Published:** 2025-11-18

**Authors:** Anna Ordyszewska-Lach, Kinga Cieplińska, Iwona Anusiewicz, Jarosław Chojnacki, Kinga Kaniewska-Laskowska, Rafał Grubba

**Affiliations:** a Department of Inorganic Chemistry, Faculty of Chemistry and Advanced Materials Center, Gdańsk University of Technology Narutowicza 11/12 80-233 Gdańsk Poland rafal.grubba@pg.edu.pl; b Laboratory of Quantum Chemistry, Department of Theoretical Chemistry, Faculty of Chemistry, University of Gdańsk Wita Stwosza 63 80-308 Gdańsk Poland

## Abstract

We report the synthesis of the first monomeric, isolable ambiphilic borinium ion. Bromide abstraction from a bromo(diphosphino)borane precursor affords a two-coordinate boron cation with a structure isolobal to allene. The presence of two phosphido substituents directly bonded to the boron center provides electronic stabilization, enabling the isolation of the P-substituted borinium ion in crystalline form. This compound readily forms adducts with N-heterocyclic carbenes, yielding diphosphinoborenium ions. The ambiphilic nature of the P-substituted borinium ion is demonstrated by its ability to activate dihydrogen under mild, catalyst-free conditions, highlighting its dual Lewis acidic and basic character.

## Abstract 2

The first isolable P-substituted borinium ion has been synthesized. The obtained cation exhibits ambiphilic reactivity, enabling the activation of dihydrogen under mild, catalyst-free conditions.

## Graphical abstract

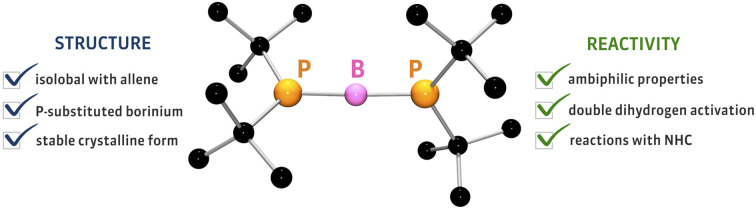

## Introduction

**Chart 1:**
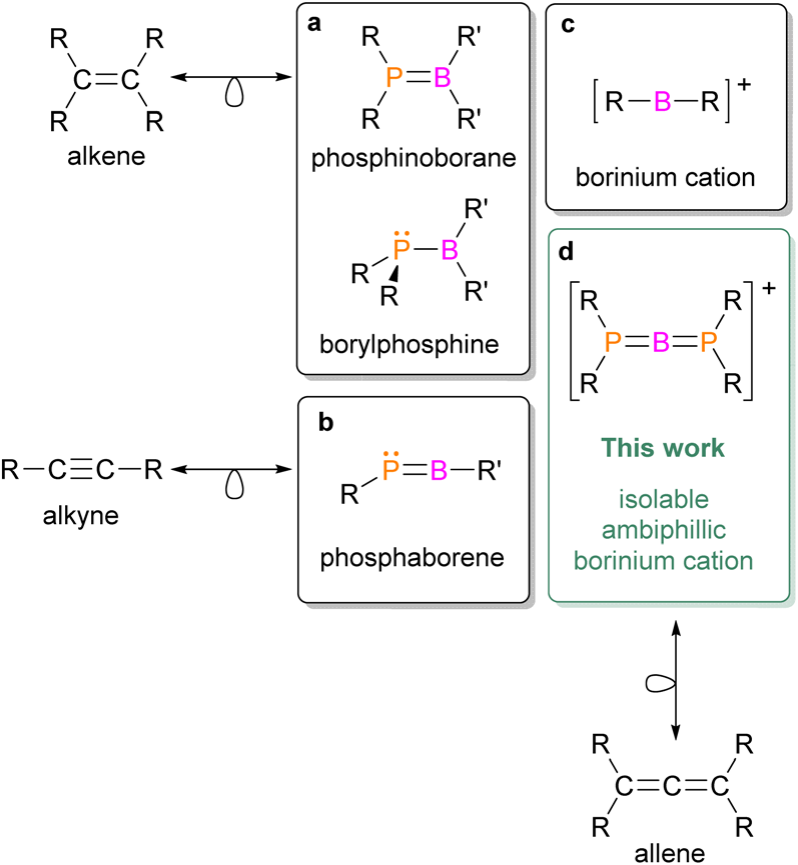
Low-coordinate boron and phosphorus species.

Ambiphilicity is a property of chemical compounds exhibiting both Lewis acid and Lewis base behaviors; that is, they can simultaneously act as electron-pair acceptors and donors. Transition metals inherently possess this attribute because they feature partially filled d orbitals of similar energy. Moreover, owing to their ambiphilic nature, transition metals are widely utilized in the activation of thermodynamically stable molecules such as dihydrogen and dinitrogen and play key roles in industrial catalytic processes. A promising alternative is the design of ambiphilic compounds derived from main-group elements.^1^ This approach allows the replacement of expensive and scarce metals with more abundant s- and p-block elements. However, it requires specialized synthetic strategies that focus on electronically and coordinatively unsaturated species. In this context, compounds containing boron and phosphorus are particularly appealing. Notably, combinations of these elements produced frustrated Lewis pairs (FLPs)—sterically hindered phosphine–borane assemblies that resist classical adduct formation and instead interact with a third (activated) molecule—thus initiating major advances in small-molecule activation and metal-free catalysis.^2–7^ Although traditional B/P-based FLPs lack a direct B–P coordination bond, recent advances in main-group chemistry have yielded low-coordinate, ambiphilic species featuring an actual B–P bond within their structure. Such species exhibit a strong tendency to dimerize; thus, the isolation of monomeric forms requires kinetic and/or electronic stabilization.^8^ Within this class of compounds, three-coordinate phosphinoboranes and borylphosphines can be distinguished, featuring a double or single B–P bond, respectively (Chart 1a).^9^ Planar and isoelectronic with alkenes, phosphinoboranes can be synthesized *via* substitution at the boron center with aromatic, electron-withdrawing groups, which have been successfully employed in dihydrogen activation.^10,11^ In contrast, the use of electron-donating ligands such as alkoxido or amido groups at the boron atom leads to the formation of borylphosphine species, characterized by elongated B–P bonds and a pyramidal P atom when compared to a planar P atom in phosphinoboranes. The broad reactivity of phosphinoboryl compounds has been demonstrated in 1,2-addition reactions to several unsaturated organic substrates.^11–18^ Moreover, these compounds can activate inorganic molecules such as CO_2_, SO_2_, and N_2_O, while remaining unreactive toward H_2_ owing to their partially quenched Lewis acidity.^19–21^ A particularly intriguing subclass of two-coordinate B–P compounds is the phosphaborenes, which are isoelectronic with alkynes (Chart 1b). Transient phosphaborenes generated *in situ* have been shown to react with phenylacetylene to yield 1,2-phosphaboretes;^22^ they can also undergo boro-phospha-Wittig-type reactions with carbonyl compounds, providing new synthetic routes to phosphaalkenes.^23^ The isolation of monomeric phosphaborenes had long remained a challenge, but was recently achieved by employing a combination of kinetic and electronic “push–pull” stabilization strategies.^24–26^ These species can also be stabilized by either Lewis acids or Lewis bases,^27,28^ confirming the ambiphilic nature of the P=B moiety in phosphaborenes.

Among the strongest known Lewis acids within boron cations chemistry are two-coordinate borinium ions,^29^ which adopt a linear geometry featuring an sp-hybridized boron center (Chart 1c). Owing to their low coordination number, these species are highly reactive and synthetically challenging. Nevertheless, viable synthetic strategies have been developed, incorporating a wide range of substituents—including amido^30,31^ and phosphinimido ligands,^32^ aryl^33,34^ and vinyl groups,^35^ and, more recently, chelating frameworks.^36^ Moreover, the pronounced Lewis acidity and oxophilicity of borinium ions have been demonstrated through their reactions with both organic and inorganic small molecules.^33,37,38^ Only one example of a reaction between a borinium ion and dihydrogen has been reported: [Mes_2_B]^+^ reacts with HD to yield a borenium–mesitylene adduct, [MesB(E)(C_6_Me_3_H_2_E)]^+^ (E = H, D). This product was identified in solution by ^11^B, ^1^H, and ^2^H{^1^H} NMR spectroscopy; however, it could not be isolated or fully characterized due to its instability.^39^

This raises a compelling question: can a Lewis basic center be incorporated into the structure of a strongly Lewis acidic borinium ion, thereby enabling the synthesis and isolation of a two-coordinate ambiphilic boron cation? Recently, we reported that *in situ* generated phosphido- and amido-substituted borinium ions, 
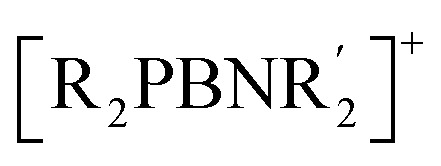
, exhibit ambiphilic properties and undergo cycloaddition reactions with ketones, isocyanates, carbodiimides, and nitriles, yielding four- and six-membered boracyclic cationic products.^40,41^ Interestingly, the amino group in these transient monomeric borinium ions was not involved in these transformations; instead, the directly bonded phosphorus and boron atoms acted as the electron pair donor and acceptor, respectively. Motivated by these findings, we pursued further investigations into the chemistry of ambiphilic boron cations and attempted to isolate these elusive intermediates.

Herein, we report the synthesis of the first isolable, ambiphilic, two-coordinate boron cation—diphosphinoborinium (Chart 1d). This species combines structural features of phosphinoboranes, phosphaborenes, and borinium ions, and is isolobal with allene. Moreover, it exhibits reactivity analogous to that of transition metals, activating one of the strongest covalent bonds—the H–H bond in dihydrogen—under remarkably mild, catalyst-free conditions.

## Results and discussion

**Scheme 1:**
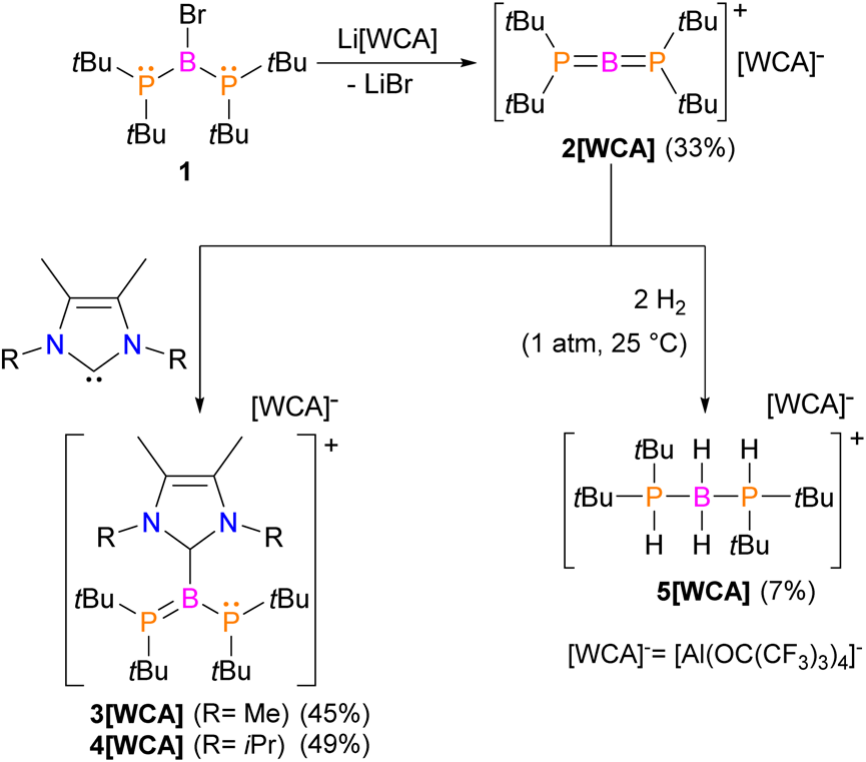
The synthesis of diphosphinoborinium ion and its reactivity toward N-heterocyclic carbenes and dihydrogen.

To obtain the P-substituted borinium ion, we reacted bromo(diphosphino)borane (1)^42^ with the lithium salt of a weakly coordinating anion (WCA^−^ = [Al(OC(CF_3_)_3_)_4_]^−^)^43^ in *o*-difluorobenzene (DFB) (Scheme 1). The progress of the reaction was monitored using ^11^B and ^31^P NMR spectroscopy. After 90 min, signals corresponding to the parent borane (1) almost disappeared, while new broad signals appeared in the ^11^B and ^31^P NMR spectra at 91.0 ppm and 146.8 ppm, respectively. These resonances are significantly downfield-shifted compared to the ^11^B and ^31^P chemical shifts of compound 1, which appear at 74.6 ppm and 46.2 ppm, respectively.^42^ The ^11^B NMR chemical shift of the newly formed compound is very close to that observed for the previously reported two-coordinate borinium ion [Mes_2_B]^+^ (93.3 ppm).^33^ Furthermore, the pronounced deshielding of the ^31^P NMR resonances can be attributed to electron pair donation from the phosphorus atoms to the two formally vacant p orbitals of the boron atom. Altogether, these spectroscopic data support the formation of a P-substituted, two-coordinate borinium ion, 2[WCA] (Scheme 1). Interestingly, in contrast to the diphosphinoborinium ion 2^+^, the reported ^11^B NMR chemical shifts for diaminoborinium derivatives are significantly upfield-shifted (33.0–38.7 ppm),^44^ a region typically associated with three-coordinate boron species. Encouraged by the NMR results, we sought to isolate the resulting borinium salt. The DFB solution of 2[WCA] was decanted from the LiBr precipitate and layered with petroleum ether. Crystallization at −22 °C yielded nearly colorless crystals of 2[WCA] in 33% yield.

**Fig. 1:**
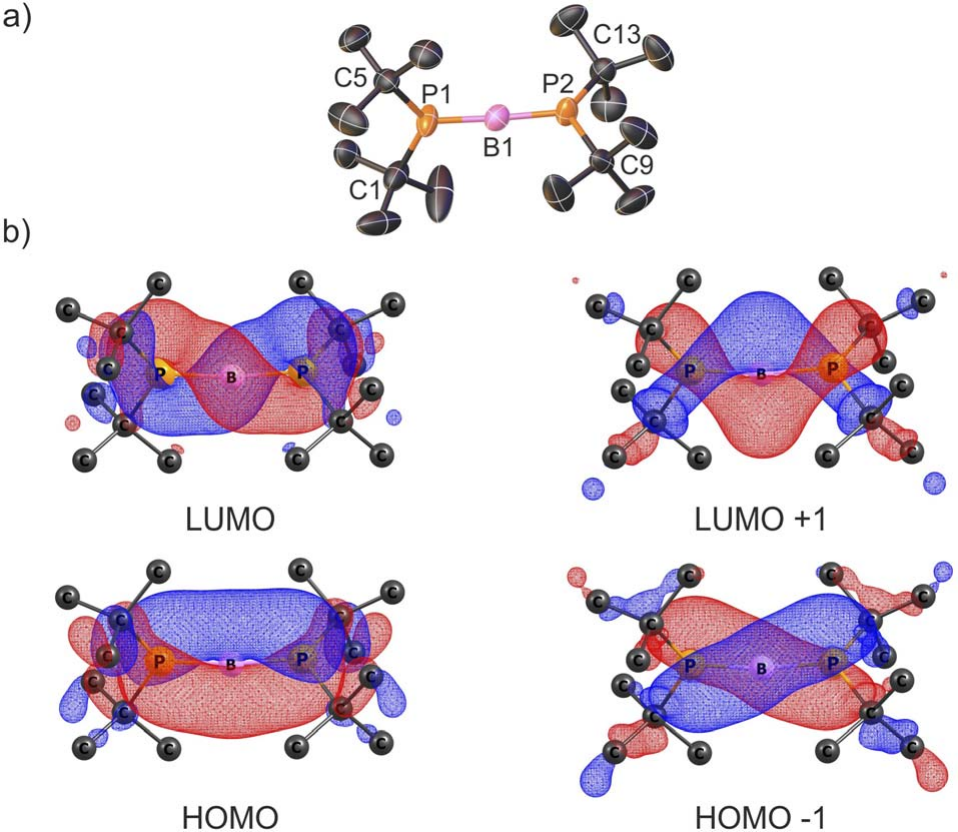
X-ray structure of cation 2^+^; counterions and H atoms are omitted for clarity; thermal ellipsoids are shown at the 50% probability level; selected bond distances [Å] and angles [°]: B1–P1 1.728(3); B1–P2 1.737(3); P1–B1–P2 176.6(2) (a). The calculated (in DFB solvent using the IEF-PCM model at ωB97xD/6-311++G** theoretical level) Kohn–Sham highest energy occupied molecular orbitals (HOMO, HOMO−1) and lowest energy unoccupied molecular orbitals (LUMO, LUMO+1) plotted with 0.03 a.u. contour value (b).

The successful isolation of crystalline 2[WCA] enabled structural elucidation *via* single-crystal X-ray diffraction (Fig. 1a). The X-ray structural analysis of cation 2^+^ confirms the conclusions drawn from NMR spectroscopy and supports the formation of a two-coordinate borinium ion, in which the central boron atom is bonded to two phosphido groups. The structure reveals an almost linear geometry around the boron center, with a P1–B1–P2 bond angle of 176.6(2)°. Furthermore, the geometries around the phosphorus atoms P1 and P2 are nearly planar, with the sums of bond angles measuring 358.6(3)° and 357.5(3)°, respectively. The planar R_2_P groups adopt an orthogonal conformation. Notably, the B1–P1 and B1–P2 bond lengths are very short and nearly identical, measuring 1.728(3) and 1.737(3) Å, respectively. To the best of our knowledge, these represent the shortest B–P bond distances reported to date. They are even slightly shorter than the B–P bond length observed in a recently isolated crystalline phosphaborene featuring two-coordinate boron and phosphorus atoms (1.741(3) Å).^25^ Remarkably, the B–P bond lengths in 2^+^ are also shorter than the sum of the double covalent bond radii of boron and phosphorus (1.80 Å),^45^ indicating multiple bond character. The most striking structural features – the very short B–P bonds and the planar, orthogonal arrangement of the phosphanyl groups – suggest the presence of π-interactions within the P–B–P fragment. Notably, the crystal structure of the borinium salt 2[WCA] demonstrates no direct interaction between 2^+^ and WCA^−^, with the shortest H2A⋯F35 contact measuring 2.567 Å. 2[WCA] crystals can be stored under an argon atmosphere at room temperature for at least several weeks. However, they are extremely sensitive to air and moisture. Additionally, while the borinium salt 2[WCA] is relatively stable in DFB, it readily reacts with other common polar solvents such as chloroform, dichloromethane, and diethyl ether.

The electronic structure of the 2^+^ cation was investigated using density functional theory (DFT) with the ωB97xD functional and the 6-311++G basis set, employing the IEF-PCM model to simulate DFB solvent. The central boron atom adopts sp hybridization, while the phosphorus atoms exhibit sp^2^ hybridization. The overlap between the two lone electron pairs on the phosphorus atoms and the formally vacant p_*x*_ and p_*y*_ orbitals of the boron atom leads to the formation of two 3-center-2-electron π-bonds. The molecular orbitals HOMO−1, HOMO, LUMO, and LUMO+1 are delocalized along the P–B–P fragment and represent these π-interactions (Fig. 1b). Notably, these molecular orbitals display a characteristic helical shape, analogous to those determined for allene and other cumulenes.^46^ The NBO analysis, together with the calculated B–P Wiberg bond order of 1.81, further supports the presence of multiple bonding. Natural population analysis reveals positive charges on the phosphorus atoms (+0.80) and a slightly negative charge on the boron atom (−0.14), suggesting significant electronic stabilization of the two-coordinate boron center by the electron-donating phosphido ligands.

In order to evaluate the Lewis acidic and Lewis basic properties, we calculated the fluoride ion affinity (FIA), hydride ion affinity (HIA), and proton affinity (PA) for 2^+^. For comparison, the same parameters were also determined for representative borinium ions, namely [(iPr_2_N)_2_B]^+^ and [Mes_2_B]^+^, at the same level of theory. Despite the strong π-donation from the phosphorus atoms to the boron center, 2^+^ exhibits a high FIA value (790.2 kJ mol^−1^), which is significantly higher than that calculated for [(iPr_2_N)_2_B]^+^ (762.0 kJ mol^−1^), and only slightly lower than that of [Mes_2_B]^+^ (801.5 kJ mol^−1^). Interestingly, compared to both the amido and mesityl derivatives, 2^+^ shows the highest HIA value (816.1 kJ mol^−1^*vs.* 771.1 kJ mol^−1^ and 804.2 kJ mol^−1^, respectively). The exceptional Lewis basicity of 2^+^ relative to known borinium ions is highlighted by the calculated PA values: it significantly outperforms both the diamido and dimesityl analogues (545.4 kJ mol^−1^*vs.* 397.8 kJ mol^−1^ and 501.3 kJ mol^−1^, respectively).

The Lewis acidity of 2^+^ was evaluated experimentally through reactions with N-heterocyclic carbenes (IMe_4_ = (MeCNMe)_2_C; IiPr_2_Me_2_ = (MeCNiPr)_2_C), as shown in Scheme 1. The reactions of 2[WCA] with NHCs in DFB afforded three-coordinate diphosphinoborenium salts 3[WCA] and 4[WCA]. Moreover, these species could be synthesized directly from precursor 1*via* sequential treatment with an NHC in petroleum ether and Li[WCA] in dichloromethane. The resulting borenium salts 3[WCA] and 4[WCA] were isolated as red crystals by crystallization from dichloromethane layered with petroleum ether or pentane, with overall yields of 45% and 49%, respectively. It is worth mentioning that during the synthesis of 2[WCA], we observed a second minor product, which most likely results from the coordination of one phosphorus atom of the parent diphosphinoborane 1 to the lithium cation; this minor product also reacts with NHCs, affording 3[WCA] or 4[WCA].

Compared to 2^+^, the cationic species 3^+^ and 4^+^ exhibit significantly upfield-shifted ^11^B and ^31^P NMR resonances. The NMR spectroscopic features of 3^+^ and 4^+^ are closely related, with ^11^B chemical shifts around 44 ppm for both cations. The ^31^P resonances differ only slightly, appearing at 78.0 ppm for 3^+^ and 81.3 ppm for 4^+^. These NMR data indicate an increase in the coordination number of boron from two to three and suggest a weakening of π-interactions between the boron and phosphorus atoms.

**Fig. 2:**
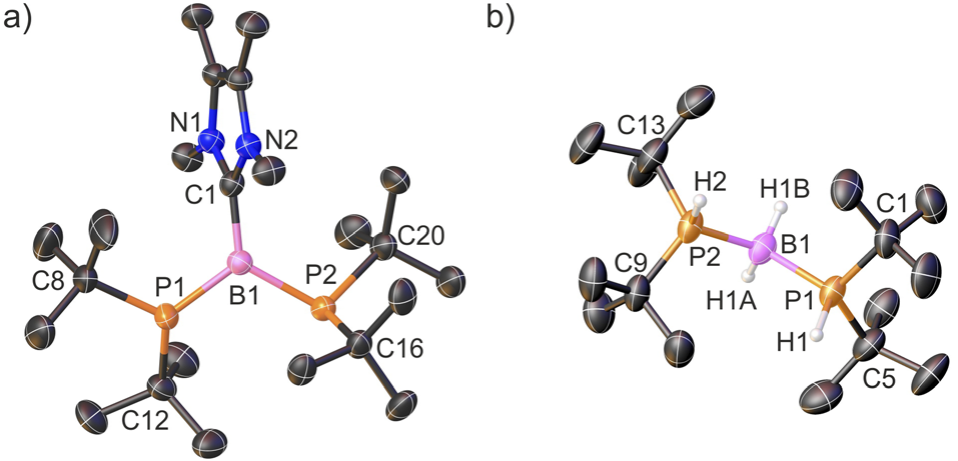
X-ray structures of 3^+^ (a) and 5^+^ (b) cations; counterions and H atoms (except those bonded with boron and phosphorus atoms) are omitted for clarity; thermal ellipsoids are shown at the 50% probability level; one of two crystallographically independent cations in the asymmetric unit is shown in each case. Selected bond distances [Å] and angles [°] for 3^+^: B1–P1 1.817(8); B1–P2 1.931(7); B1–C1 1.583(9); C1–N1 1.353(9); C1–N2 1.350(4); P1–B1–P2 118.0(4); P2–B1–C1 125.8(5); C1–B1–P1 115.5(4). Important bond distances [Å] and angles [°] for 5^+^: B1–P1 1.936(6); B1–P2 1.940(6); P1–B1–P2 118.7(3).

The X-ray structures of cations 3^+^ and 4^+^ are presented in Fig. 2a and S39, respectively. Their structural features are discussed using the representative diphosphinoborenium ion 3^+^ as a model compound. This species can be considered an adduct of the borinium ion 2^+^ and NHC ligand. The central B1 atom exhibits a trigonal planar geometry (ΣB1 = 359.3°). Interestingly, the crystal structure of 3^+^ differs from that observed in solution. In particular, the ^1^H, ^13^C, and ^31^P NMR spectra indicate that both phosphanyl groups are equivalent in solution. In contrast, in the solid state, the phosphanyl groups adopt distinct geometries: one is planar (ΣP1 = 359.4(5)°), while the other is pyramidal (ΣP2 = 326.9(6)°). Moreover, the B1–P1 bond (1.817(8) Å) is significantly shorter than the B1–P2 bond (1.931(7) Å), suggesting double and single bond character, respectively. We propose that in solution, the π-bond is delocalized across the P1–B1–P2 framework, whereas in the crystal structure, the conformer with a localized P1=B1 double bond is stabilized.

Next, we investigated the application of the diphosphinoborinium ion 2^+^ for dihydrogen activation. This molecule was selected for our experiments owing to the very strong covalent bond in H_2_ (436 kJ mol^−1^),^47^ which makes its activation particularly challenging, requiring the activator to possess both Lewis acid and base properties. A solution of 2[WCA] in DFB was frozen in liquid nitrogen, evacuated under high vacuum, and subsequently backfilled with dihydrogen gas (*p* = 1 atm). After 24 h at 25 °C, complete consumption of 2^+^ and formation of a new boronium cation, 5^+^, as the main product was confirmed by multinuclear NMR spectroscopy (Scheme 1). Despite extensive efforts, the boronium salt 5[WCA] could not be isolated in pure form and was obtained as a co-crystallized mixture with a small amount of another byproduct. However, crystals of 5[WCA] suitable for X-ray diffraction were obtained by multiple recrystallization from a diethyl ether/pentane solvent system at −22 °C with 7% yield.

The ^1^H NMR spectrum of 5^+^ displays a doublet at 1.42 ppm (^3^*J*_PH_ = 16 Hz), attributed to *tert*-butyl groups, and a doublet of triplets at 4.49 ppm (^1^*J*_PH_ = 378 Hz, ^3^*J*_HH_ = 6 Hz), assigned to P–H protons. Although signals corresponding to hydrido ligands bonded to boron are not directly observed in the ^1^H NMR spectrum, a ^1^H–^11^B HMQC experiment indicates that these signals are overlapped with the aforementioned *tert*-butyl resonance. Compared to the parent cation 2^+^, the ^11^B NMR resonance of 5^+^ is significantly upfield-shifted (−40.9 ppm), consistent with the increase in the boron coordination number from two to four. The broad ^11^B signal exhibits quintet multiplicity, which changes to a triplet upon proton decoupling. This observation suggests that the coupling constants ^1^*J*_BP_ and ^1^*J*_BH_ are of similar magnitude (∼90 Hz). The ^31^P{^1^H} NMR spectrum of 5^+^ exhibits a broad multiplet at 36.2 ppm (^1^*J*_BP_ = 90 Hz), which further splits upon the removal of proton decoupling (^1^*J*_PH_ = 378 Hz). The large ^1^*J*_PH_ coupling constant indicates the presence of a four-coordinate phosphorus atom.

The NMR data are consistent with the single-crystal X-ray structure of 5[WCA] (Fig. 2b). The boronium cation 5^+^ can be formally considered as an adduct of the simplest borinium ion [BH_2_]^+^ with two *t*Bu_2_PH ligands. Notably, the hydrogen atoms directly bonded to phosphorus and boron were located based on the Fourier electron density map (Fig. S40). As expected, compared to 2^+^, the P1–B1–P2 bond angle is significantly more acute, decreasing from 176.6(2)° to 118.7(3)°. Concurrently, the B1–P1 and B1–P2 bond lengths are noticeably elongated from 1.728(3) Å and 1.737(3) Å to 1.936(6) Å and 1.940(6) Å, respectively. Although a smaller P1–B1–P2 angle is generally expected for a four-coordinate boron center, the observed value can be rationalized by steric repulsion between the bulky phosphine ligands. Furthermore, the computed geometric parameters for 5^+^ (B1–P1 = 1.940 Å; B1–P2 = 1.942 Å; P1–B1–P2 = 120.94°) are in excellent agreement with the experimental X-ray crystal structure.

Collectively, the NMR and X-ray data confirm the heterolytic cleavage of two H_2_ molecules by the borinium ion 2^+^, resulting in the formation of a new four-coordinate boronium cation 5^+^, which represents the first example of an isolable adduct of borinium ion and hydrogen molecule. Additionally, the reactions of borenium derivatives 3^+^ and 4^+^ with H_2_ were investigated, but no reaction occurred under the same conditions as those used for 2^+^.

**Fig. 3:**
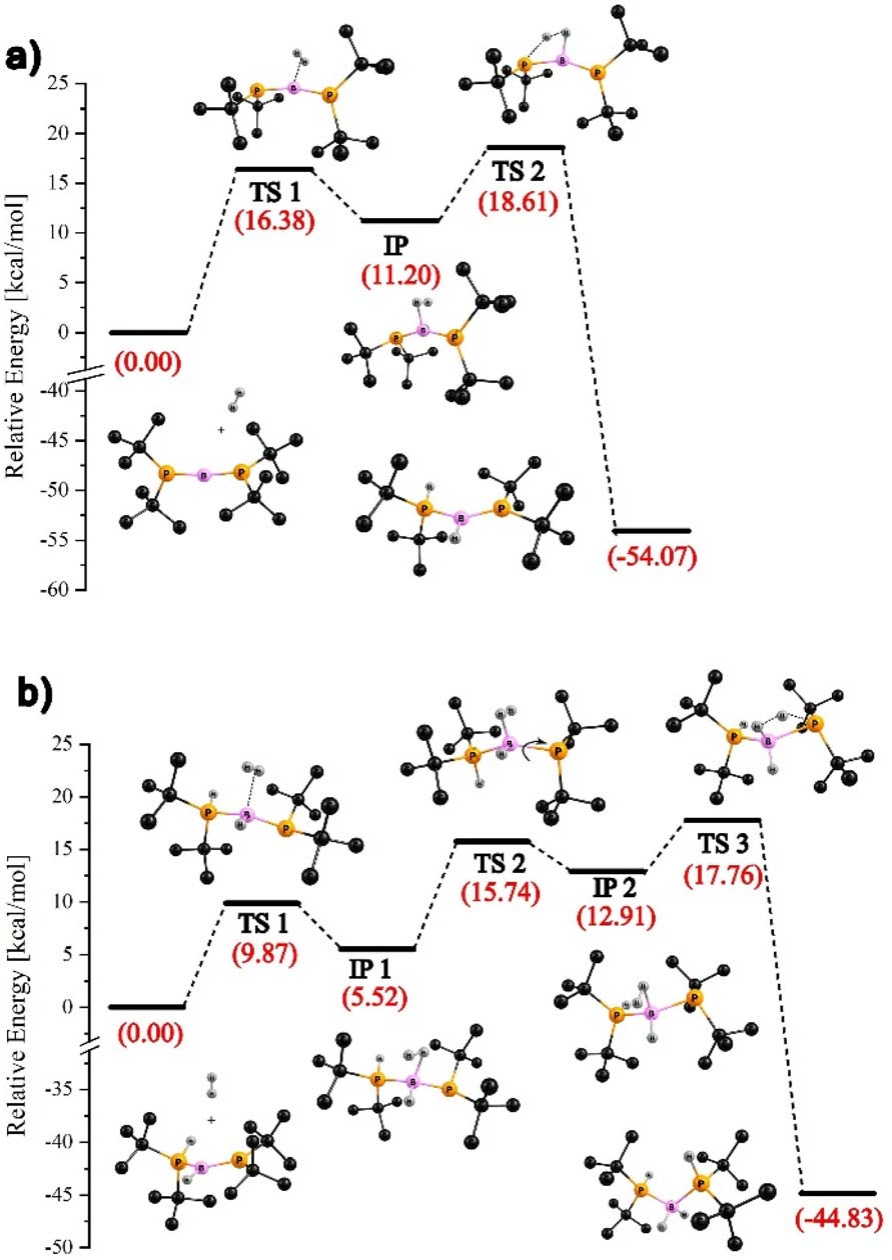
ωB97xD/6-311++G** energy profile (in DFB as solvent using the IEF-PCM model) for the reaction of 2^+^ with dihydrogen: activation of the first (a) and second H_2_ molecule (b).

According to our theoretical predictions, the activation of the first H_2_ molecule by [*t*Bu_2_P1=B1=P2*t*Bu_2_]^+^ (2^+^) proceeds *via* two main steps. As illustrated in Fig. 3a, the H_2_ molecule initially coordinates to the boron center through the transition state TS1. This is followed by interaction with the P1 atom, leading to H–H bond cleavage *via*TS2 and the formation of the intermediate borenium ion [*t*Bu_2_P1(H)–B1(H)=P2*t*Bu_2_]^+^. These two steps involve kinetic barriers of 16.4 and 18.6 kcal mol^−1^, respectively (see Fig. 3a). The activation of the second H_2_ molecule follows a similar two-step pathway: coordination to boron, followed by its heterolytic cleavage, resulting in the formation of [*t*Bu_2_P1(H)–B1(H_2_)–P2(H)*t*Bu_2_]^+^ (5^+^) (Fig. 3b).

This process proceeds with slightly lower energy barriers of 9.9 and 17.8 kcal mol^−1^, respectively (TS1 and TS3). Additionally, an extra step appears in the reaction energy profile, involving rotation around the B1–P2 bond (TS2). This step is associated with the formation of an intermediate complex (labeled IP1) upon binding of the second H_2_ molecule. The interaction induces a shift of an electron pair from the B1=P2 double bond toward the P2 atom, enhancing its Lewis basic character and facilitating its interaction with the second H_2_ molecule. Notably, in both activation sequences, the rate-determining step is the dissociation of the H_2_ molecule following initial adduct formation. The computed reaction energy profiles shown in Fig. 3 indicate that the activation of both the first and second dihydrogen molecules by 2^+^ is highly exergonic, with a total free energy release of approximately 100 kcal mol^−1^.

Interestingly, the mechanism of H_2_ activation by 2^+^ resembles that calculated for the reaction of H_2_ with the intramolecular FLPs – in particular with Mes_2_PCH_2_CH(Me)CH_2_B(C_6_F_5_)_2_.^48,49^ Similarly, as shown by our calculations for the reaction involving 2^+^, in the case of H_2_ activation by the intramolecular FLP, the hydrogen molecule first coordinates side-on to the boron center, followed by interaction with the phosphorus atom, which abstracts a proton from the activated H_2_ molecule. Moreover, the structures of TS2 and TS3, presented in Fig. 3a and b, respectively, are analogous to the structure of the transition state calculated for the aforementioned intramolecular FLP reaction, featuring an H⋯H⋯P fragment that additionally interacts with the boron center *via* hydrogen atoms.^49^

## Conclusions

In conclusion, our results demonstrate that an ambiphilic borinium ion can be successfully synthesized and isolated. The incorporation of electron-donating phosphido groups at the boron center provides substantial electronic stabilization and results in an allenic structure with two delocalized π-bonds across the P–B–P fragment. Despite the electron donation from the phosphorus atoms to the central boron atom, 2^+^ retains pronounced Lewis acidic as well as basic character, as supported by FIA, HIA, and PA calculations. Its reactions with NHCs experimentally confirm the Lewis acidity of 2^+^ and provide access to a new class of borenium ions featuring two B–P bonds. Remarkably, the dual Lewis acidic and basic nature of 2^+^ is demonstrated by the activation of dihydrogen under mild, catalyst-free conditions, in which the boron and phosphorus atoms cooperatively interact with the H_2_ molecule, thereby mimicking intramolecular FLP-type reactivity. In contrast to the analogous reaction of [Mes_2_B]^+^ yielding the unstable product, 2^+^ is capable of activating up to two H_2_ molecules, affording an isolable boronium derivative that was characterized in both the solid state and in solution. These findings pave the way for the development of novel boron-based cationic species for small-molecule activation.

## Author contributions

A. O.-L.: investigation, conceptualization, visualization, writing – original draft; K. C.: investigation, I. A.: formal analysis, writing – original draft; J. C.: investigation, formal analysis; K. K.-L.: funding acquisition, visualization, writing – original draft; R. G.: supervision, conceptualization, writing – original draft.

## Conflicts of interest

There are no conflicts to declare.

## Supplementary Material

SC-017-D5SC07702H-s001

SC-017-D5SC07702H-s002

## Data Availability

CCDC 2455327 (2[WCA]), 2455328 (3[WCA]), 2455329 (4[WCA]) and 2455330 (5[WCA]) contain the supplementary crystallographic data for this paper.^50*a*–*d*^ The data supporting this article have been included as part of the supplementary information (SI). Supplementary information: experimental, crystallographic, spectroscopic and computational details. See DOI: https://doi.org/10.1039/d5sc07702h.
