# Supplementary material for: A crystalline phosphorus-substituted borinium ion: double dihydrogen activation by an ambiphilic inorganic allene
Source: Chem Sci. 2025 Nov 18;17(2):1288–93. doi: 10.1039/d5sc07702h (PMC12643039; doi:10.1039/d5sc07702h)
Supplement: SC-017-D5SC07702H-s001 [file SC-017-D5SC07702H-s001.pdf]

Supplementary Information (SI) for Chemical Science.
This journal is © The Royal Society of Chemistry 2025

# Supplementary Information

## A Crystalline Phosphorus-Substituted Borinium Ion: Double Dihydrogen Activation by an Ambiphilic Inorganic Allene

Anna Ordyszewska-Lach[a]*, Kinga Cieplińska[a], Iwona Anusiewicz[b], Jarosław Chojnacki[a], Kinga Kaniewska-Laskowska[a], Rafał Grubba[a]*

*corresponding author, E-mail: rafal.grubba@pg.edu.pl, anna.ordyszewska-lach@pg.edu.pl

[a]Department of Inorganic Chemistry, Faculty of Chemistry and Advanced Materials Center, Gdańsk University of Technology, Narutowicza 11/12, 80-233 Gdańsk, Poland;

[b]Laboratory of Quantum Chemistry, Department of Theoretical Chemistry, Faculty of Chemistry, University of Gdańsk, Wita Stwosza 63, 80-308 Gdańsk, Poland.

# Table of Contents


Experimental section ........ 3

Materials and methods ........ 3

Synthesis of 2[WCA] ........ 3

Synthesis of 3[WCA] ........ 4

Synthesis of 4[WCA] ........ 5

Synthesis of 5[WCA] ........ 6

NMR spectra ........ 7

NMR spectra of 2[WCA] ........ 8

NMR spectra of reaction mixtures - reaction of 2[WCA] with $IMe_4$ ........ 13

NMR spectra of reaction mixtures - reaction of generated in situ 2[WCA] with $^{Me}$I*i*$Pr_2$ ........ 14

NMR spectra of 3[WCA] ........ 15

NMR spectra of 4[WCA] ........ 19

NMR spectra of 5[WCA] reaction mixture ........ 23

NMR spectra of 5[WCA] ........ 24

X-ray structure analysis ........ 30

X-ray structure analysis details ........ 30

X-ray structure analysis of 4[WCA] ........ 33

X-ray differential Fourier electron density map of 5[WCA] ........ 33

DFT calculations ........ 34

References ........ 51

# Experimental section

## Materials and methods

All experimental manipulations were performed under an inert dry argon atmosphere using flame-dried Schlenk-type glassware, either on a high-vacuum line or in an argon-filled glovebox.[1] All solvents were purchased from POCH except for DFB, which was obtained from Apollo Scientific. Solvents were purified and dried using standard procedures: petroleum ether with a K/Na alloy, toluene with Na/benzofenone, DFB with $CaH_2$ and $CH_2Cl_2$ with phosphorus pentoxide. Solvents for NMR spectroscopy ($C_6D_6$, $CD_2Cl_2$) were purchased from Deutero and purified with metallic sodium ($C_6D_6$) or phosphorus pentoxide ($CD_2Cl_2$). 1D ($^{1}H$, $^{11}B$, $^{31}P$, $^{13}C$, $^{27}Al$, $^{19}F$) and 2D NMR spectra were collected on a Bruker AV400 MHz spectrometer (external standard TMS for $^{1}H$ and $^{13}C$; $BF_3{\cdot}Et_2O$ for $^{11}B$, 85% $H_3PO_4$ for $^{31}P$; $[Al(H_2O)_6]^{3+}$ for $^{27}Al$, $CFCl_3$ for $^{19}F$) at 25°C. $^{1}H$ and $^{13}C\{^{1}H\}$, chemical shifts are reported in ppm relative to residual solvent peaks at 5.32 and 53.84 ppm for $CD_2Cl_2$ and 7.16 and 128.06 ppm for $C_6D_6$, respectively.[2] Reaction progress was monitored by $^{11}B$, $^{31}P\{^{1}H\}$, and $^{31}P$ NMR spectroscopy of the reaction mixtures. All NMR data were processed using the Bruker Topspin 3.5 software. CHN elemental analyses of all compounds were performed using an Elementar Vario MACRO analyzer (Elementar Analysesysteme GmbH) in Instrumental Analysis Laboratory, Faculty of Chemistry, Nicolaus Copernicus University in Toruń, Poland. $(tBu_2P)_2BBr$[3], $IMe_4$[4], $^{Me}IiPr_2$[4], and $Li[Al(OC(CF_3)_3)_4]$[5] were prepared according to literature procedures.

## Synthesis of 2[WCA]

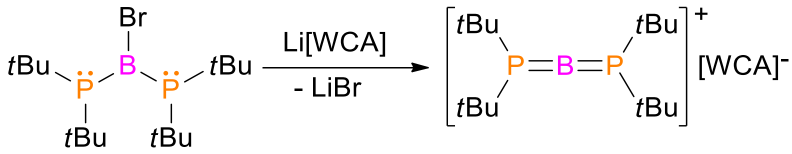

1 2[WCA]

**Scheme S1.** Synthesis of borinium cation **2**$^{+}$

A dark red solution of bromo(diphosphino)borane **1** (0.152 g, 0.400 mmol) in DFB (2.5 mL) was added dropwise to a stirred suspension of white $Li[Al(OC(CF_3)_3)_4]$ (0.390 g, 0.400 mmol) in DFB (1 mL) at -20 °C. The mixture was allowed to warm to room temperature and stirred for 1.5 h. The LiBr precipitate was then removed, and the obtained clear dark purple solution was layered with 2 mL of petroleum ether, giving single colorless crystals of **2[WCA]** (0.165 g, 0.130 mmol, yield: 32.5%) suitable for X-ray diffraction at -22°C. Elemental analysis calc. for $C_{32}H_{36}AlBF_{36}O_4P_2$ (1268.31 g/mol): C, 30.30; H, 2.861. Found: C, 30.29; H, 2.934. Due to the extremely high reactivity of **2[WCA]**, we were unable to dissolve

the product in accessible NMR polar solvents; therefore, NMR spectra were detected for a concentrated solution of **2[WCA]** in DFB/$C_6D_6$. $^{11}$B NMR ($C_6D_6$): δ 91.0 (bm). $^{31}$P NMR ($C_6D_6$): δ 146.8 (bm). $^{1}$H NMR ($C_6D_6$): δ 1.27 (bm, overlapped, 36 H, C(C**H**$_3$)$_3$). $^{13}$C{$^{1}$H} NMR ($C_6D_6$): δ 122.3 (q, $^{1}J_{CF}$ = 292.5 Hz, OC(**C**F$_3$)$_3$); 41.5 (m, overlapped, **C**(CH$_3$)$_3$); 31.6 (s, C(**C**H$_3$)$_3$). $^{27}$Al NMR ($C_6D_6$): δ 35.1 (s, [**Al**(OC(CF$_3$)$_3$)$_4$]$^-$). $^{19}$F NMR ($CD_2Cl_2$): δ -75.2 (s, OC(C**F**$_3$)$_3$).

# Synthesis of 3[WCA]

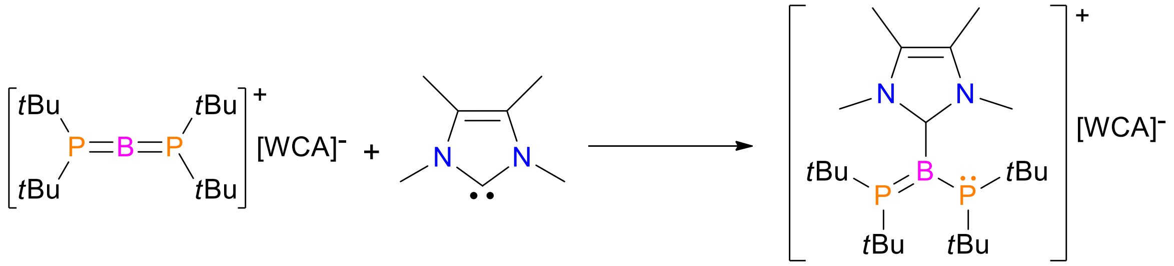


**2[WCA]** **3[WCA]**

**Scheme S2.** Method A: synthesis of NHC-stabilized borenium cation **3⁺**

**Method A:** To a dark purple solution of **2[WCA]** (0.137 g, 0.108 mmol) in DFB (2.0 mL) at -30 °C, a solution of 1,3,4,5-tetramethylimidazol-2-ylidene (IMe$_4$) (0.014 g, 0.113 mmol, 5% excess) in a minimal volume of DFB was added dropwise. The reaction mixture became red almost immediately and was allowed to warm to ambient temperature and stirred for 30 min. The formation of **3[WCA]** was confirmed by $^{31}$P and $^{11}$B NMR spectroscopy (Figure S9).

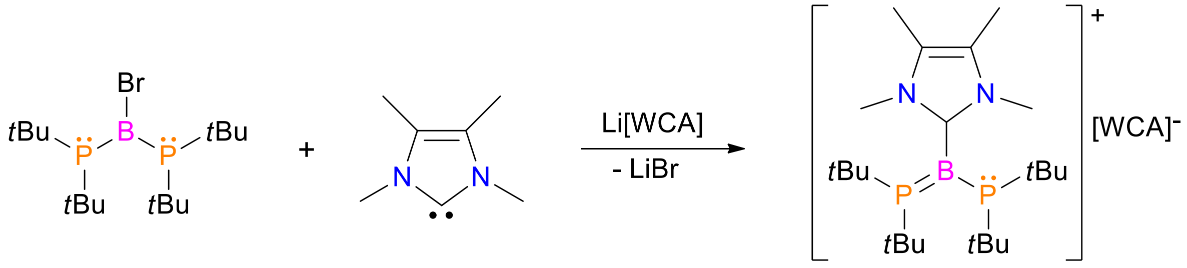


**1** **3[WCA]**

**Scheme S3**. Method B: synthesis of NHC-stabilized borenium cation **3⁺**

**Method B:** A solution of bromo(diphosphino)borane **1** (0.234 g, 0.614 mmol) in petroleum ether (4 mL) was added dropwise to a stirred suspension of *N*-heterocyclic carbene (1,3,4,5-tetramethylimidazol-2-ylidene, IMe$_4$, 0.076 g, 0.614 mmol) in petroleum ether (2 mL) at -30 °C. The mixture was allowed to warm to room temperature and stirred for 1 hour. After filtration of the suspension, evaporation of all volatiles under reduced pressure afforded a red crude product of borenium salt (0.233 g, 0.461 mmol, yield: 75.1%).

Afterwards, a solution of resulting borinium salt (0.227 g, 0.450 mmol) in $CH_2Cl_2$ (2 mL) was added dropwise to a stirred suspension of Li[Al(OC(CF$_3$)$_3$)$_4$] (0.487 g, 0.500 mmol) in $CH_2Cl_2$ (3 mL) at -30 °C. The mixture was allowed to warm to room temperature and stirred overnight. The LiBr precipitate was

then removed and the obtained clear dark red solution was concentrated to 1 mL. Single crystals of **3[WCA]** (0.377 g, 0.271 mmol, yield: 60.2%) suitable for X-ray diffraction were obtained from $CH_2Cl_2$ solution (2 mL) layered with petroleum ether (2 mL) stored at -22 °C. Overall yield of the reaction: 45.2%. Elemental analysis calc. for $C_{39}H_{48}AlBF_{36}N_2O_4P_2$ (1392.49 g/mol): C, 31.88; H, 3.568; N, 2.06. Found: C, 31.90; H, 3.642; N, 2.141. $^{11}$B NMR ($CD_2Cl_2$): δ 44.4 (bs). $^{31}$P NMR ($CD_2Cl_2$): δ 78.0 (bs). $^{1}$H NMR ($CD_2Cl_2$): δ 3.72 (s, 6 H, NC**H**$_3$ of IMe$_4$); 2.27 (s, 6 H, =CC**H**$_3$ of IMe$_4$); 1.37 (m, overlapped, 36 H, C(C**H**$_3$)$_3$). $^{13}$C{$^{1}$H} NMR ($CD_2Cl_2$): δ 129.3 (s, H$_3$C**C**=**C**CH$_3$ of IMe$_4$); 121.7 (q, $^{1}J_{CF}$ = 292.8 Hz, OC(**C**F$_3$)$_3$); 38.6 (bs, overlapped, **C**(CH$_3$)$_3$); 35.7 (s, N**C**H$_3$ of IMe$_4$); 33.6 (m, overlapped, C(**C**H$_3$)$_3$); 9.1 (s, H$_3$**C**C=C**C**H$_3$ of IMe$_4$). The carbene carbon atom was not detected in the $^{13}$C{$^{1}$H} spectrum; however, the chemical shift of the carbene carbon atom was identified at 151.8 ppm based on the $^{13}$C $^{1}$H HMBC spectrum. $^{27}$Al NMR ($CD_2Cl_2$): δ 34.6 (s, [**Al**(OC(CF$_3$)$_3$)$_4$]$^-$). $^{19}$F NMR ($CD_2Cl_2$): δ -75.7 (s, OC(C**F**$_3$)$_3$).

# Synthesis of 4[WCA]

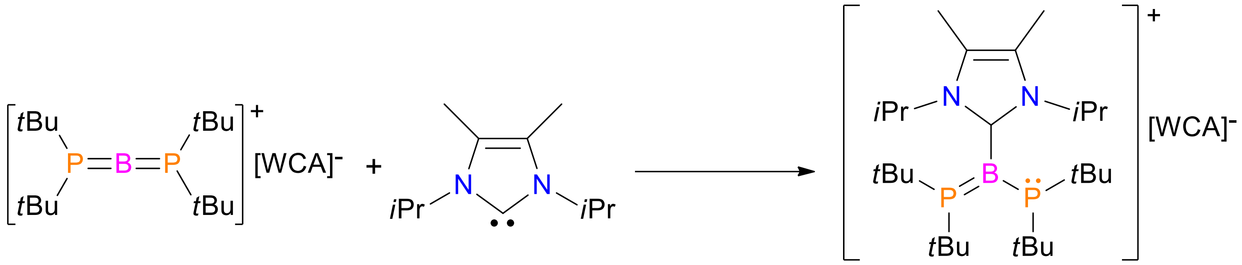


**2[WCA]** **4[WCA]**

**Scheme S4.** Method A: synthesis of NHC-stabilized borenium cation **4⁺**

**Method A:** A clear solution of **2[WCA]** (0.380 g; 0.300 mmol) in 2 mL of DFB was treated with a solution of the N-heterocyclic carbene (1,3-diisopropyl-4,5-dimethylimidazol-2-ylidene, $^{Me}$I*i*Pr$_2$, 0.566 g, 0.315 mmol) in 2 mL of DFB, and the mixture was stirred at room temperature for 1 hour. The color change was observed from dark brownish-purple to brownish-orange. The formation of **4[WCA]** was confirmed by $^{31}$P and $^{11}$B NMR spectroscopy (Figure S10).

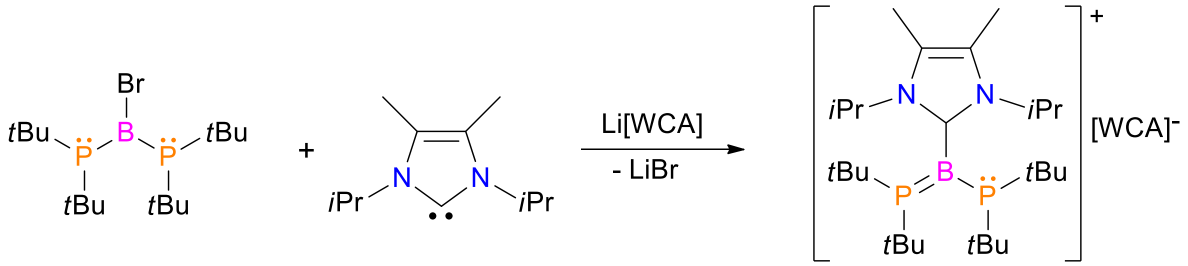


**1** **4[WCA]**

**Scheme S5.** Method B: synthesis of NHC-stabilized borenium cation **4⁺**

**Method B:** A red solution of bromo(diphosphino)borane **1** (0.280 g, 0.735 mmol) in petroleum ether (4 mL) was added dropwise to a stirred suspension of *N*-heterocyclic carbene (1,3-diisopropyl-4,5-dimethylimidazol-2-ylidene, $^{Me}$I*i*Pr$_2$, 0.132 g, 0.735 mmol) in petroleum ether (2 mL) at -30 °C. The

mixture was allowed to warm to room temperature and stirred for 1 hour. After filtration of the suspension, evaporation of all volatiles under reduced pressure afforded a red crude product of borenium salt (0.27 g, 0.480 mmol, yield: 65.3%).

Afterwards, a red solution of resulting borinium salt (0.085 g, 0.150 mmol) in $CH_2Cl_2$ (2 mL) was added dropwise to a stirred suspension of $Li[Al(OC(CF_3)_3)_4]$ (0.146 g, 0.150 mmol) in $CH_2Cl_2$ (3 mL) at -30 °C. The mixture was allowed to warm to room temperature and stirred overnight. The LiBr precipitate was then removed and the obtained clear red solution was concentrated to 1 mL. Single red crystals of **4[WCA]** (0.162 g, 0.112 mmol, yield: 74.5%) suitable for X-ray diffraction were obtained from $CH_2Cl_2$ solution (1 mL) layered with pentane (2 mL) stored at +4 °C. Overall yield of the reaction: 48.7%. Elemental analysis calc. for $C_{43}H_{56}AlBF_{36}N_2O_4P_2$ (1448.60 g/mol): C, 35.65; H, 3.896; N, 1.934. Found: C, 35.62; H, 3.860; N, 1.897. $^{11}B$ NMR ($CD_2Cl_2$): δ 44.5 (bs). $^{31}P$ NMR ($CD_2Cl_2$): δ 81.3 (bs). $^{1}H$ NMR ($CD_2Cl_2$): δ 5.05 (sept, 2 H, $^{3}J_{HH}$ = 7.0 Hz, C**H**($CH_3$)$_2$ of $^{Me}$I*i*Pr); 2.38 (s, 6 H, C**H**$_3$ of $^{Me}$I*i*Pr); 1.60 (d, 12 H, $^{3}J_{HH}$ = 7.0 Hz, CH(C**H**$_3$)$_2$ of $^{Me}$I*i*Pr); 1.43 (m, overlapped, 36 H, C(C**H**$_3$)$_3$). $^{13}C\{^{1}H\}$ NMR ($CD_2Cl_2$): δ 130.3 (s, $H_3$C**C**=**C**C$H_3$ of $^{Me}$I*i*Pr); 121.7 (q, $^{1}J_{CF}$ = 292.6 Hz, OC(**C**$F_3$)$_3$); 52.9 (m, overlapped, **C**H($CH_3$)$_2$ of $^{Me}$I*i*Pr); 39.7 (m, overlapped, **C**($CH_3$)$_3$); 33.9 (m, overlapped, C(**C**$H_3$)$_3$); 22.1 (s, CH(**C**$H_3$)$_2$ of $^{Me}$I*i*Pr); 11.2 (s, $H_3$**C**C=C**C**$H_3$ of $^{Me}$I*i*Pr). The carbene carbon atom was not detected. $^{27}Al$ NMR ($CD_2Cl_2$): δ 34.6 (s, [**Al**($OC(CF_3)_3)_4]^-$). $^{19}F$ NMR ($CD_2Cl_2$): δ -75.7 (s, OC(C**F**$_3$)$_3$).

# Synthesis of 5[WCA]

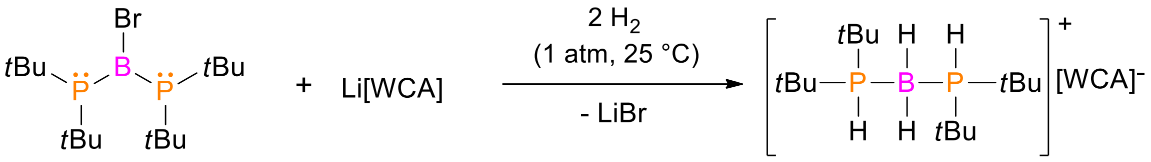


**1** **5[WCA]**

**Scheme S6.** Synthesis of boronium cation **5**$^+$

A solution of bromo(diphosphino)borane **1** (0.152 g, 0.400 mmol) in DFB (2.5 mL) was added dropwise to a stirred suspension of $Li[Al(OC(CF_3)_3)_4]$ (0.390 g, 0.400 mmol) in DFB (1 mL) at -20 °C and stirred for about 1 h at RT. The LiBr precipitate was then removed, and the resulting dark purple solution was frozen in liquid nitrogen. Hydrogen gas ($H_2$) was introduced by displacing argon and applying $H_2$ at 1 atm. The reaction mixture was then allowed to warm to room temperature applying pressure stabilizing system (at level about 1.1 atm) containing mercury bubbler. After reaching RT, the pressure stabilizing system was cut off and the resulting solution was stirred for 24 h. No visible color change was observed. The reaction yield, estimated based on the $^{31}P$ NMR spectrum of the reaction mixture, was 70%. X-ray quality single colorless crystals of **5[WCA]** were obtained by solvent evaporation followed by multiple recrystallizations from diethyl ether layered with pentane at –22 °C 0.034 g, 0.027 mmol, yield: 6.8%). Elemental analysis could not be performed due to the strong tendency of the

product to undergo hydrolysis. $^{11}$B NMR ($CD_2Cl_2$): δ -40.9 (bm). $^{31}$P NMR ($CD_2Cl_2$): δ 36.1 (bd, $^1J_{PH}$ = 378.0 Hz). $^1$H NMR ($CD_2Cl_2$): δ 4.49 (dt, $^1J_{PH}$ = 378.0 Hz, $^3J_{HH}$ = 6.3 Hz, 2H, P**H**), 1.42 (d, $^3J_{P\text{-}H}$ = 15.7 Hz, overlapped, 38H, B**H**$_2$ and C(C**H**$_3$)$_3$)). $^{13}$C{$^1$H} NMR ($CD_2Cl_2$): δ 121.7 (q, $^1J_{CF}$ = 292.6 Hz, OC(**C**F$_3$)$_3$), 33.7 (m, overlapped, **C**(CH$_3$)$_3$), 28.9 (s C(**C**H$_3$)$_3$). $^{27}$Al NMR ($CD_2Cl_2$): δ 34.6 (s, [**Al**(OC(CF$_3$)$_3$)$_4$]$^-$). $^{19}$F NMR ($CD_2Cl_2$): δ -75.7 (s, OC(C**F**$_3$)$_3$).

# NMR spectra

Abbreviations

| | |
|---|---|
| s | deuterated solvent (residual signal) |
| g | grease |
| a | adduct **1** with Li[WCA] |
| ★ | impurity |
| ● | 1,2-difluorobenzene |
| ● | *t*Bu$_2$PH |
| ● | imidazolium salt |
| ● | *t*Bu$_2$PP*t*Bu$_2$ |
| ● | Et$_2$O |

## NMR spectra of 2[WCA]

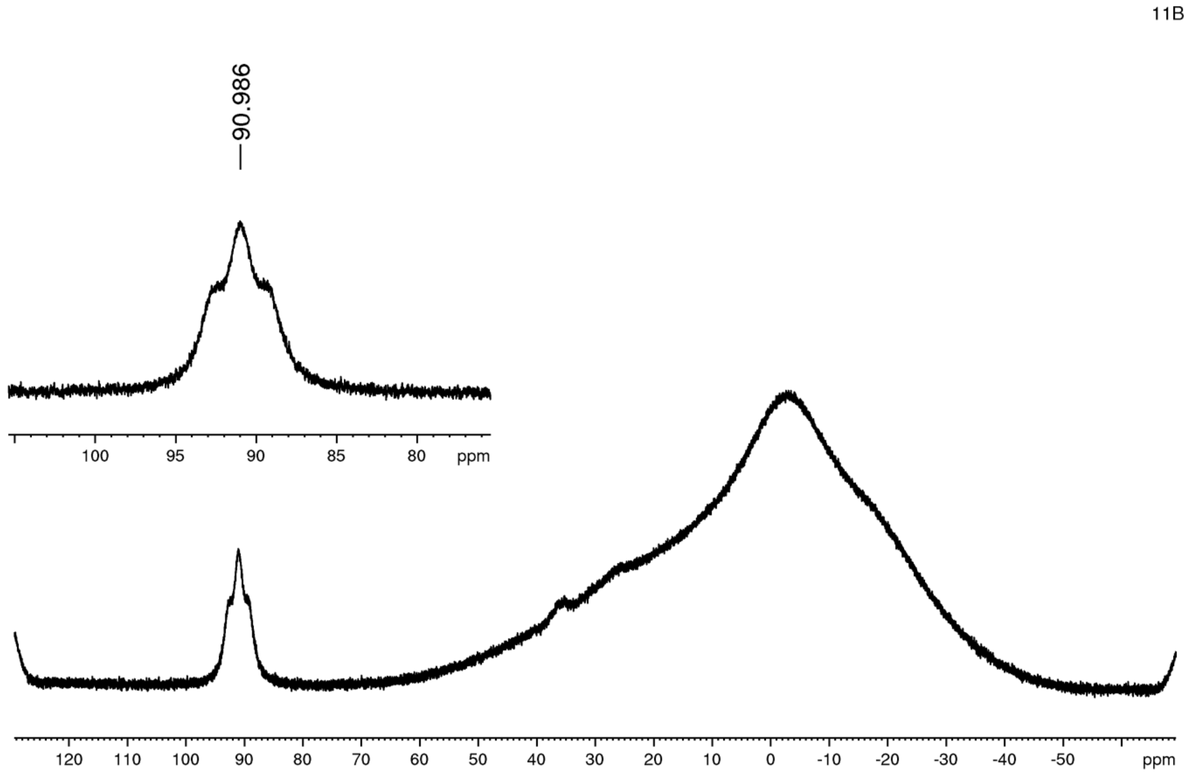


**Figure S1.** $^{11}B$ spectrum ($C_6D_6$) of **2[WCA]**

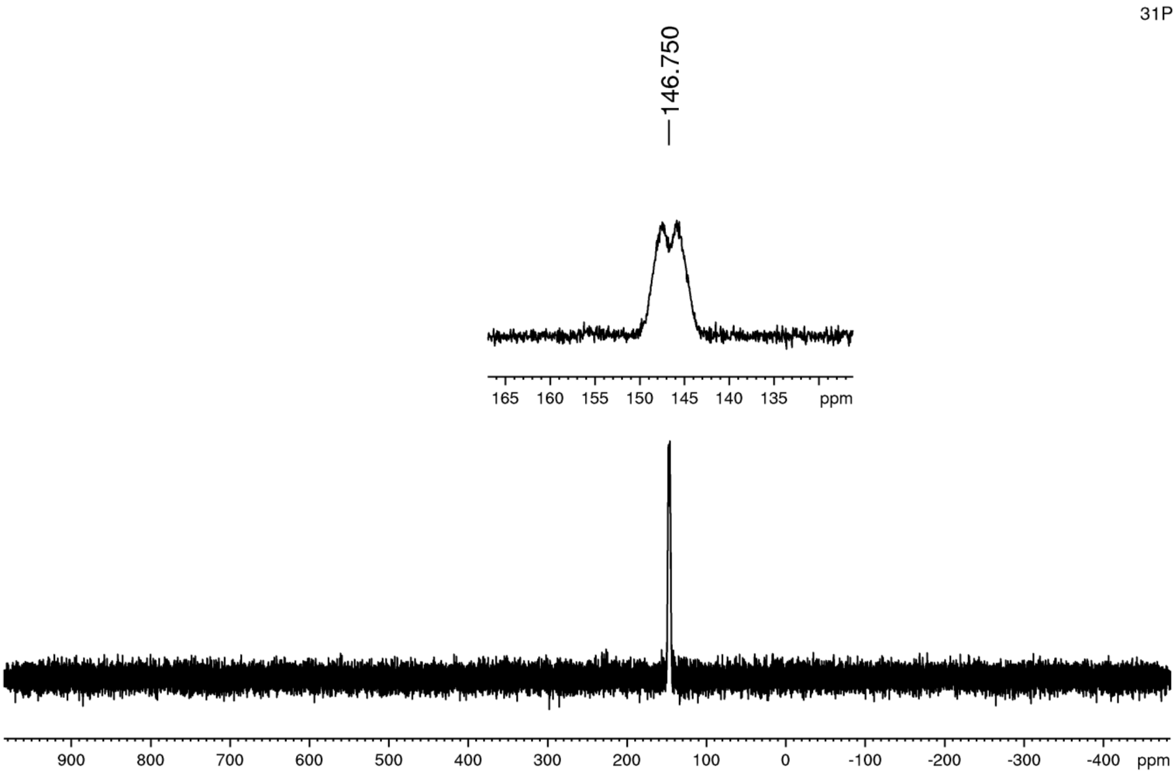


**Figure S2.** $^{31}P$ spectrum ($C_6D_6$) of **2[WCA]**

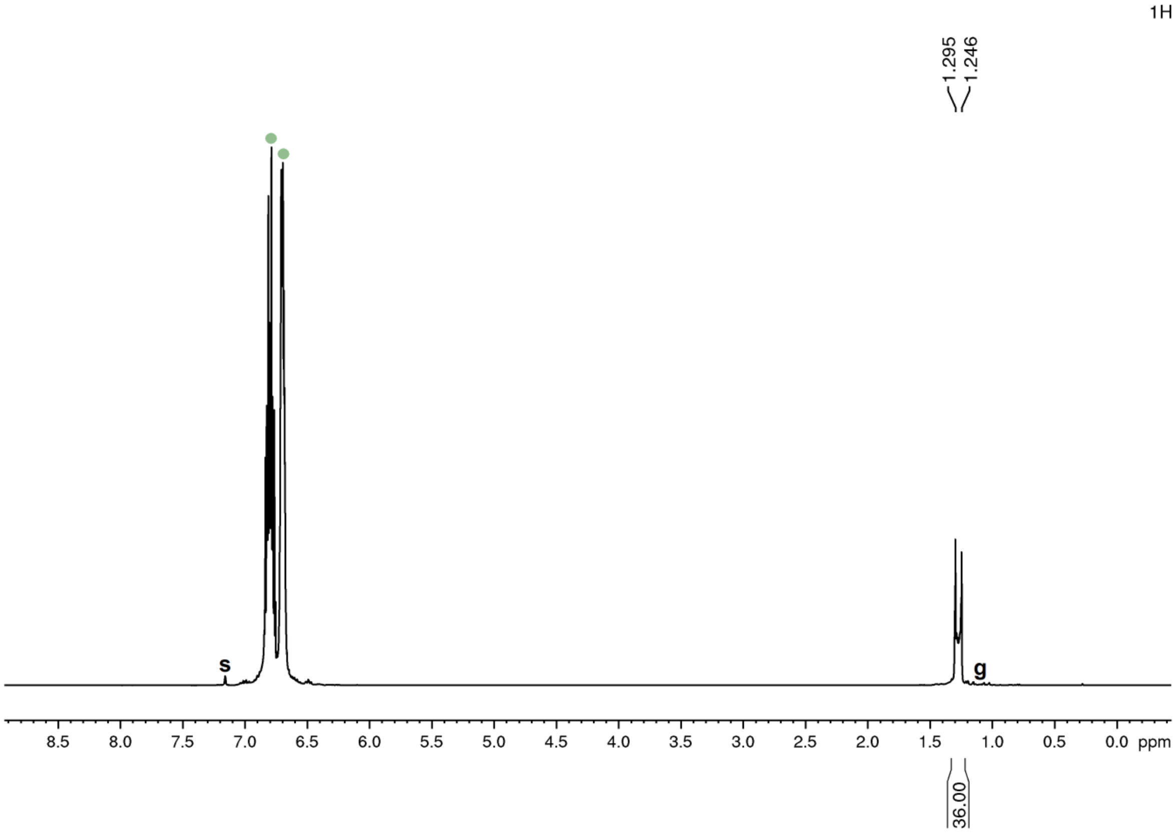


**Figure S3.** $^{1}H$ spectrum ($C_6D_6$) of **2[WCA]**
(**s** – deuterated solvent (residual signal); **g** – grease; ● – 1,2-difluorobenzene)

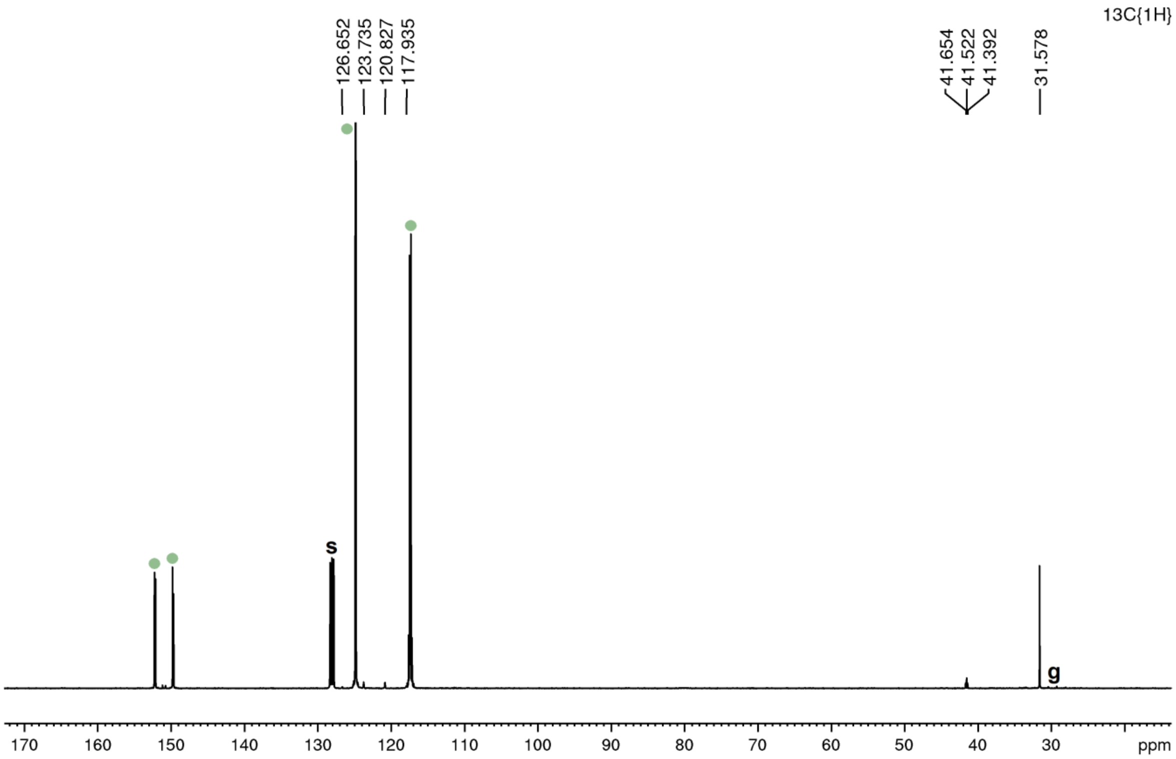


**Figure S4.** $^{31}C\{^{1}H\}$ spectrum ($C_6D_6$) of **2[WCA]**
(**s** – deuterated solvent (residual signal); **g** – grease; ● – 1,2-difluorobenzene)

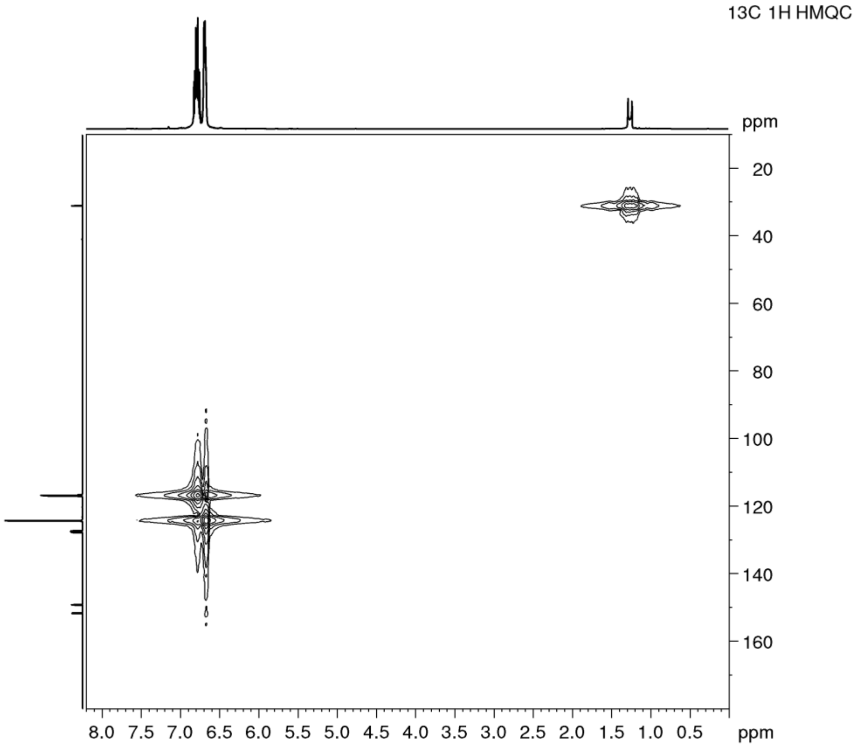


**Figure S5.** $^{13}C$ $^{1}H$ HMQC spectrum ($C_6D_6$) of **2[WCA]**

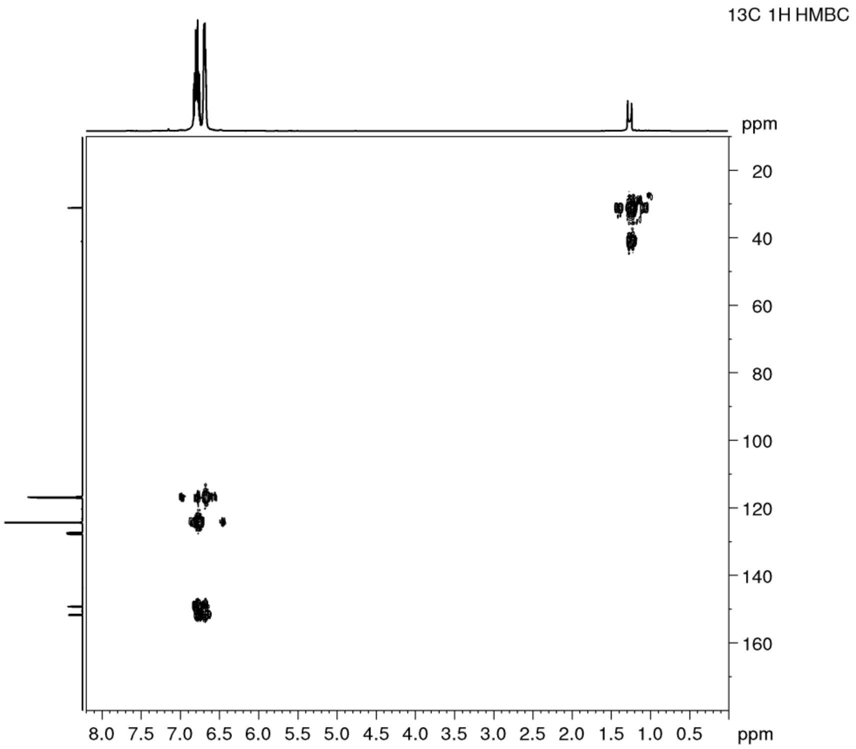


**Figure S6.** $^{13}C$ $^{1}H$ HMBC spectrum ($C_6D_6$) of **2[WCA]**

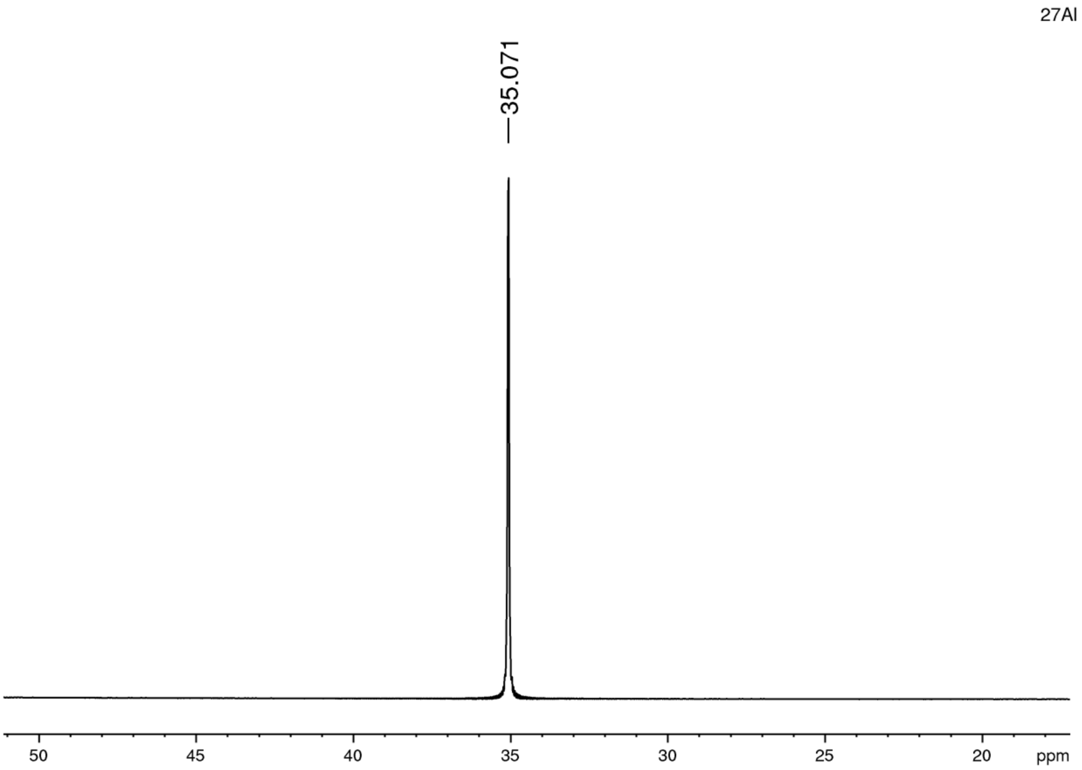


**Figure S7.** $^{27}Al$ spectrum ($C_6D_6$) of **2[WCA]**

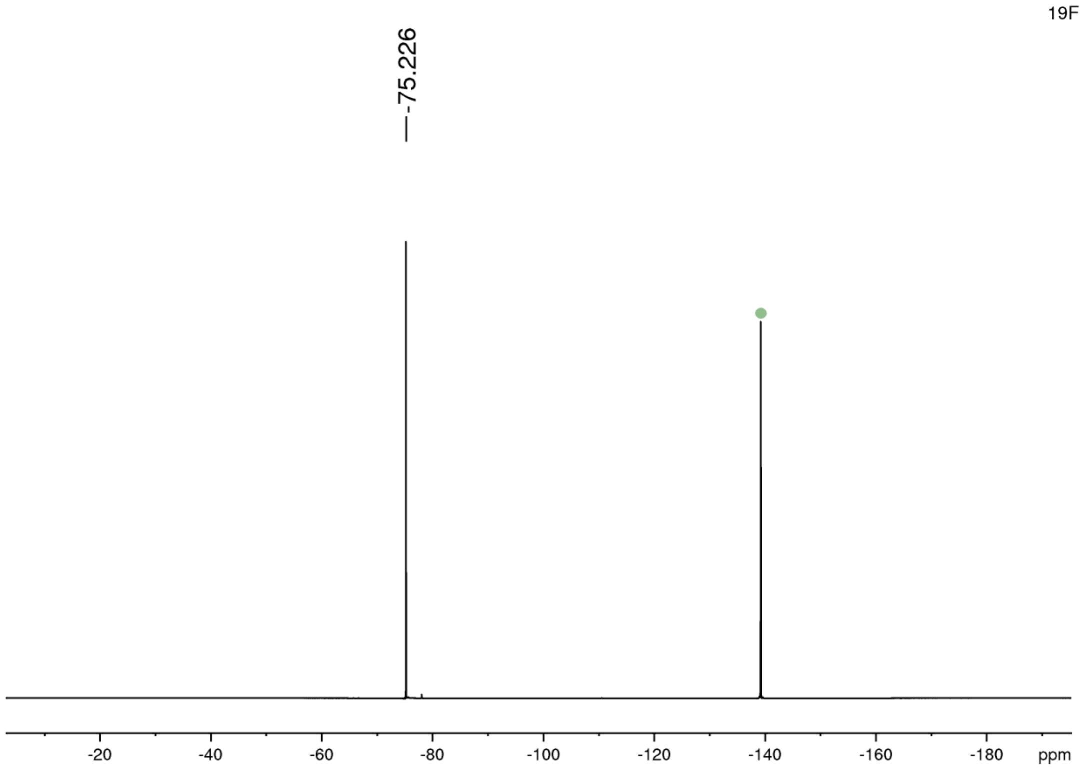


**Figure S8.** $^{19}F$ spectrum ($C_6D_6$) of **2[WCA]**
(● – 1,2-difluorobenzene)

# NMR spectra of reaction mixtures - reaction of 2[WCA] with $IMe_4$

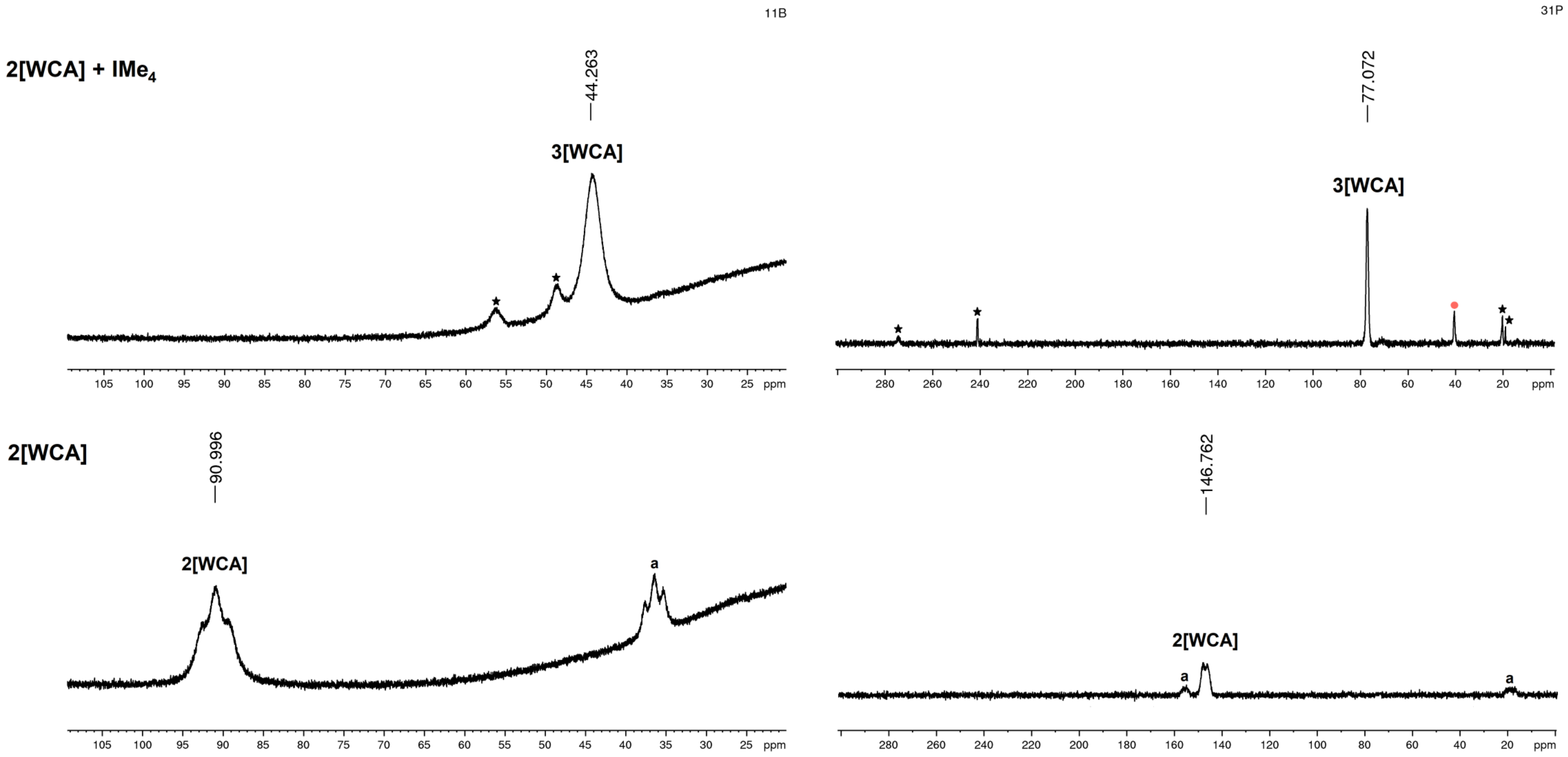


**Figure S9.** $^{11}$B and $^{31}$P NMR spectra ($C_6D_6$) of **2[WCA]** and **2[WCA] + NHC** reaction mixture – method A of **3[WCA]** synthesis
(**a** – adduct **1** with Li[WCA]; ★ – impurity; ● – *t*$Bu_2$P*Pt*$Bu_2$)

# NMR spectra of reaction mixtures - reaction of generated in situ 2[WCA] with $^{Me}$I*i*Pr$_2$

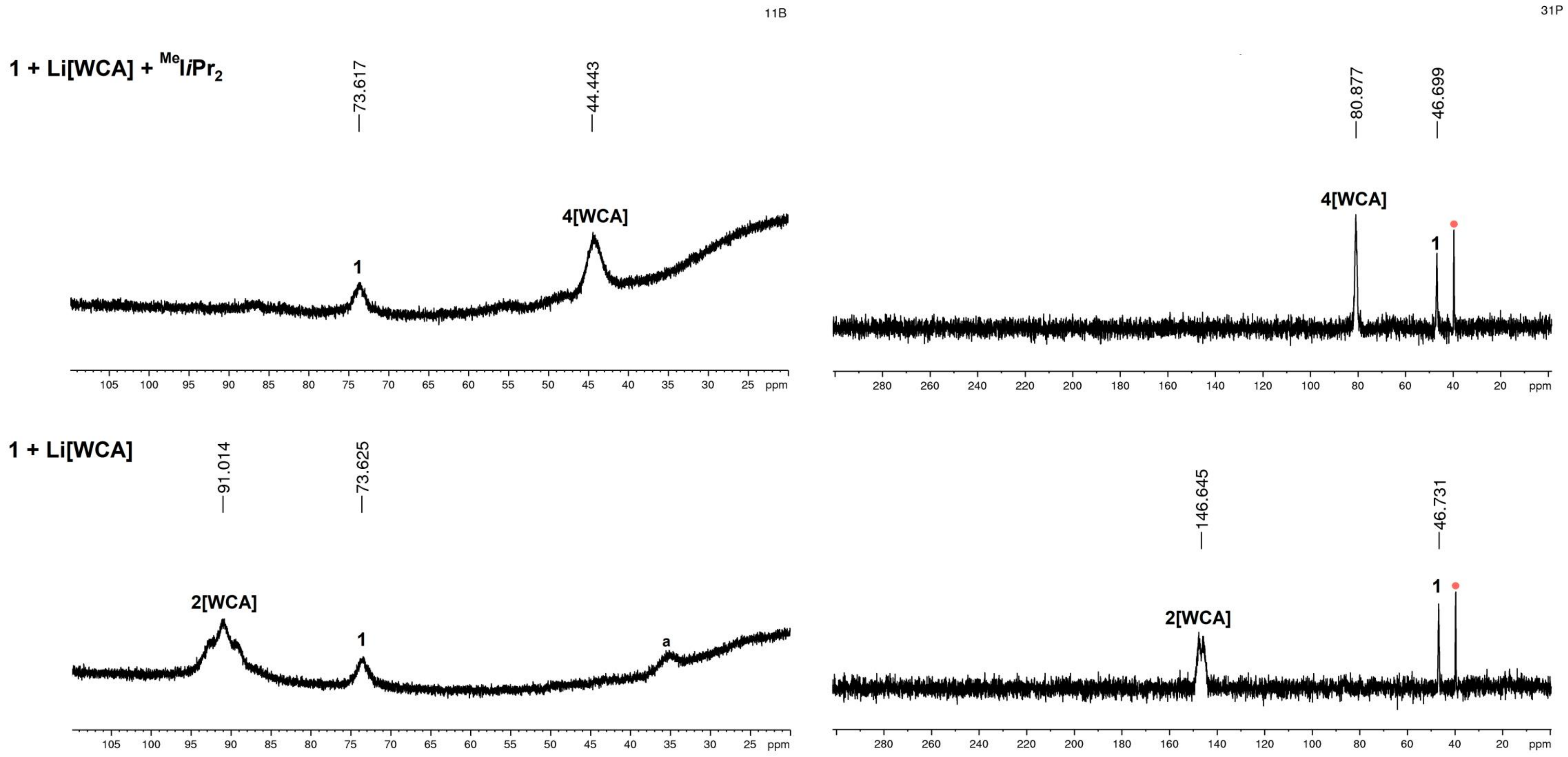


**Figure S10.** $^{11}$B and $^{31}$P NMR spectra ($C_6D_6$) of **1** + **Li[WCA]** and **1 + Li[WCA]** + **NHC** reaction mixtures – method A of **4[WCA]** synthesis (**a** – adduct **1** with Li[WCA]; ● – *t*Bu$_2$PP*t*Bu$_2$)

# NMR spectra of 3[WCA]

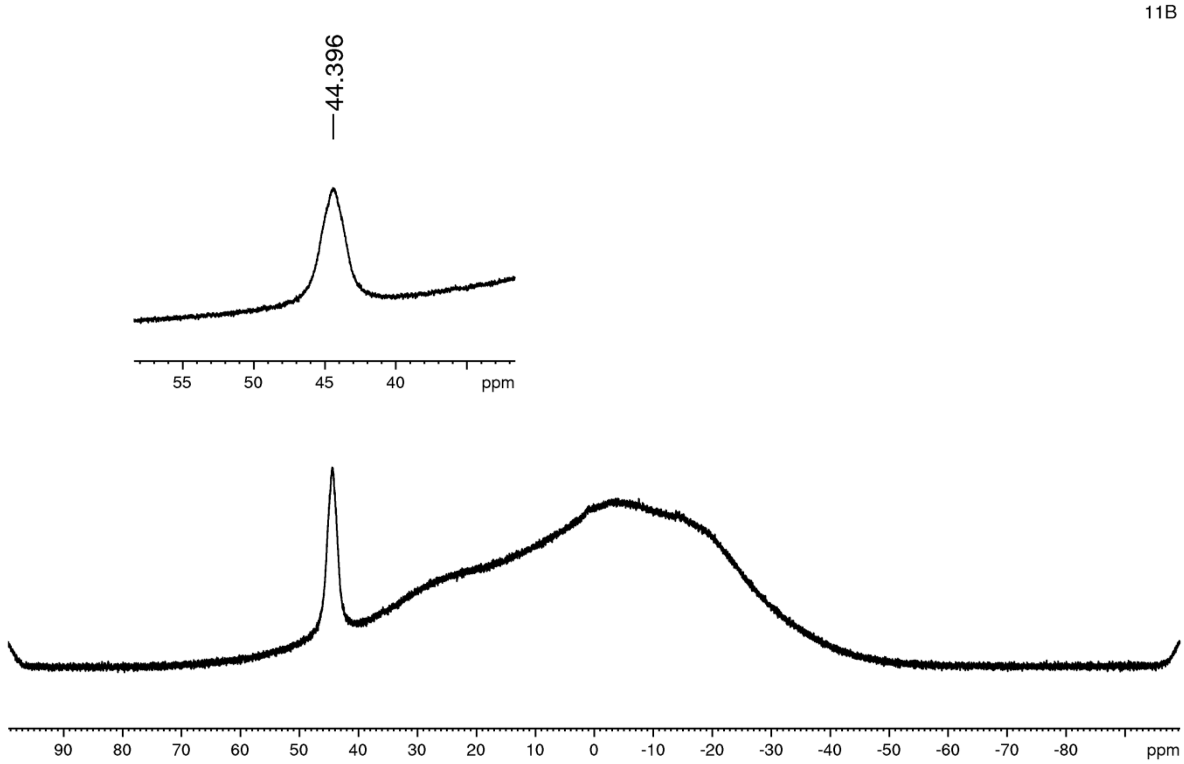


**Figure S11.** $^{11}B$ spectrum ($CD_2Cl_2$) of **3[WCA]**

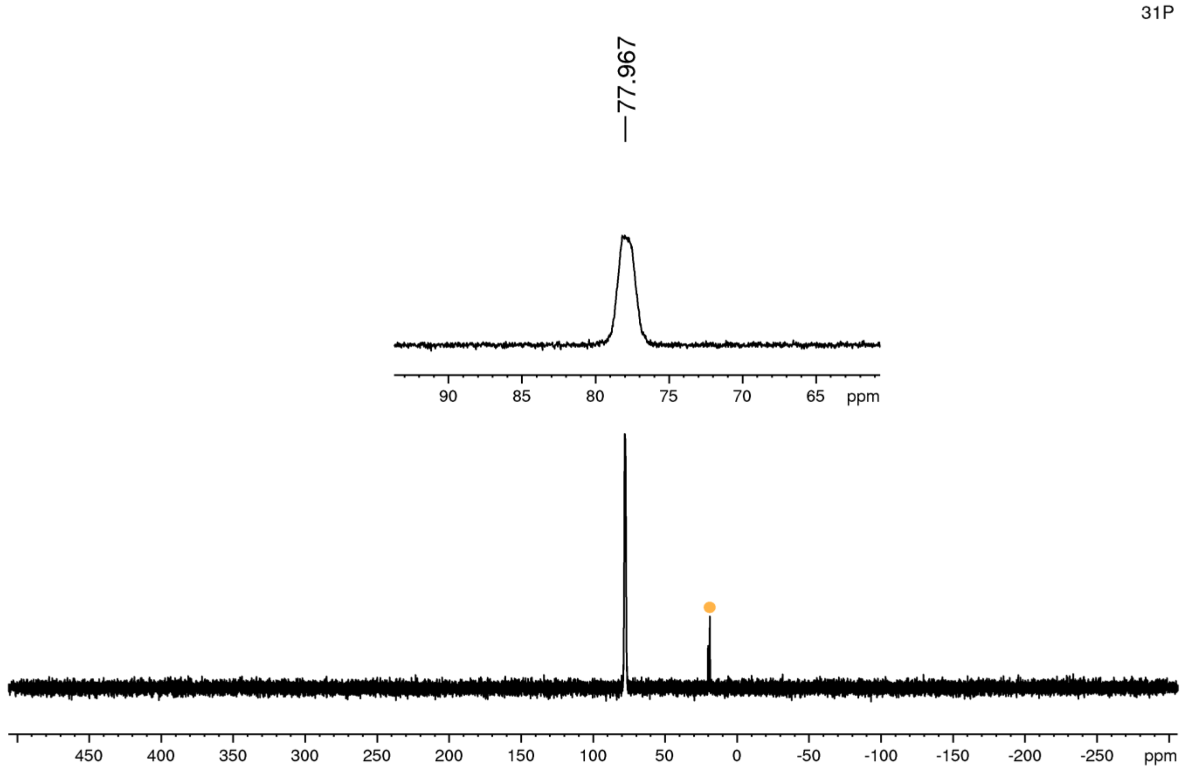


**Figure S12.** $^{31}P$ spectrum ($CD_2Cl_2$) of **3[WCA]**
(● – *t*$Bu_2PH$)

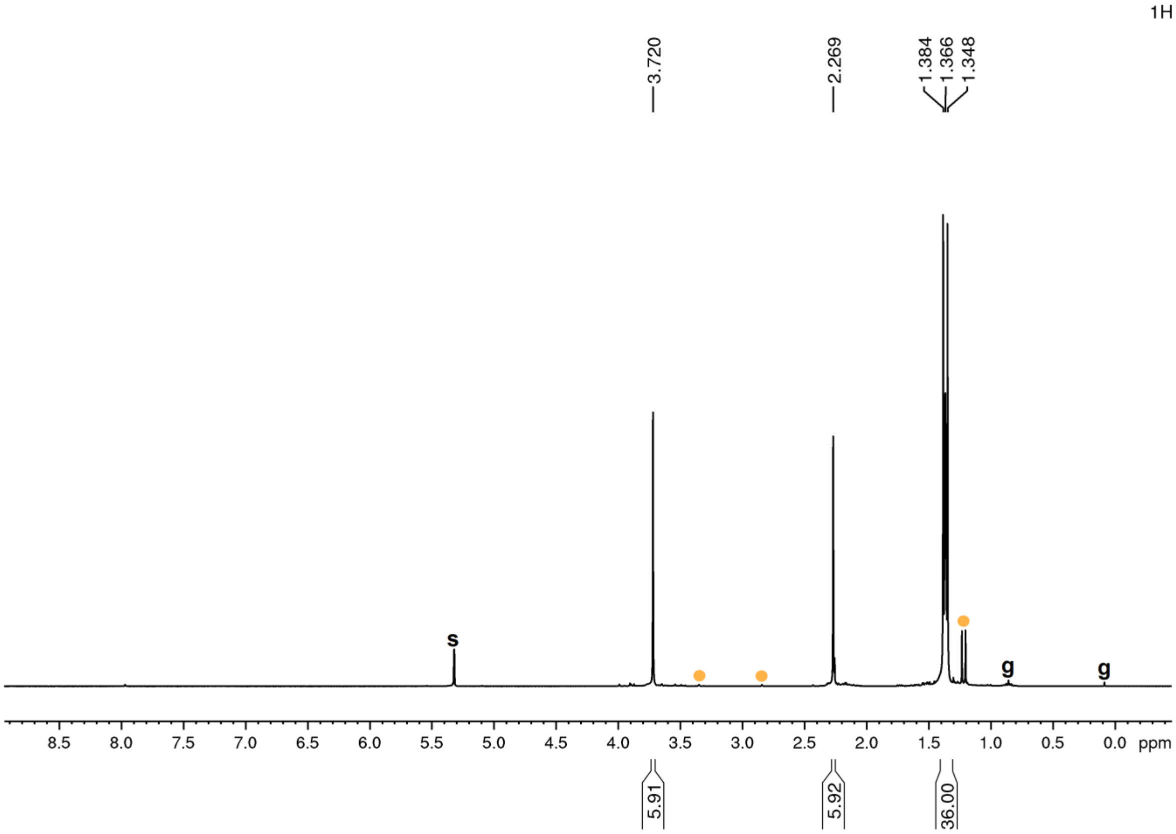


**Figure S13.** $^1$H spectrum ($CD_2Cl_2$) of **3[WCA]**
(**s** – deuterated solvent (residual signal); **g** – grease; ● – *t*$Bu_2$PH)

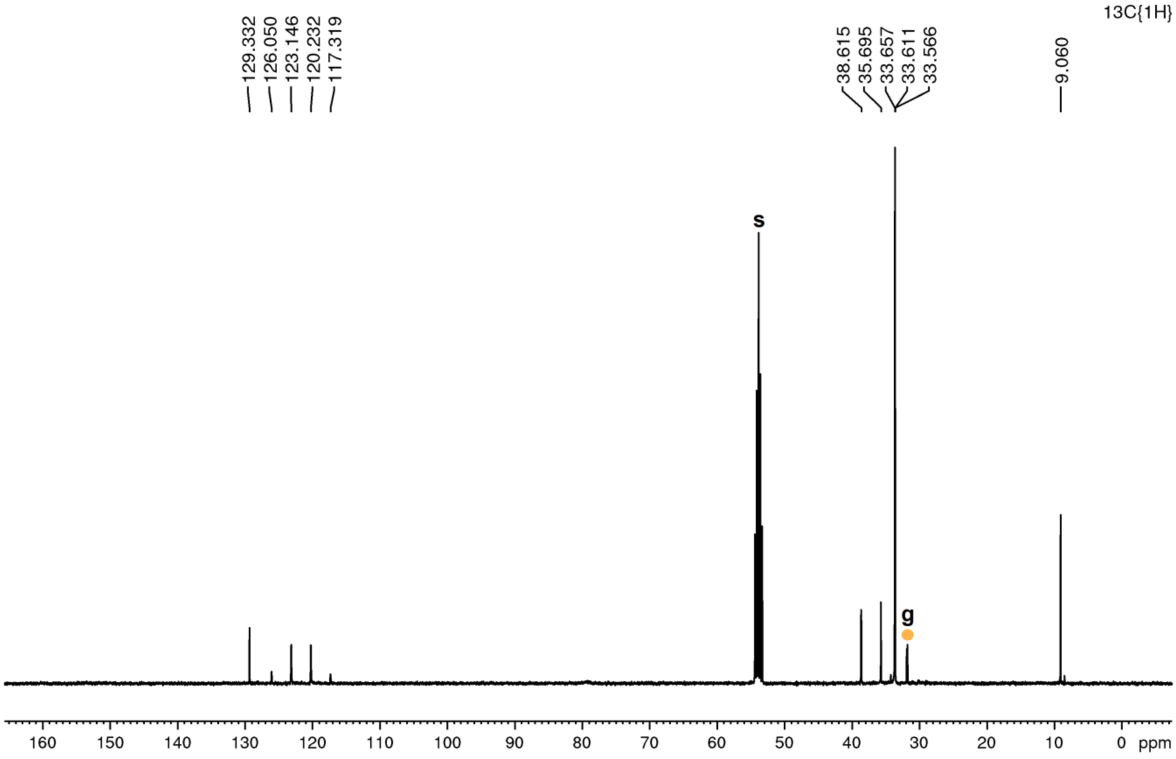


**Figure S14.** $^{13}C\{^1H\}$ spectrum ($CD_2Cl_2$) of **3[WCA]**
(**s** – deuterated solvent (residual signal); **g** – grease; ● – *t*$Bu_2$PH)

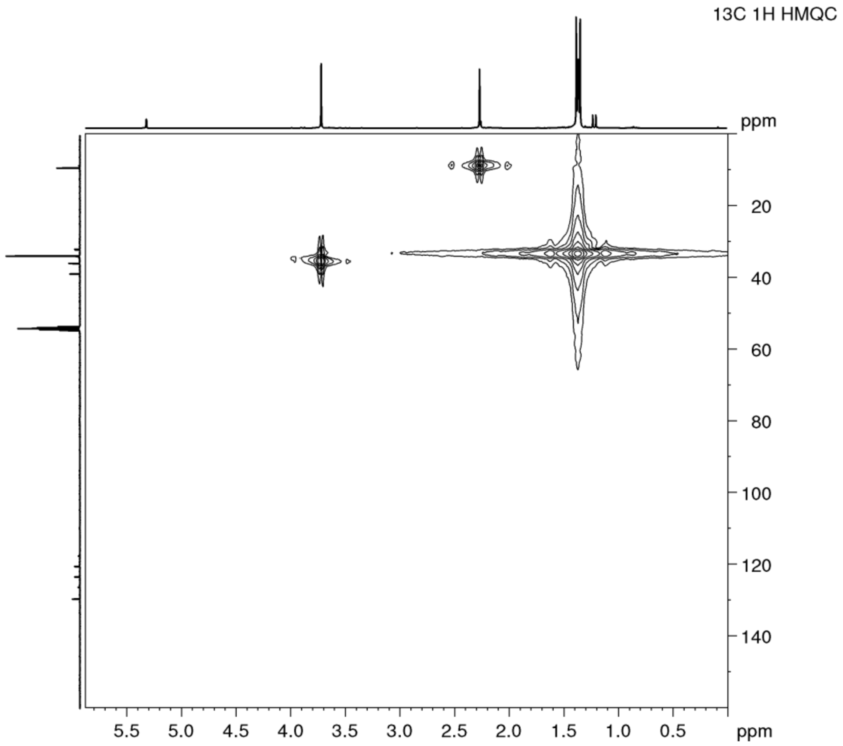


**Figure S15.** $^{13}C$ $^{1}H$ HMQC spectrum ($CD_2Cl_2$) of **3[WCA]**

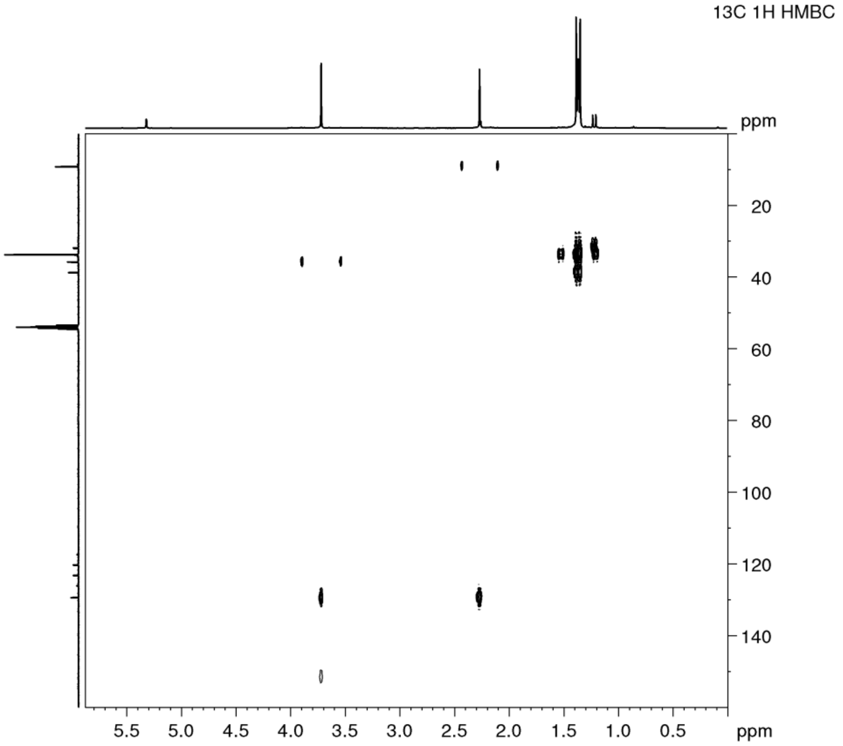


**Figure S16.** $^{13}C$ $^{1}H$ HMBC spectrum ($CD_2Cl_2$) of **3[WCA]**

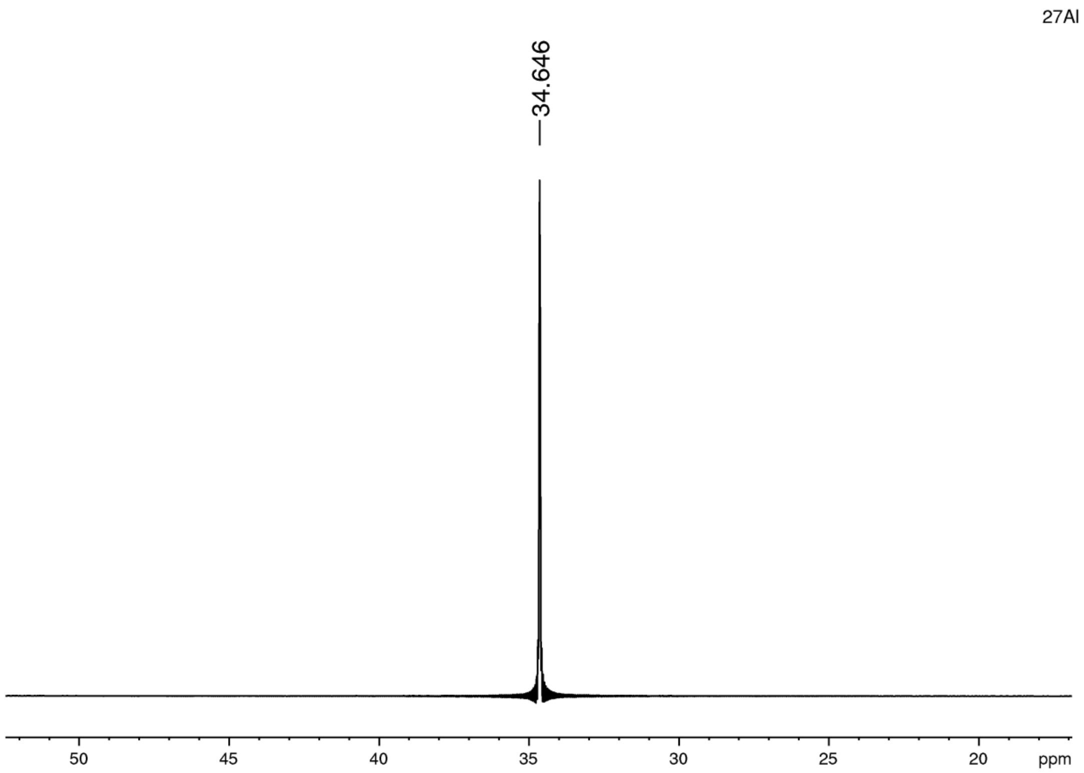


**Figure S17.** $^{27}$Al spectrum ($CD_2Cl_2$) of **3[WCA]**

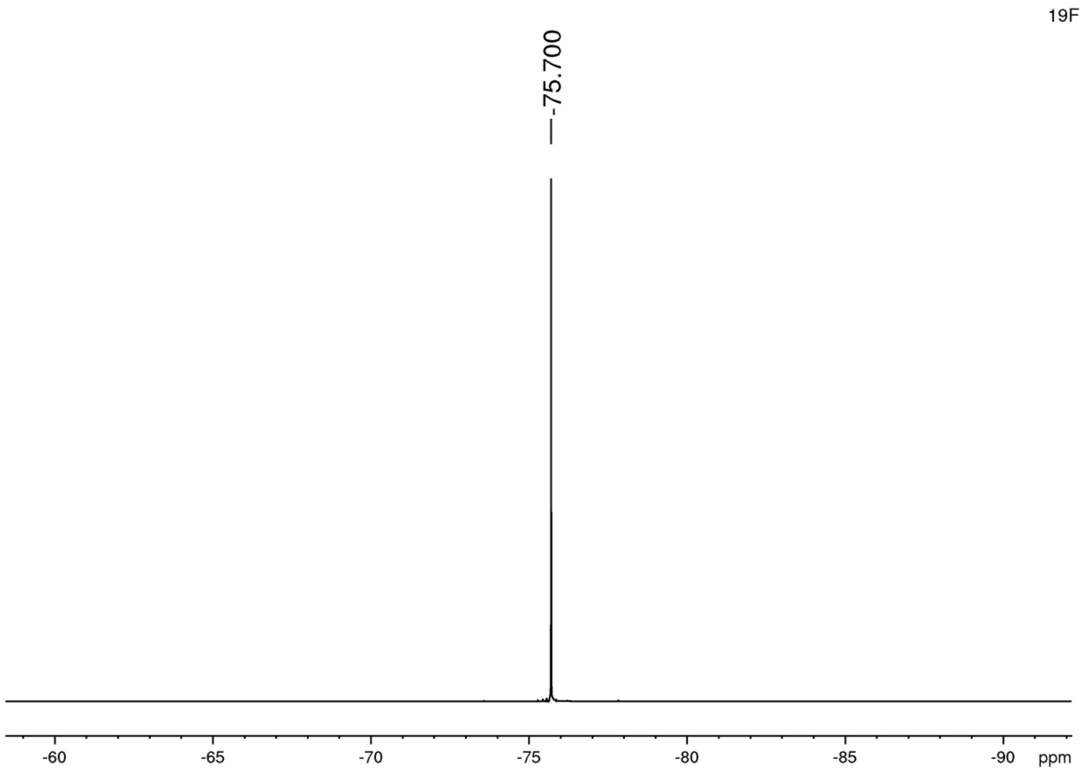


**Figure S18.** $^{19}$F spectrum ($CD_2Cl_2$) of **3[WCA]**

# NMR spectra of 4[WCA]

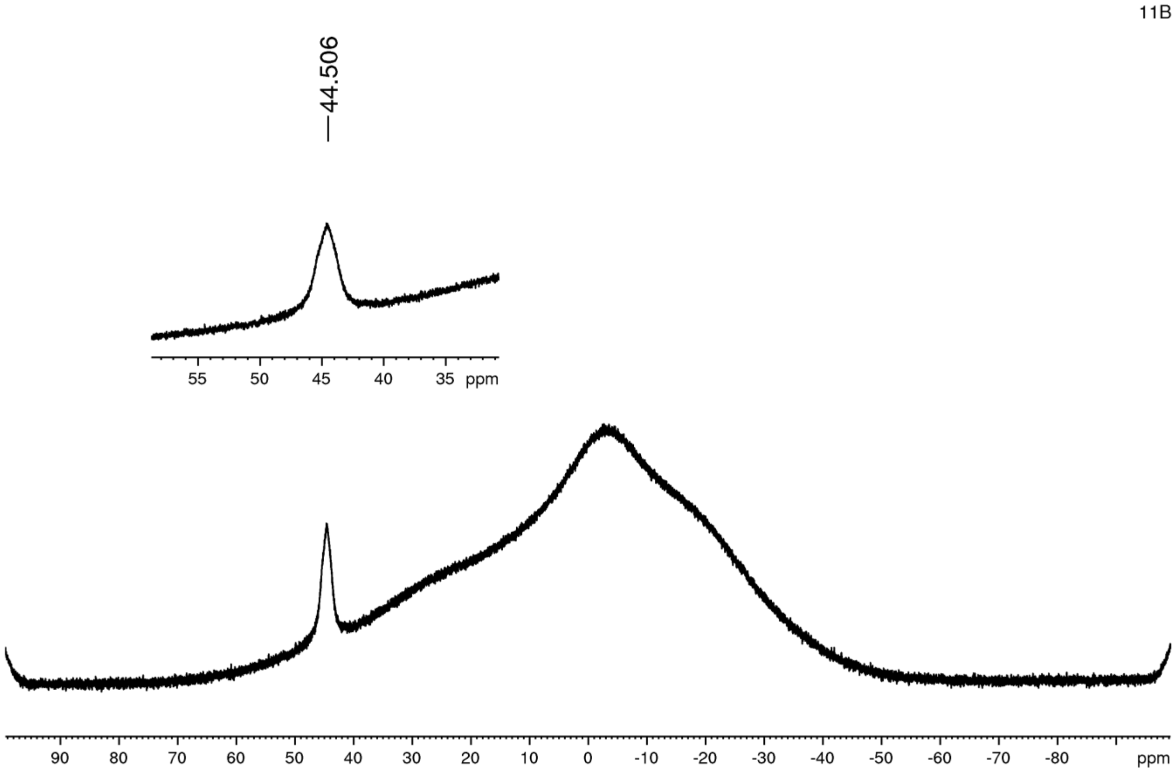


**Figure S19.** $^{11}B$ spectrum ($CD_2Cl_2$) of **4[WCA]**

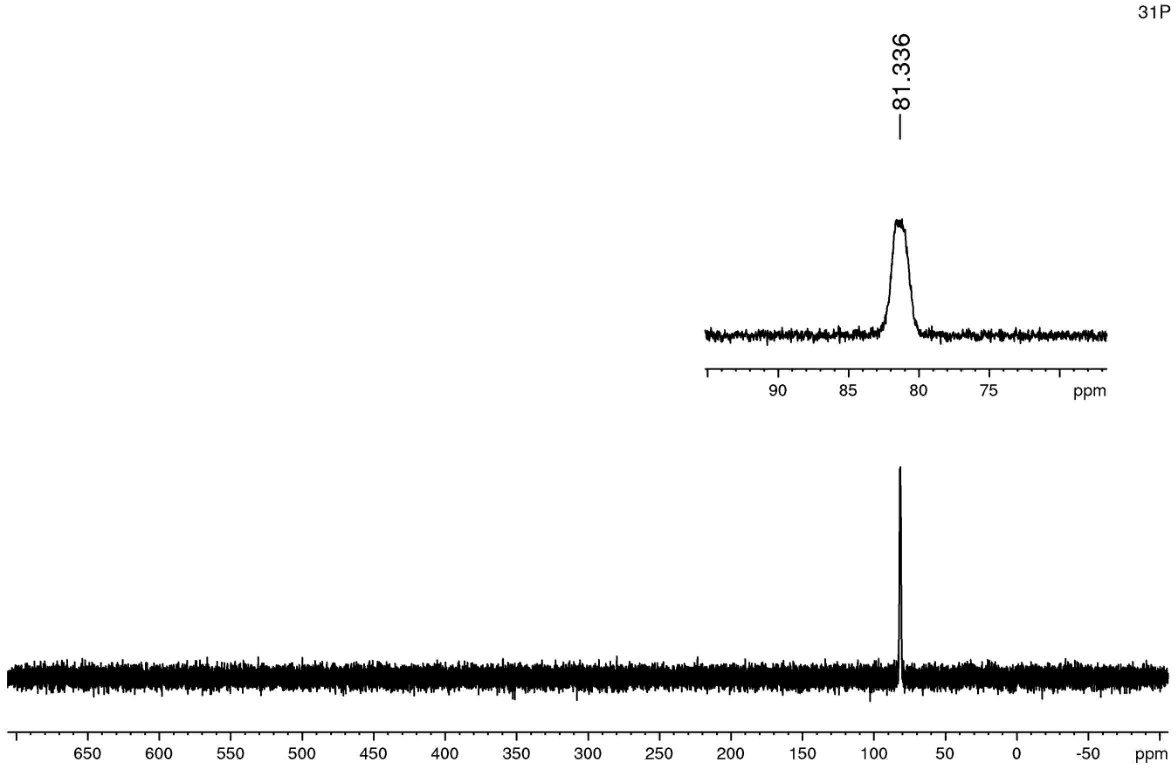


**Figure S20.** $^{31}P$ spectrum ($CD_2Cl_2$) of **4[WCA]**

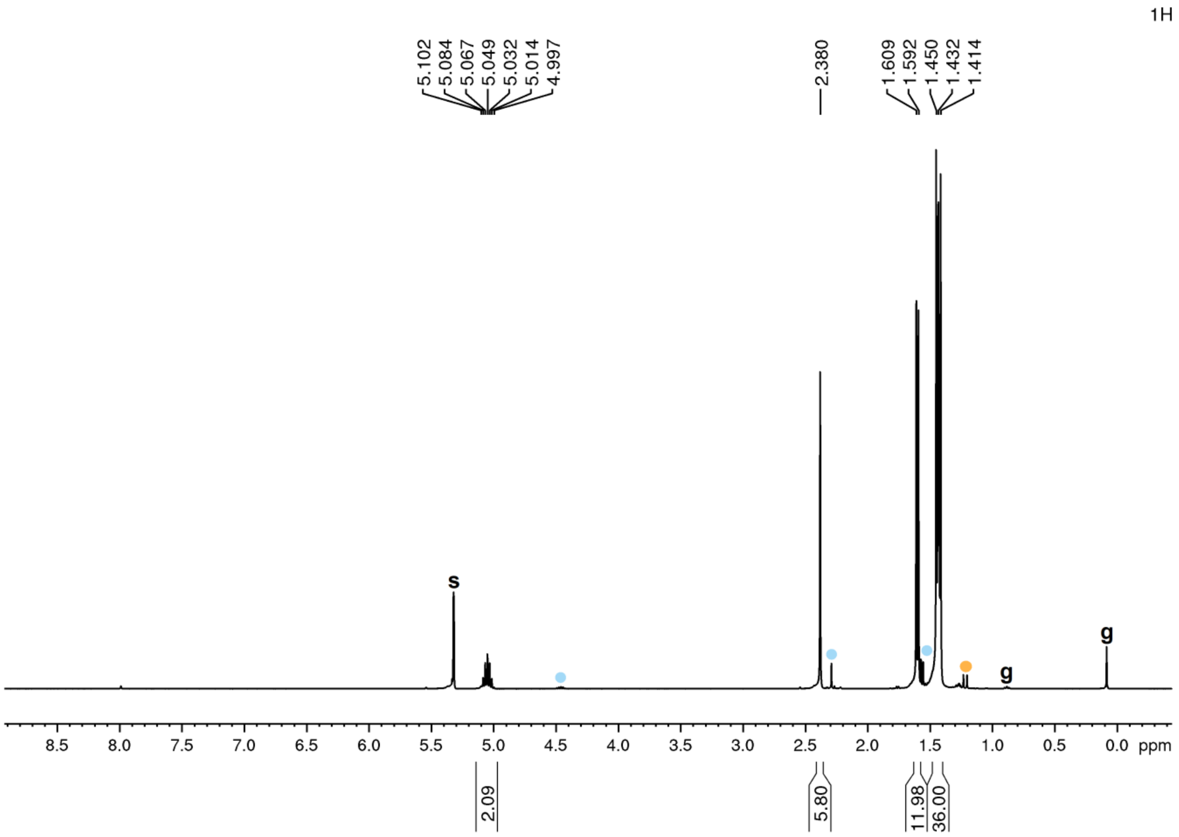


**Figure S21.** $^{1}H$ spectrum ($CD_2Cl_2$) of **4[WCA]**
(**s** – deuterated solvent (residual signal); **g** – grease; 🟠 – *t*$Bu_2PH$; 🔵 – imidazolium salt)

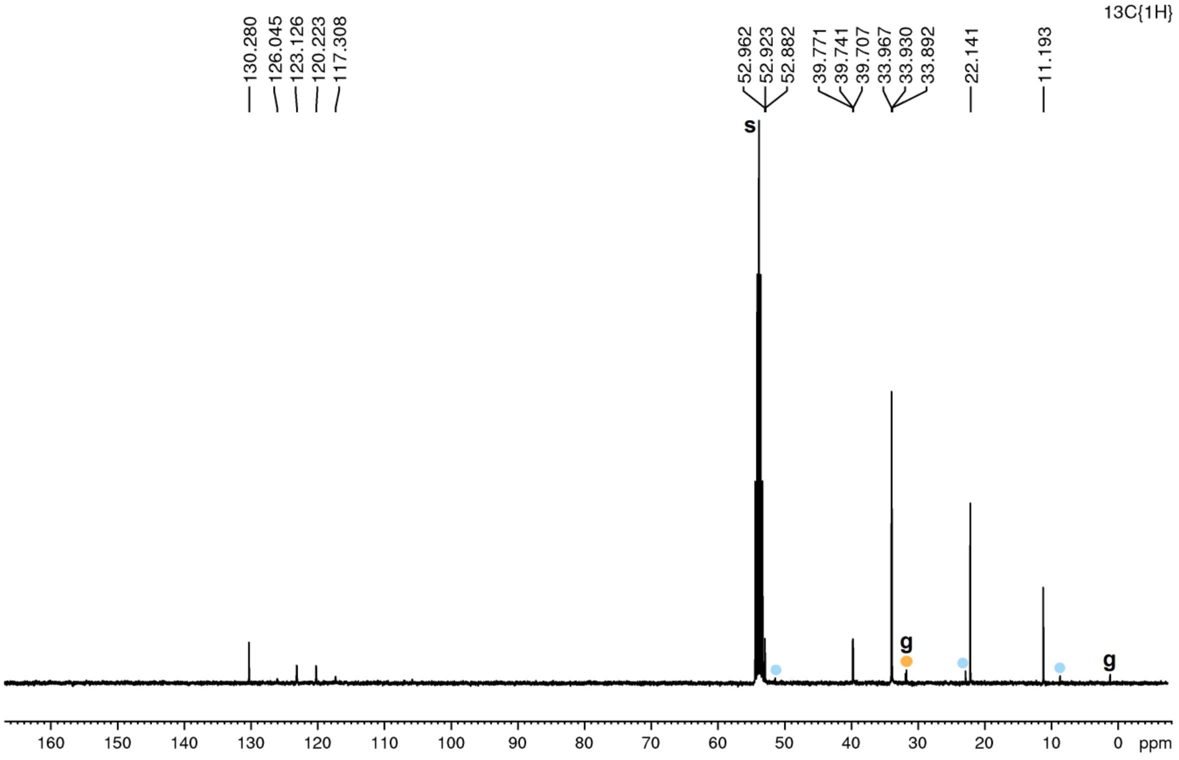


**Figure S22.** $^{13}C\{^{1}H\}$ spectrum ($CD_2Cl_2$) of **4[WCA]**
(**s** – deuterated solvent (residual signal); **g** – grease; 🟠 – *t*$Bu_2PH$; 🔵 – imidazolium salt)

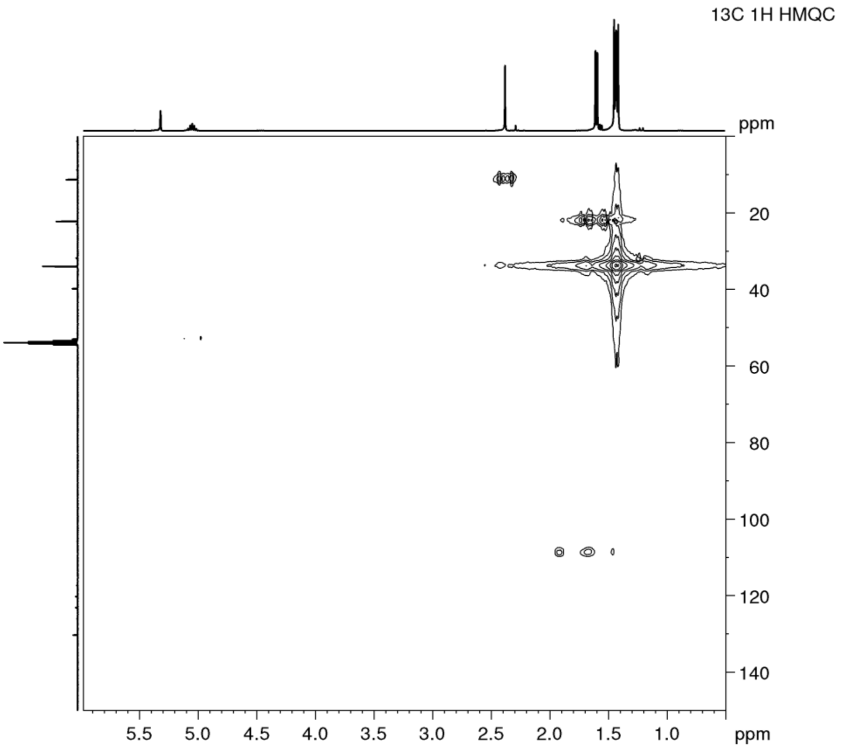


**Figure S23.** $^{13}C$ $^{1}H$ HMQC spectrum ($CD_2Cl_2$) of **4[WCA]**

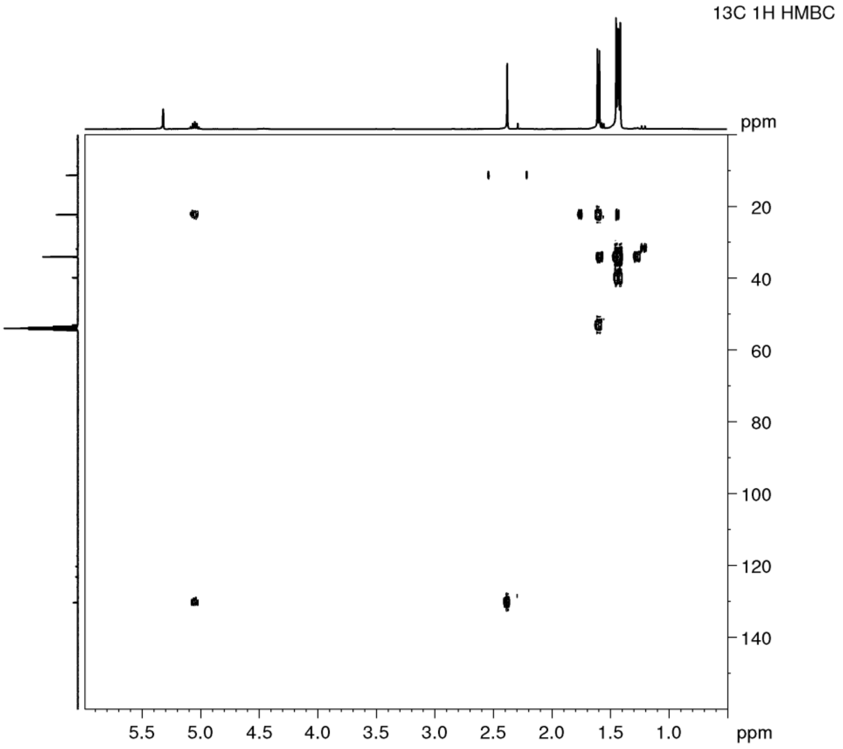


**Figure S24.** $^{13}C$ $^{1}H$ HMBC spectrum ($CD_2Cl_2$) of **4[WCA]**

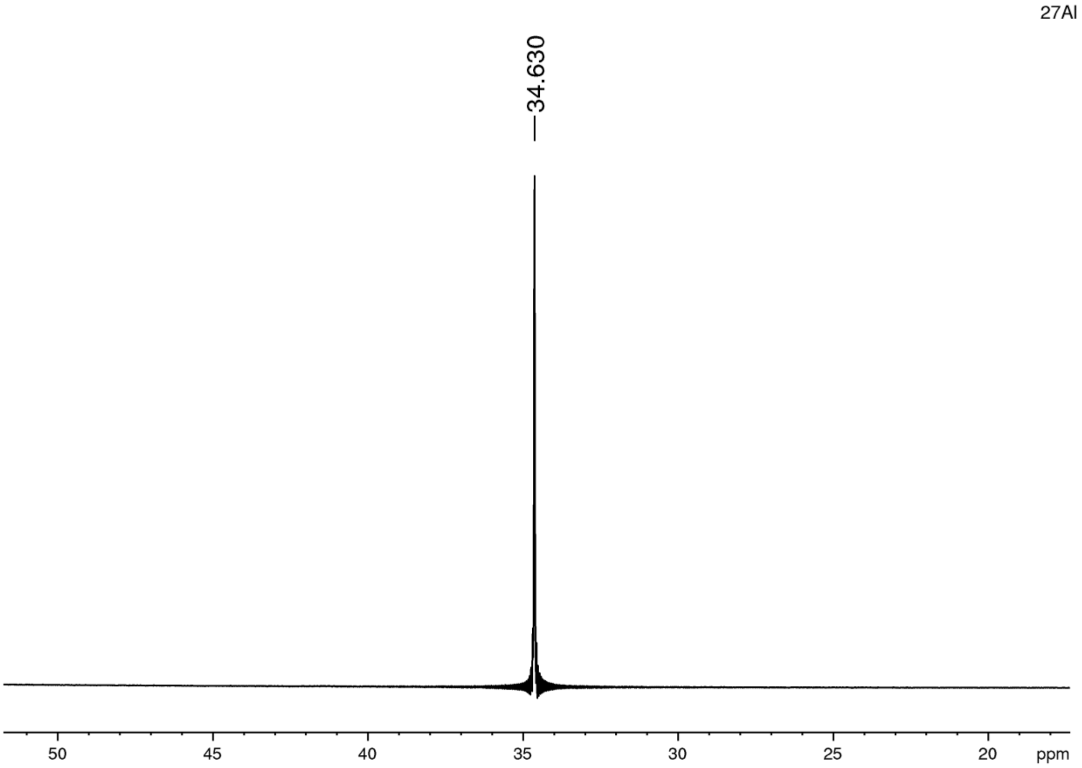


**Figure S25.** $^{27}$Al spectrum ($CD_2Cl_2$) of **4[WCA]**

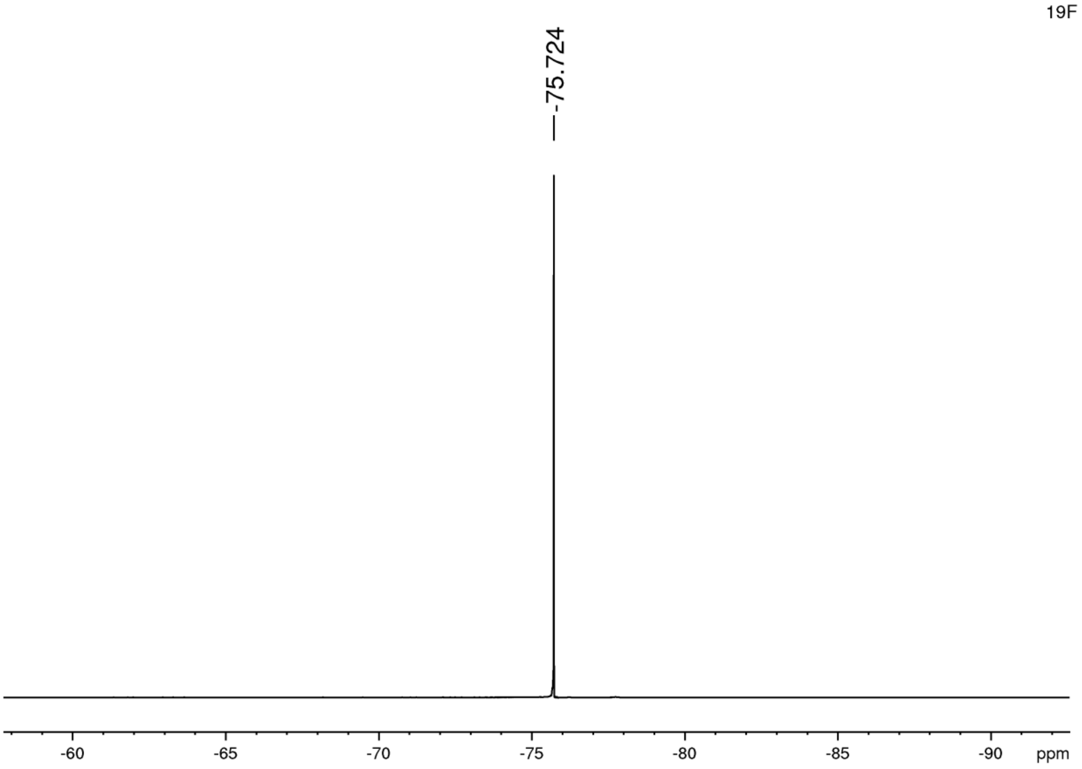


**Figure S26.** $^{19}$F spectrum ($CD_2Cl_2$) of **4[WCA]**

# NMR spectra of 5[WCA] reaction mixture

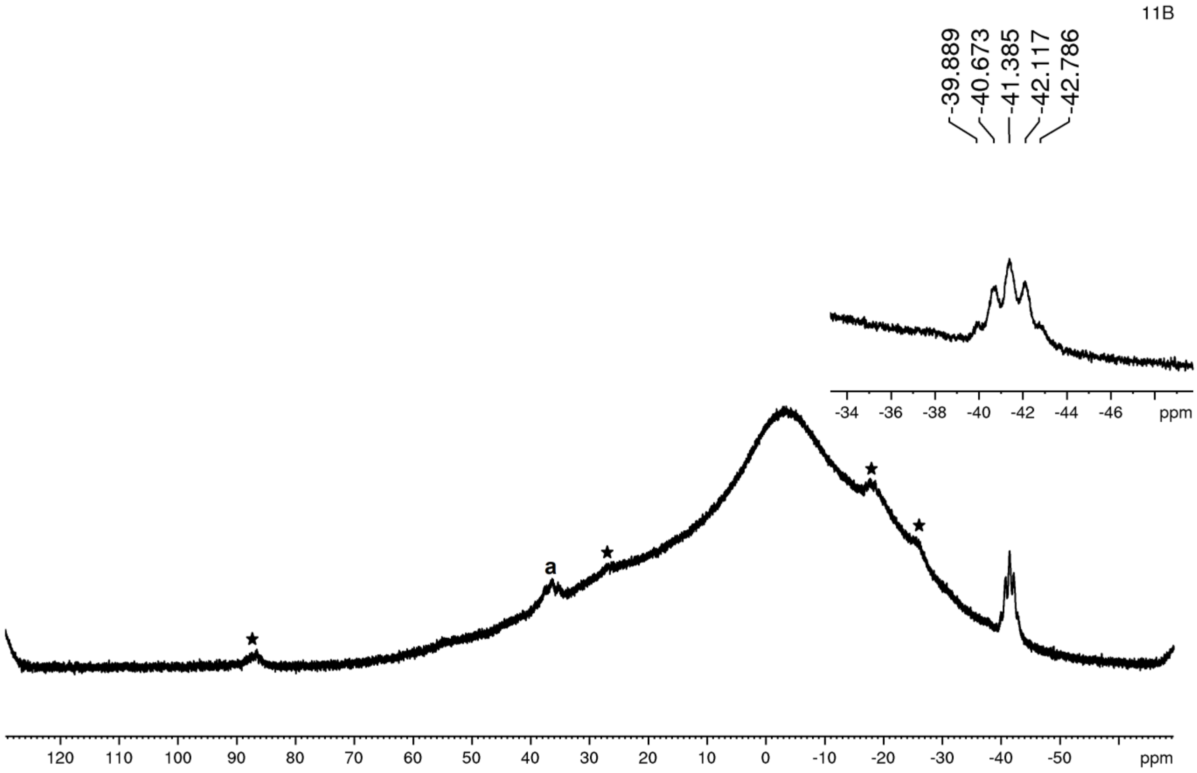


**Figure S27.** $^{11}B$ spectrum ($C_6D_6$) of **5[WCA]** reaction mixture
(**a** – adduct **1** with Li[WCA]; ★ – impurity)

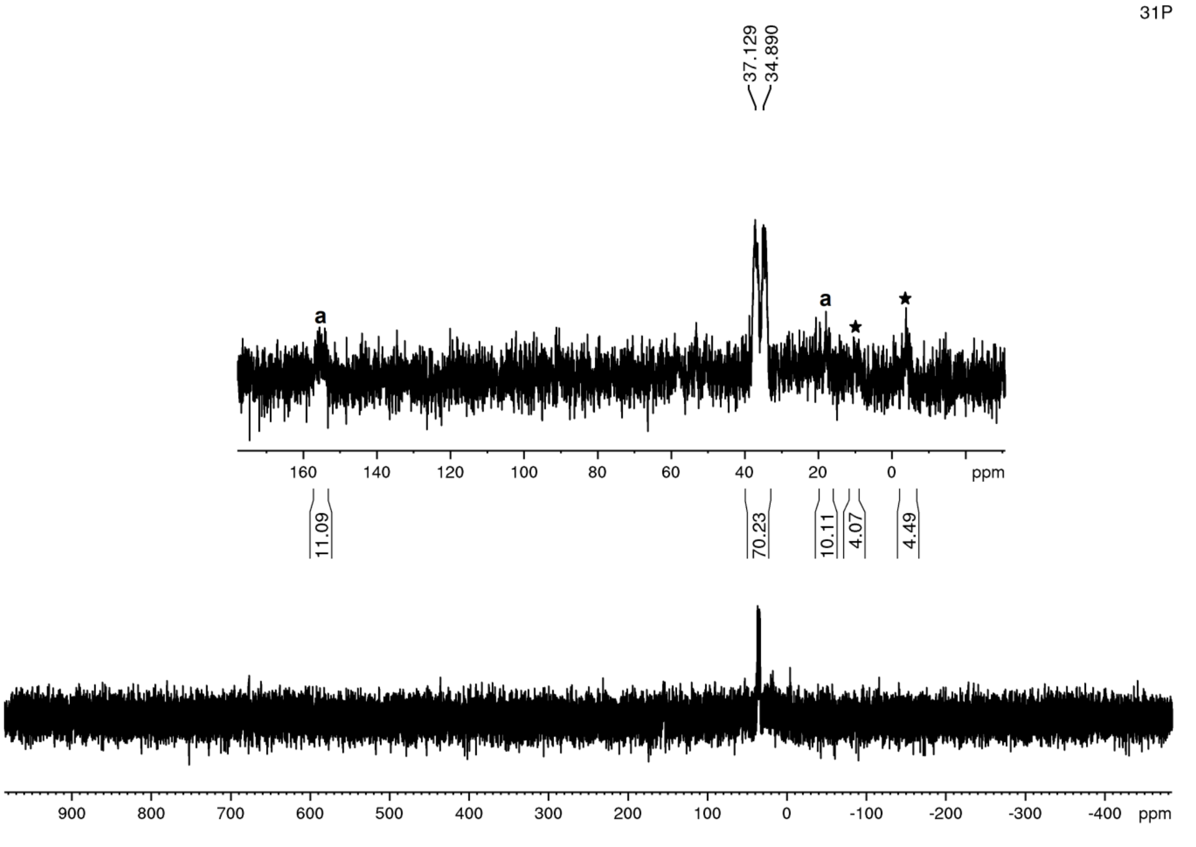


**Figure S28.** $^{31}P$ spectrum ($C_6D_6$) of **5[WCA]** reaction mixture
(**a** – adduct **1** with Li[WCA]; ★ – impurity)

## NMR spectra of 5[WCA]

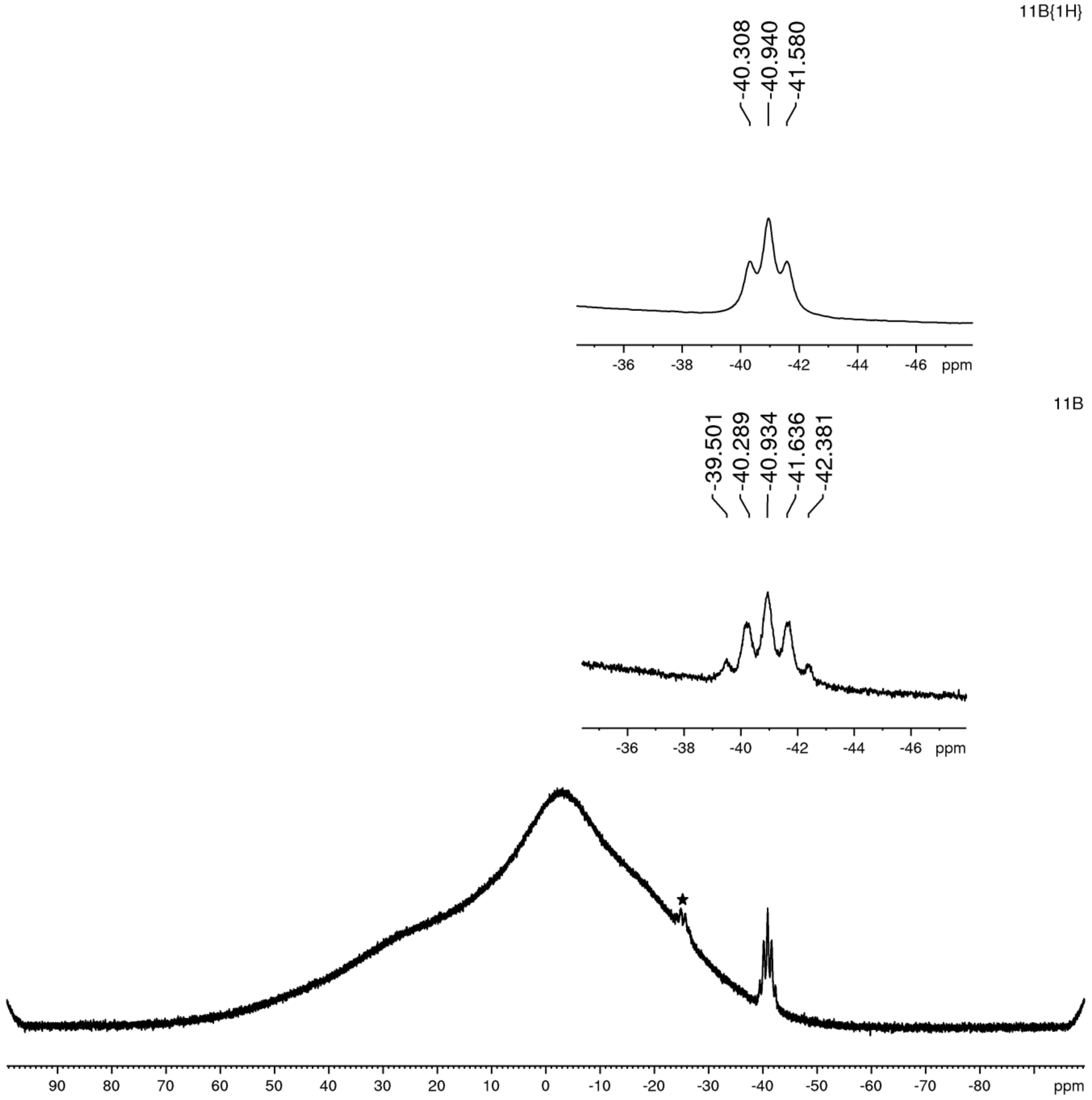


**Figure S29.** $^{11}B\{^{1}H\}$ and $^{11}B$ spectra ($CD_2Cl_2$) of **5[WCA]**
(★ – impurity)

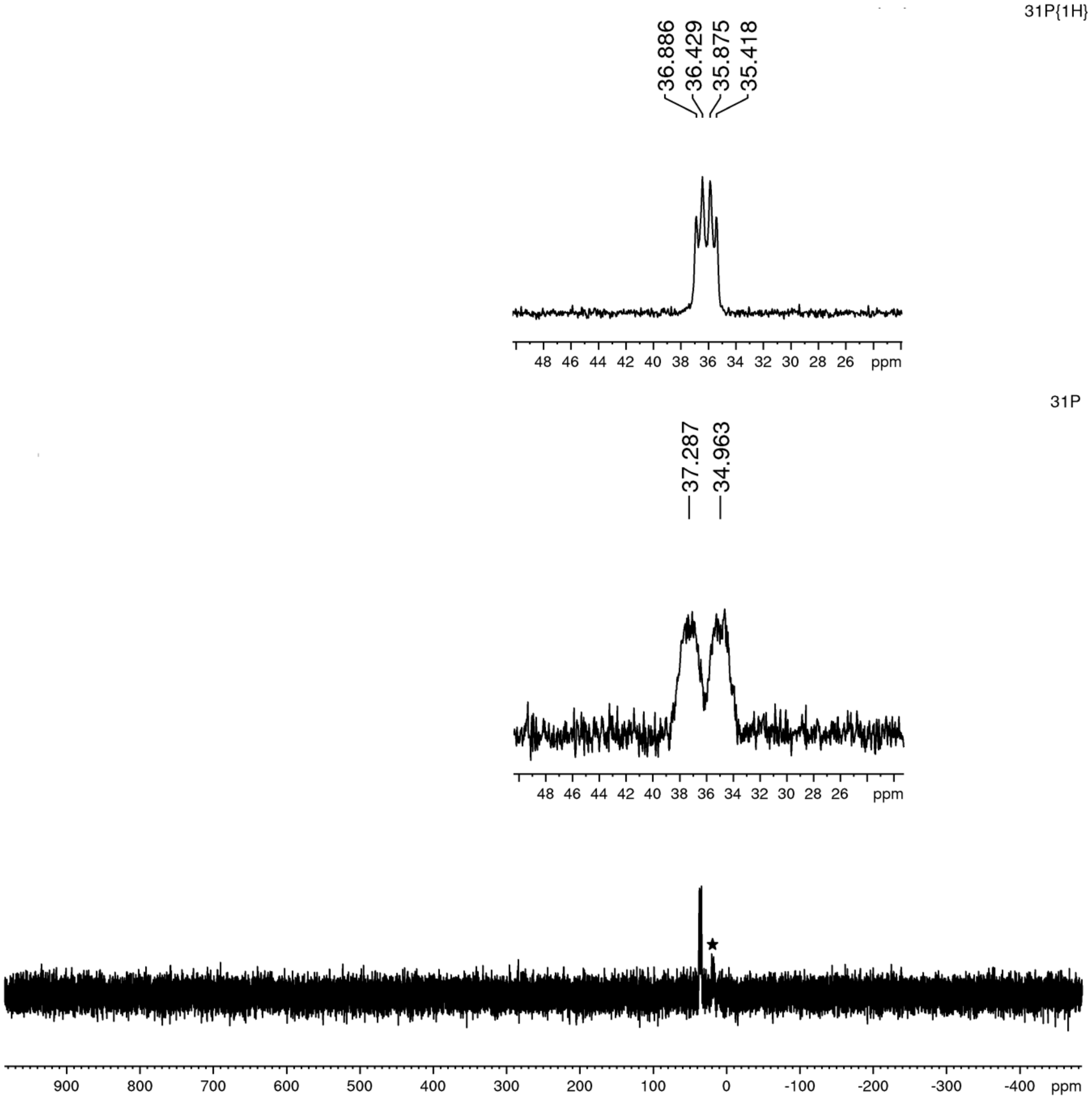


**Figure S30.** $^{31}P\{^{1}H\}$ and $^{31}P$ spectrum ($CD_2Cl_2$) of **5[WCA]**
(★ – impurity)

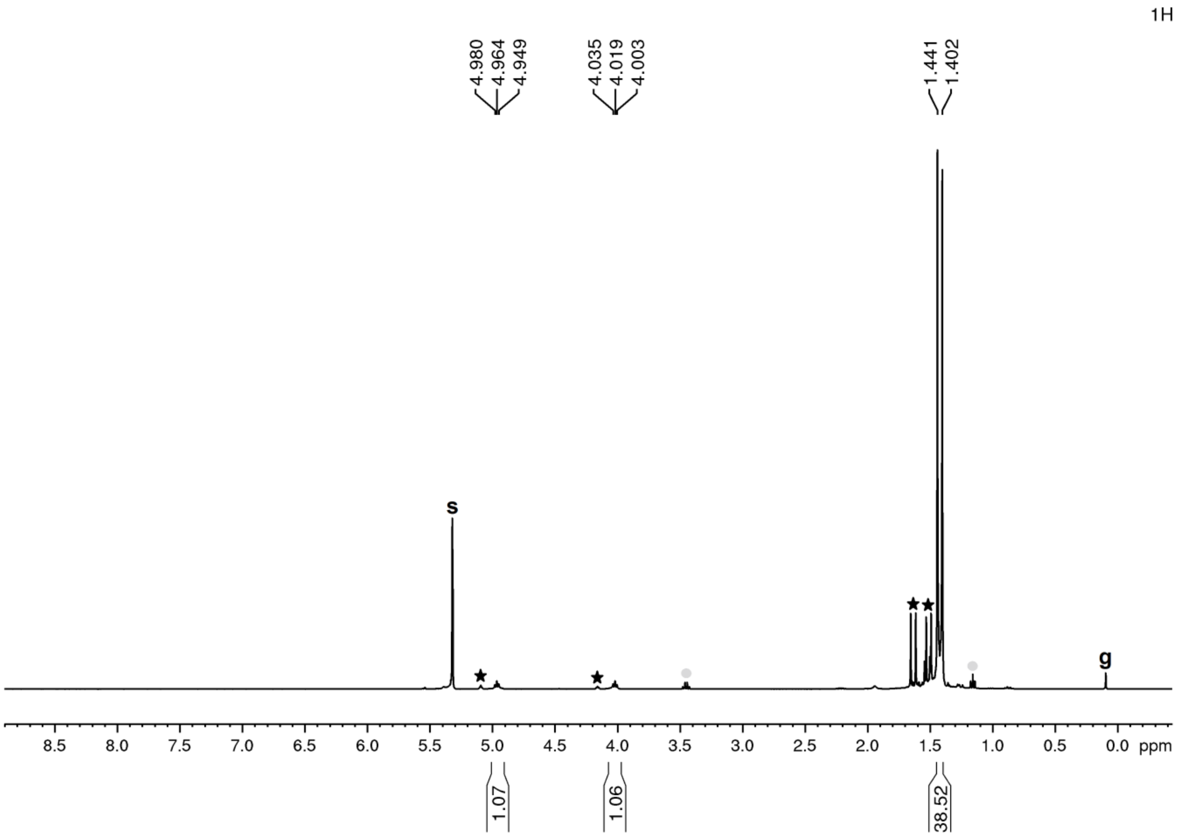


**Figure S31.** $^{1}H$ spectrum ($CD_2Cl_2$) of **5[WCA]**
(**s** – deuterated solvent (residual signal); **g** – grease; ★ – impurity; ● – $Et_2O$)

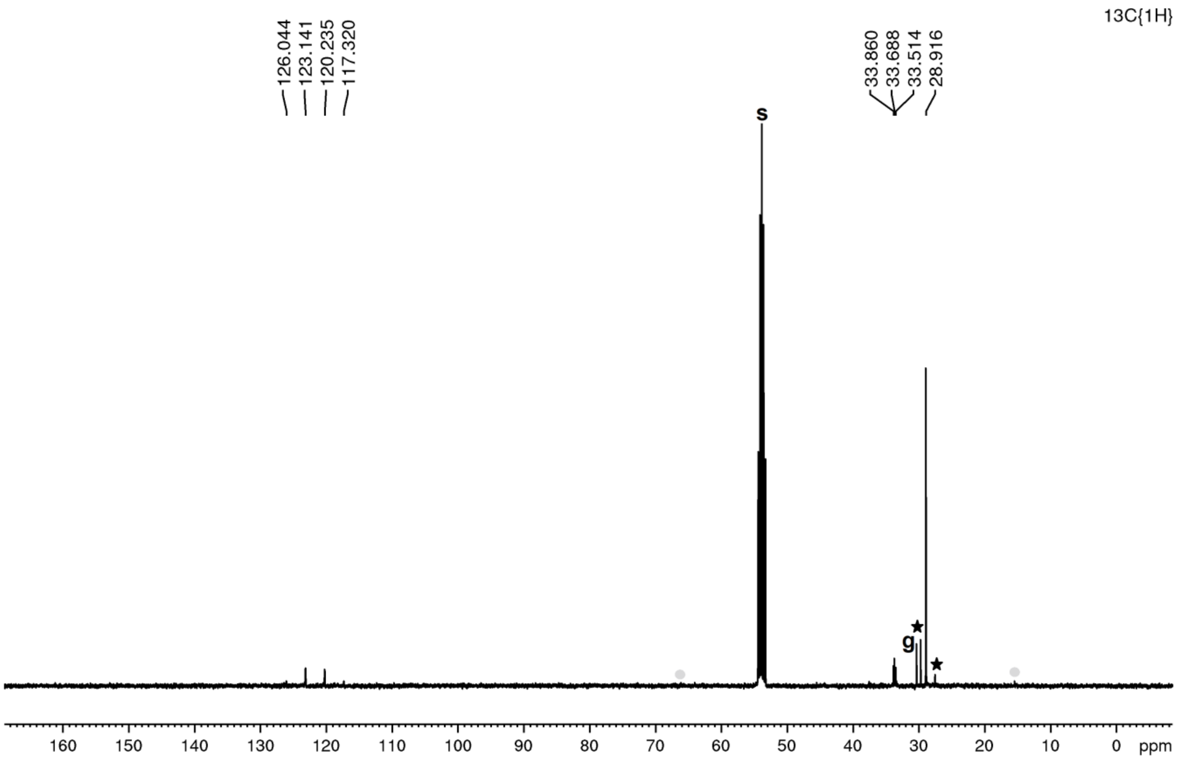


**Figure S32.** $^{13}C\{^{1}H\}$ spectrum ($CD_2Cl_2$) of **5[WCA]**
(**s** – deuterated solvent (residual signal); **g** – grease; ★ – impurity; ● – $Et_2O$)

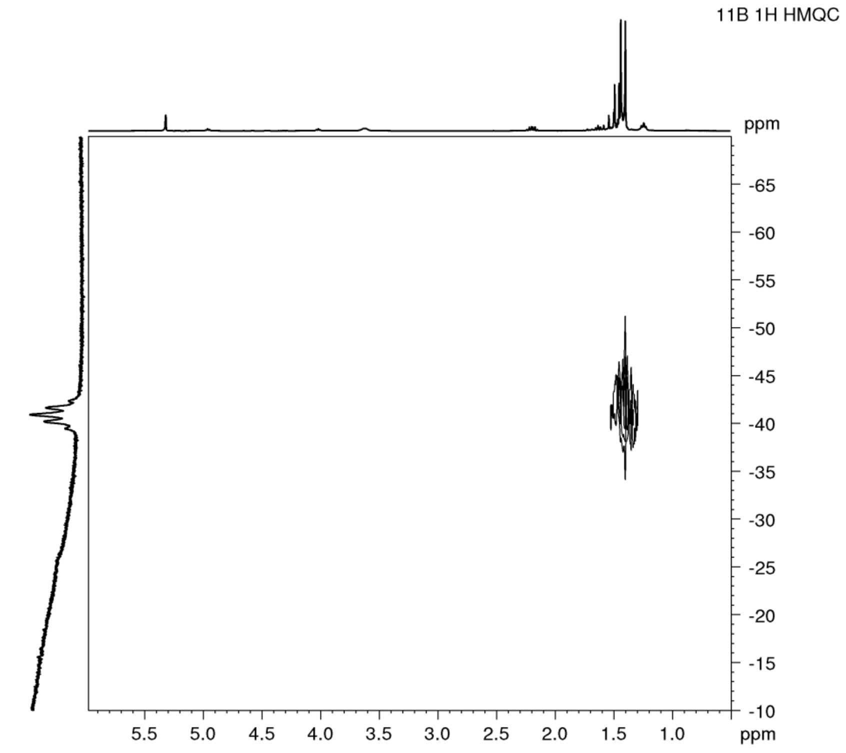


**Figure S33.** $^{11}B$ $^{1}H$ HMQC spectrum ($CD_2Cl_2$) of **5[WCA]**

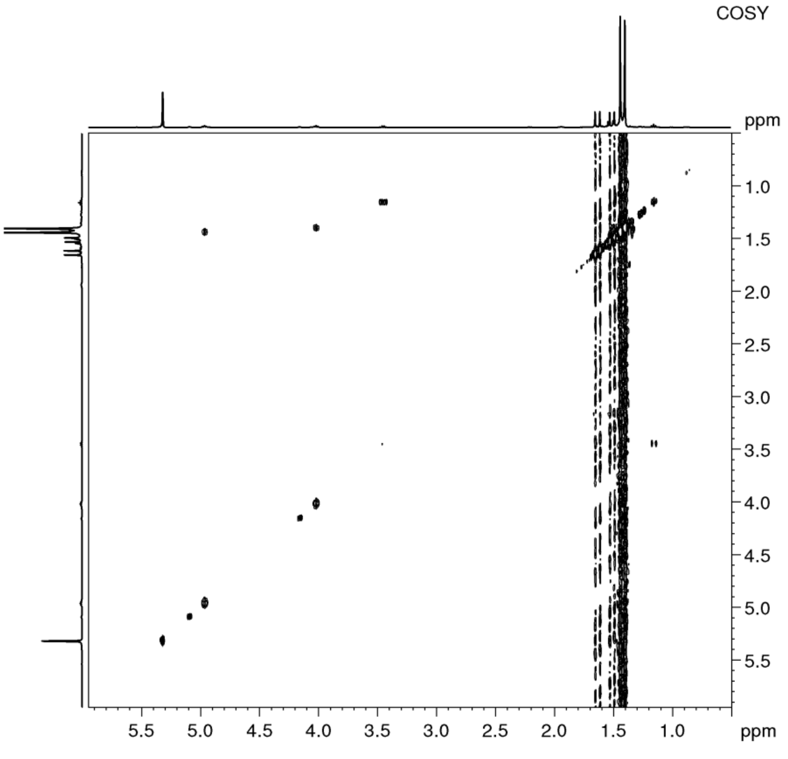


**Figure S34.** COSY spectrum ($CD_2Cl_2$) of **5[WCA]**

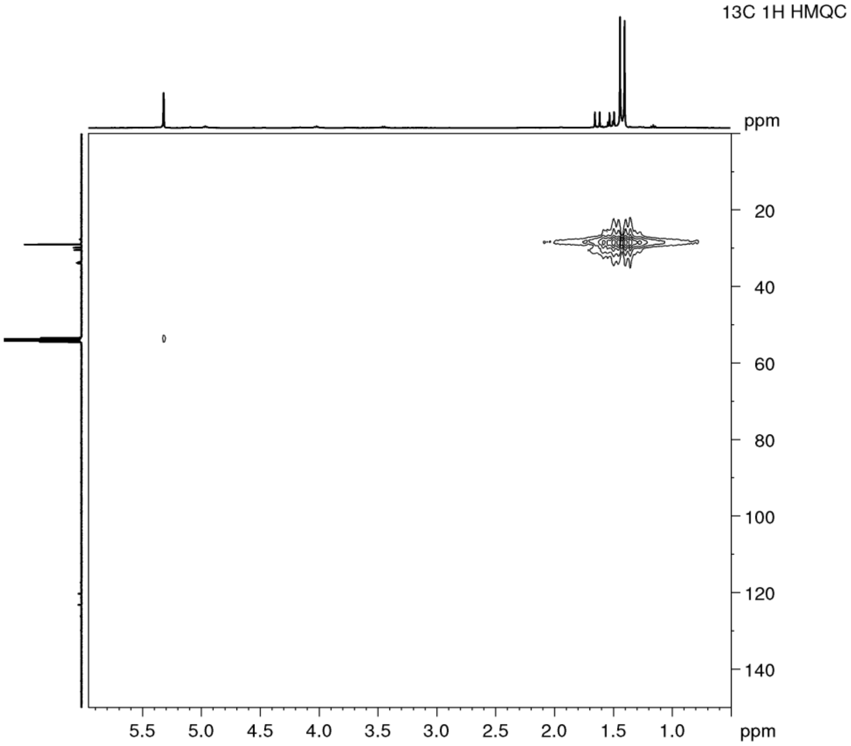


**Figure S35.** $^{13}C$ $^{1}H$ HMQC spectrum ($CD_2Cl_2$) of **5[WCA]**

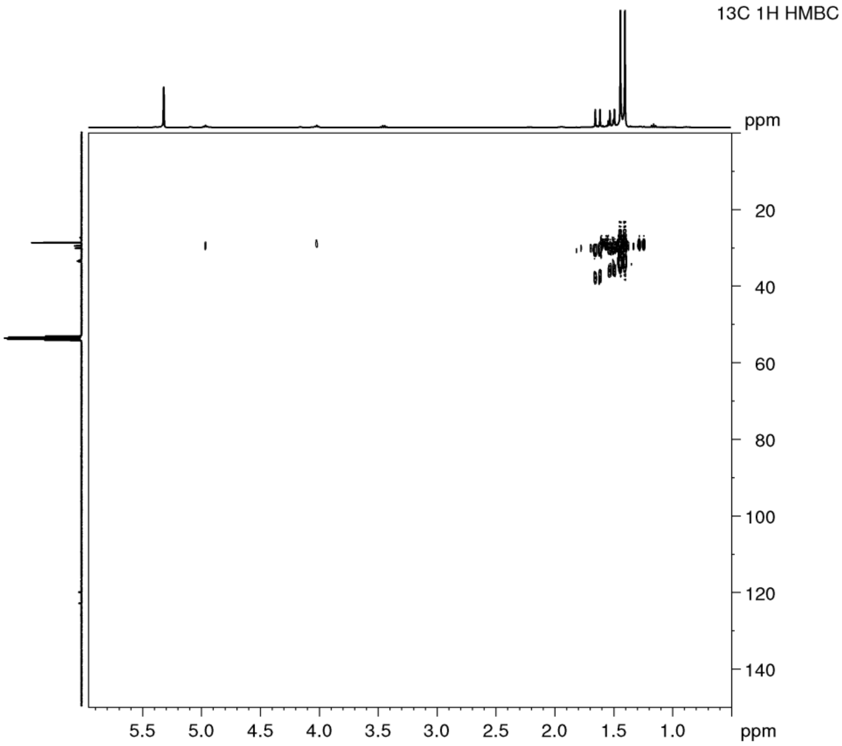


**Figure S36.** $^{13}C$ $^{1}H$ HMBC spectrum ($CD_2Cl_2$) of **5[WCA]**

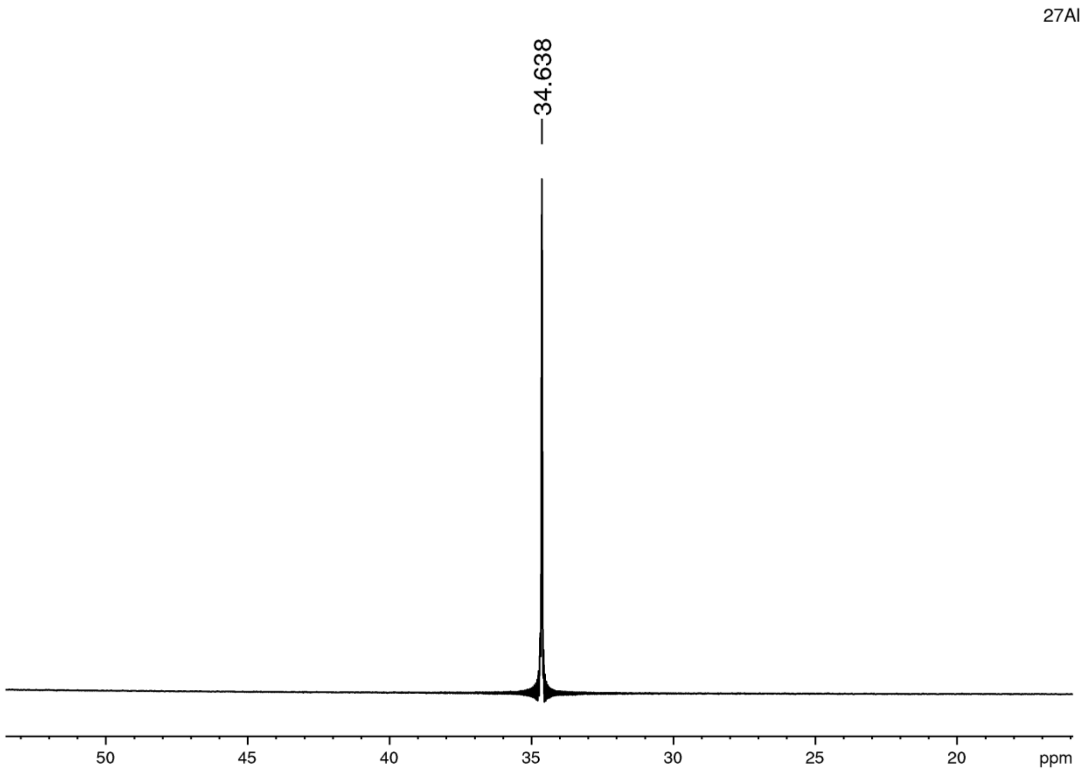


**Figure S37.** $^{27}Al$ spectrum ($CD_2Cl_2$) of **5[WCA]**

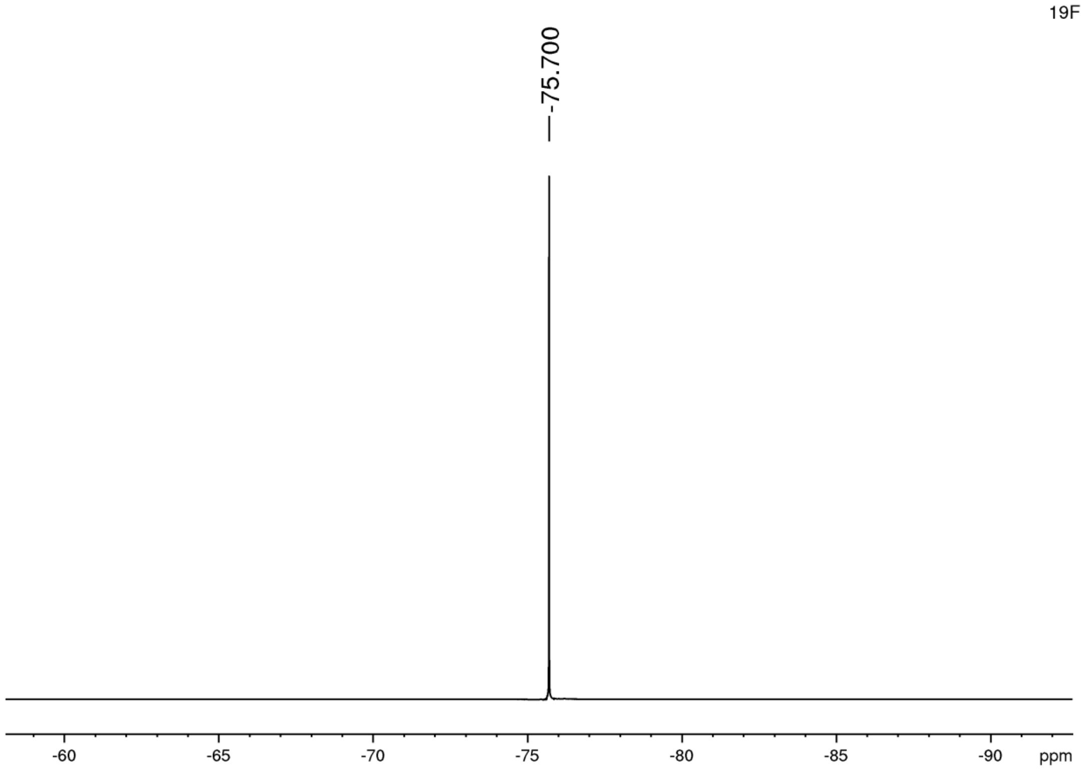


**Figure S38.** $^{19}F$ spectrum ($CD_2Cl_2$) of **5[WCA]**

# X-ray structure analysis

Crystal data, data collection and structure refinement details are summarized in Table S1.

Diffraction intensity data for all crystals were collected on an IPDS 2T dual beam diffractometer (STOE & Cie GmbH, Darmstadt, Germany) at 120.0(2) K with Mo *K*α radiation of a microfocus X-ray source (GeniX 3D Mo High Flux, Xenocs, Sassenage, 50 kV, 1.0 mA, and λ = 0.71069 Å) for most of the structures and Cu *K*α radiation of a microfocus X-ray source (GeniX 3D Mo High Flux, Xenocs, Sassenage, 50 kV, 0.6 mA, and λ = 1.54186 Å) in the case of **4[WCA]**. Investigated crystals were thermostated under a nitrogen stream at 120 K using the CryoStream-800 device (Oxford CryoSystem, UK) during the entire experiment. Data collection and data reduction were controlled by using the X-Area 1.75 program (STOE, 2015). Absorption correction was performed only for **4[WCA]** because of stronger absorption of copper radiation (μ> 0.5 $mm^{-1}$). Numerical absorption correction was performed by gaussian integration analogous to P. Coppens, "The Evaluation of Absorption and Extinction in Single-Crystal Structure Analysis", published in F. R. Ahmed (Editor), "Crystallographic Computing", Munksgaard, Copenhagen (1970), 255 – 270. The structures were solved using intrinsic phasing implemented in SHELXT and refined anisotropically using the program packages Olex2[6] and SHELX-2015.[7,8] Positions of hydrogen atoms were calculated geometrically taking into account isotropic temperature factors. All H-atoms were refined as riding on their parent atoms with the usual restraints.

## X-ray structure analysis details

All structures contain WCA anions, which are usually strongly disordered, that has a negative impact on refinement quality indicators like $R_1$ or $wR^2$. Nevertheless, the more interesting cations are essentially well localized and their geometry is determined reliably.

Structure of **2[WCA]** belongs to the monoclinic system, the space group $P2_1/n$, Z = 4, Z' = 1. Asymmetric unit contains one borinium cation ($C_{16}H_{26}BP_2^+$) and one aluminate anion ($WCA^-$ = $AlC_{16}F_{36}O_4^-$). Unexpectedly, the anion shows no disorder. One *tert*-butyl group in the cation, i.e. C13-C16, was refined as disordered over two positions with site occupation factors of 0.738(8)/0.262(8).

Structure of **3[WCA]** belongs to the triclinic system, the space group $P\bar{1}$, Z = 4, Z' = 2. Asymmetric unit contains two borenium cations ($C_{23}H_{48}BN_2P_2^+$), two aluminate anions ($WCA^-$ = $AlC_{16}F_{36}O_4^-$) and two dichloromethane (solvent) molecules: one ordered and one disordered (s.o.f. 0.511(6)/0.489(6)). The first anion (with O1-O4) is refined as strongly disordered at all alkoxy branches. The alkoxy residue at O1 is disordered by rotation of all three $CF_3$ groups at C47 atom by ca 60°. Further disorder (at O2, O3 and O4) starts from oxygen atoms and whole alkoxy groups are tilted in two positions. Occupation factors are respectively: 0.688(7)/0.312(7); 0.744(5)/0.253(5) and 0.692(5)/0.308(5). The other anion (O5-O8) has only one alkoxy group (with O6) disordered with C-$CF_3$ groups rotated ca 60° at C67 atom

in the second disorder part (s.o.f. 0.634(8)/366(8)). Fortunately, each of the two cations occupies only one, well defined, position in asymmetric unit.

Structure of **4[WCA]** belongs to the trigonal system, the space group $P3_1$, Z = 3, Z' = 1. Asymmetric unit contains one borenium cation ($C_{27}H_{56}BN_2P_2^+$) and one aluminate anion ($WCA^-$ = $AlC_{16}F_{36}O_4^-$). The anion is disordered at two alkoxy groups O3 and O4 in the tilt and rotate mode starting from the oxygen atoms. Site occupation factors were refined to 0.761(13)/0.239(13) and 0.808(18)/0.192(18) for O3 and O4 branches, repectively.

Structure of **5[WCA]** belongs to the triclinic system, the space group $P\bar{1}$, Z = 4, Z' = 2. Asymmetric unit contains two symmetry independent boronium cations ($BH_2(PH\ ^tBu_2)_2^+$) and two aluminate anions ($AlC_{16}F_{36}O_4^-$). One cation is fully ordered and the other one is modelled as disordered in two positions with occupation factors 0.677(2)/0.323(2). All P-H and B-H hydrogen atom positions were found in the Fourier electron density map and refined with mild restrains, to be of equal bond length within their types. Only this cation, being more reliable, was taken for analysis of geometry parameters, i.e. its bond lengths and angles. Precise location of P-H and B-H hydrogen atoms in the second, disordered, cation would be speculative and was left as undetermined in the CIF file. In summary: in the unit cell we have four cations and four anions. Since both independent anions are disordered over several conformations, only one cation is well positioned in the unit cell.

Crystallographic data for all structures reported in this paper have been deposited with the Cambridge Crystallographic Data Centre as supplementary publication Nos. **CCDC 2455327 – 2455330**. The data can be obtained free of charge from The Cambridge Crystallographic Data Centre via www.ccdc.cam.ac.uk/structures.

**Table S1.** Crystal data and structure refinement for **2[WCA]**, **3[WCA]**, **4[WCA]**, and **5[WCA]**

| | **2[WCA]** | **3[WCA]** | **4[WCA]** | **5[WCA]** |
|---|---|---|---|---|
| CCDC number | 2455327 | 2455328 | 2455329 | 2455330 |
| Empirical formula | $C_{16}AlF_{36}O_4^-$ $\cdot C_{16}H_{36}BP_2^+$ | $C_{16}AlF_{36}O_4^-$ $\cdot C_{23}H_{48}BN_2P_2^+$ $\cdot CH_2Cl_2$ | $C_{16}AlF_{36}O_4^-$ $\cdot C_{27}H_{56}BN_2P_2^+$ | $C_{16}AlF_{36}O_4^-$ $\cdot C_{16}H_{40}BP_2^+$ |
| Formula weight | 1268.34 | 1477.45 | 1448.62 | 1272.37 |
| Temperature/K | 120 | 120 | 120 | 120 |
| Crystal system | monoclinic | triclinic | trigonal | triclinic |
| Space group | $P2_1/n$ | $P$-1 | $P3_1$ | $P$-1 |
| a/Å | 11.6622(2) | 20.238(2) | 15.9121(13) | 16.578(2) |
| b/Å | 20.3909(4) | 20.360(2) | 15.9121 (13) | 16.838(2) |
| c/Å | 21.3006(3) | 20.160(2) | 20.735 (2) | 20.642(3) |
| α/° | 90 | 96.311(9) | 90 | 105.275(10) |
| β/° | 103.726(1) | 117.899(8) | 90 | 107.625(10) |
| γ/° | 90 | 117.537(8) | 120 | 103.339(10) |
| Volume/Å$^3$ | 4920.68(15) | 5918.8(13) | 4546.7(9) | 4986.1(12) |
| Z | 4 | 4 | 3 | 4 |
| $\rho_{calc}$g/cm$^3$ | 1.712 | 1.658 | 1.587 | 1.693 |
| μ/mm$^{-1}$ | 0.279 | 0.333 | 2.205 | 0.275 |
| F(000) | 2528 | 2968 | 2196 | 2538 |
| Crystal size/mm$^3$ | 0.03 (radius) | 0.33 x 0.09 x 0.06 | 0.16 x 0.03 x 0.02 | 0.18 x 0.09 x 0.06 |
| Radiation | MoKα (λ = 0.71073) | MoKα (λ = 0.71073) | CuKα (λ = 1.54186) | MoKα (λ = 0.71073) |
| 2Θ range for data collection/° | 1.833 to 29.390 | 2.50 to 22.60 | 3.207 to 65.997 | 1.555 to 26.000 |
| Index ranges | -16 ≤ h ≤ 16, -27 ≤ k ≤ 28, -29 ≤ l ≤ 29 | -27 ≤ h ≤ 27, -27 ≤ k ≤ 27, -26 ≤ l ≤ 27 | -16 ≤ h ≤ 18, -18 ≤ k ≤ 18, -23 ≤ l ≤ 24 | -20 ≤ h ≤ 20, -20 ≤ k ≤ 20, -25 ≤ l ≤ 25 |
| Reflections collected | 148945 | 88112 | 27531 | 50903 |
| Independent reflections | 13372 [$R_{int}$ = 0.0557 $R_{sigma}$ = 0.0224] | 31743 [$R_{int}$ = 0.0600 $R_{sigma}$ = 0.0579] | 10309 [$R_{int}$ = 0.0512 $R_{sigma}$ = 0.0650] | 19616 [$R_{int}$ = 0.0404 $R_{sigma}$ = 0.0434] |
| Data/restraints/parameters | 13372/713/3 | 31743/742/1918 | 10309/934/235 | 19616/340/1878 |
| Goodness-of-fit on F$^2$ | 1.029 | 1.047 | 1.068 | 1.022 |
| Final R indexes [I>=2σ (I)] | $R_1$ = 0.0581 $wR_2$ = 0.1502 | $R_1$ = 0.0957 $wR_2$ = 0.2069 | $R_1$ = 0.0834 $wR_2$ = 0.1751 | $R_1$ = 0.0996 $wR_2$ = 0.2616 |
| Final R indexes [all data] | $R_1$ = 0.0893 $wR_2$ = 0.1735 | $R_1$ = 0.1625 $wR_2$ = 0.2543 | $R_1$ = 0.1280 $wR_2$ = 0.2078 | $R_1$ = 0.1517 $wR_2$ = 0.3137 |
| Largest diff. peak/hole/e Å$^{-3}$ | 0.762/-0.651 | 1.449/-0.828 | 0.89/-0.38 | 1.548/-0.634 |

# X-ray structure analysis of 4[WCA]

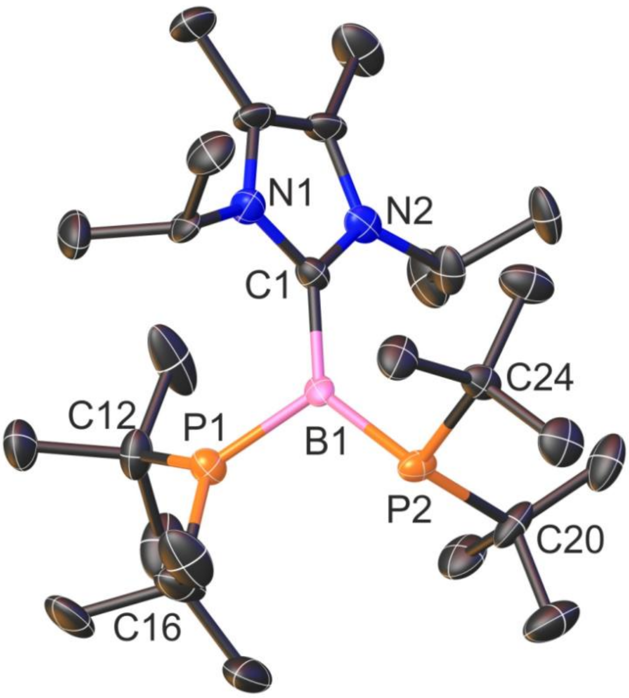


**Figure S39.** Molecular structure of NHC-stabilized borenium cation **4⁺**. The counterion and hydrogen atoms are omitted for clarity. The thermal ellipsoids are shown at the 50% probability level. Important bond distances [Å]: B1 – P1 1.82(1); B1 – P2 1.94(1); B1 – C1 1.61(2); C1 – N1 1.36(1); C1 – N2 1.36(1). Important angles [°]: P1 – B1 – P2 110.5(6); P2 – B1 – C1 132.0(9); C1 – B1 – P1 116.7(8).

# X-ray differential Fourier electron density map of 5[WCA]

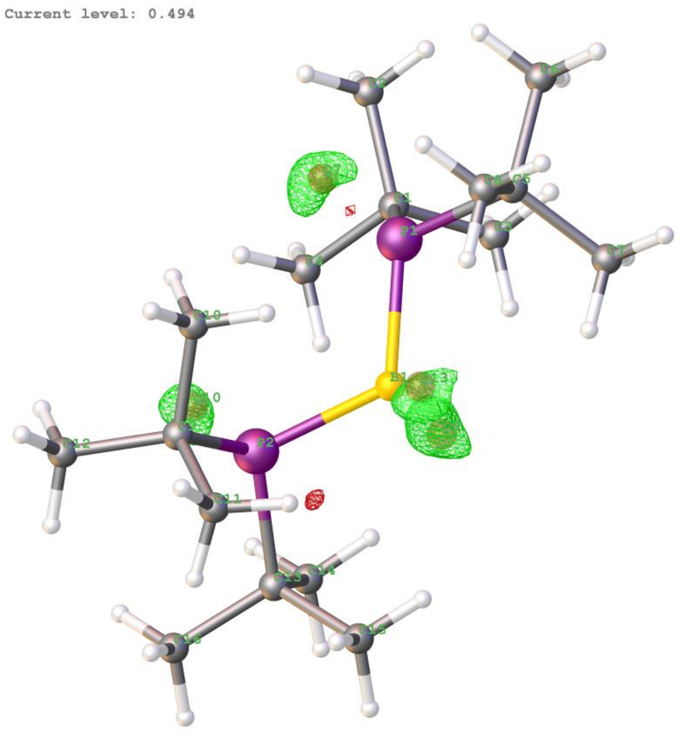


**Figure S 40.** The X-ray differential Fourier electron density map around boron and phosphorus atoms in boronium cation **5⁺**. Surfaces drawn at 0.494 e/ $Å^3$ density level.

# DFT calculations

The equilibrium geometries of all species were optimized using the long-range-corrected ωB97XD[9] functional, which includes empirical dispersion together with the 6-311++G(d,p)[10] basis set for all atoms. Vibrational frequency calculations were performed to verify that the optimized structures correspond to either true minima or transition states connecting reactants to products on the energy surface. The effects of surrounding solvent molecules (DFB) were approximated by employing the polarized continuum solvation model (PCM)[11] within a self-consistent reaction field treatment (the default options for PCM and the dielectric constant of 13.8 for DFB were used) for all structures investigated. The fluoride ion affinity (FIA)[12,13], hydride ion affinity (HIA)[14], and proton affinity (PA)[15,16] for $\mathbf{2}^+$ (as well as for $(iPr_2N)_2B^+$ and $(Mes)_2B^+$, which are used for comparison) are calculated (in accordance with the relevant definitions) as the negative of the enthalpy change in the corresponding reactions: $\mathbf{2}^+ + F^- \rightarrow \mathbf{2}$-F, $\mathbf{2}^+ + H^- \rightarrow \mathbf{2}$-H and $\mathbf{2}^+ + H^+ \rightarrow [\mathbf{2}\text{-H}]^{2+}$. These values were estimated in the gas phase at ωB97xD/6-311++G** level of theory (see ΔH values in Table 3S). Additionally, Table 3S provides the corresponding negative values of the electronic energy (ΔE) and Gibbs free energy (ΔG) changes for the reactions involving fluoride or hydride anion attachment and protonation. Natural bond orbital (NBO) analysis was performed with the Gaussian NBO 7 module.[17] All calculations were completed with Gaussian16 (Rev.C.01) software.[18]

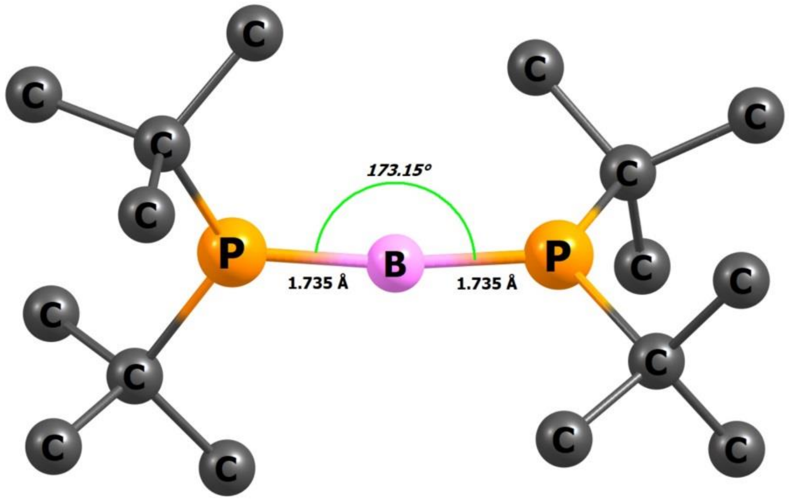


**Figure S41.** The ωB97XD/6-311++G(d,p) equilibrium geometry of the **2**+. The hydrogen atoms are omitted for clarity.

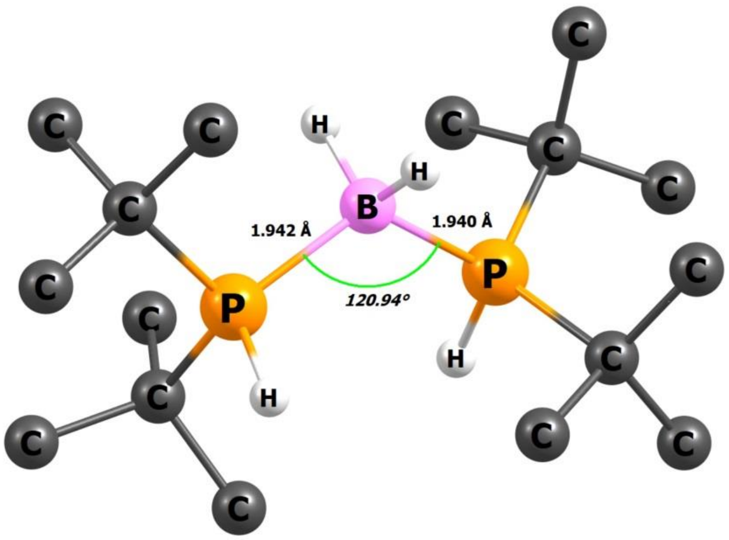


**Figure S42.** The ωB97XD/6-311++G(d,p) equilibrium geometry of the **5**⁺. The hydrogen atoms are omitted for clarity, except those bonded with boron and phosphorus.

**Table S2.** The cartesian coordinates (in Å) of all structures obtained at PCM-ωB97XD/6-311++(d,p) level of theory

| **TS 1** (*i*572 $cm^{-1}$) (Figure 3a) | | | |
|---|---|---|---|
| P | 1.647830000 | -0.229925000 | 0.022514000 |
| P | -1.850115000 | -0.507433000 | -0.696888000 |
| C | -2.756842000 | -1.306272000 | 0.763763000 |
| B | -0.018655000 | -0.637458000 | -0.415673000 |
| C | -2.295794000 | 1.293633000 | -1.062509000 |
| C | 1.882750000 | 1.059637000 | 1.365281000 |
| C | 3.970829000 | -1.805083000 | -0.037834000 |
| H | 3.375821000 | -2.680460000 | 0.230889000 |
| H | 4.839271000 | -2.146375000 | -0.609027000 |
| H | 4.337735000 | -1.338980000 | 0.876355000 |
| C | -1.558789000 | 1.635233000 | -2.367557000 |
| H | -1.848772000 | 0.967416000 | -3.182207000 |
| H | -1.811636000 | 2.659606000 | -2.658995000 |
| H | -0.472643000 | 1.582716000 | -2.246251000 |
| C | 3.169332000 | -0.833136000 | -0.916686000 |
| C | -4.108582000 | -1.821934000 | 0.243386000 |
| H | -3.974572000 | -2.530431000 | -0.577576000 |
| H | -4.630563000 | -2.336429000 | 1.056724000 |
| H | -4.753944000 | -1.013473000 | -0.104237000 |
| C | -2.984403000 | -0.371112000 | 1.953351000 |
| H | -3.638432000 | 0.464889000 | 1.697924000 |
| H | -3.475119000 | -0.934856000 | 2.754190000 |

| | | | |
|---|---|---|---|
| H | -2.048249000 | 0.024401000 | 2.350345000 |
| C | 4.011026000 | 0.401263000 | -1.279705000 |
| H | 4.384964000 | 0.932012000 | -0.404367000 |
| H | 4.881551000 | 0.057767000 | -1.845855000 |
| H | 3.453606000 | 1.100412000 | -1.906896000 |
| C | 2.764115000 | -1.543564000 | -2.216058000 |
| H | 2.088603000 | -0.942799000 | -2.829223000 |
| H | 3.677090000 | -1.712657000 | -2.793729000 |
| H | 2.318338000 | -2.521271000 | -2.033743000 |
| C | 3.114102000 | 0.699806000 | 2.210723000 |
| H | 4.048641000 | 0.775265000 | 1.654512000 |
| H | 3.169054000 | 1.415559000 | 3.036002000 |
| H | 3.035375000 | -0.301225000 | 2.640164000 |
| C | 2.042301000 | 2.463207000 | 0.763880000 |
| H | 1.189594000 | 2.742445000 | 0.143870000 |
| H | 2.117587000 | 3.184017000 | 1.583477000 |
| H | 2.944757000 | 2.550880000 | 0.158531000 |
| C | 0.643856000 | 0.995280000 | 2.269461000 |
| H | 0.546172000 | 0.016469000 | 2.743601000 |
| H | 0.754209000 | 1.747044000 | 3.056005000 |
| H | -0.275073000 | 1.217085000 | 1.729635000 |
| C | -1.927897000 | 2.327150000 | 0.003345000 |
| H | -2.350485000 | 2.097435000 | 0.981563000 |
| H | -0.846807000 | 2.421848000 | 0.103055000 |
| H | -2.313081000 | 3.305479000 | -0.303015000 |
| C | -3.811440000 | 1.334725000 | -1.313306000 |
| H | -4.075045000 | 2.321179000 | -1.707294000 |
| H | -4.126766000 | 0.584351000 | -2.042929000 |
| H | -4.381233000 | 1.187828000 | -0.393072000 |
| C | -1.909993000 | -2.509675000 | 1.204609000 |
| H | -2.468541000 | -3.078697000 | 1.954221000 |
| H | -1.698582000 | -3.186355000 | 0.371266000 |
| H | -0.964194000 | -2.200785000 | 1.657809000 |
| H | 0.401385000 | -2.307339000 | -0.884149000 |
| H | 0.250095000 | -1.991249000 | -1.568445000 |
| **IP** (*intermediate product*) (Figure 3a) | | | |
| P | 1.621053000 | 0.082400000 | -0.099880000 |

| | | | |
|---|---|---|---|
| P | -1.909726000 | 0.580365000 | -0.774534000 |
| C | -2.552430000 | -1.193385000 | -0.984983000 |
| B | 0.017412000 | 0.608522000 | -0.654151000 |
| C | -2.474969000 | 1.391253000 | 0.843016000 |
| C | 1.876579000 | -1.210214000 | 1.218834000 |
| C | 3.764370000 | -0.211272000 | -1.847088000 |
| H | 3.061857000 | -0.458113000 | -2.646261000 |
| H | 4.656434000 | 0.229174000 | -2.301677000 |
| H | 4.067633000 | -1.132378000 | -1.351187000 |
| C | -1.970101000 | 2.842654000 | 0.769308000 |
| H | -2.319381000 | 3.348625000 | -0.134368000 |
| H | -2.343365000 | 3.398384000 | 1.635709000 |
| H | -0.876991000 | 2.895294000 | 0.798964000 |
| C | 3.167977000 | 0.822720000 | -0.873449000 |
| C | -3.879997000 | -1.038729000 | -1.755027000 |
| H | -3.732595000 | -0.517933000 | -2.704154000 |
| H | -4.289470000 | -2.031800000 | -1.968910000 |
| H | -4.627037000 | -0.488686000 | -1.178047000 |
| C | -2.826071000 | -2.022684000 | 0.272525000 |
| H | -3.581313000 | -1.561050000 | 0.910971000 |
| H | -3.214359000 | -3.001060000 | -0.031161000 |
| H | -1.933862000 | -2.198205000 | 0.868553000 |
| C | 4.171906000 | 1.174089000 | 0.236265000 |
| H | 4.509374000 | 0.300539000 | 0.793077000 |
| H | 5.053301000 | 1.615134000 | -0.236520000 |
| H | 3.763502000 | 1.905720000 | 0.936648000 |
| C | 2.838186000 | 2.101442000 | -1.655336000 |
| H | 2.362313000 | 2.865385000 | -1.034824000 |
| H | 3.783054000 | 2.517763000 | -2.013102000 |
| H | 2.230140000 | 1.911890000 | -2.543394000 |
| C | 2.993791000 | -2.197168000 | 0.855107000 |
| H | 3.976713000 | -1.729146000 | 0.802957000 |
| H | 3.035368000 | -2.952701000 | 1.644561000 |
| H | 2.792184000 | -2.710597000 | -0.087258000 |
| C | 2.194005000 | -0.482161000 | 2.539568000 |
| H | 1.387758000 | 0.195043000 | 2.829571000 |
| H | 2.296195000 | -1.239536000 | 3.322045000 |

| | | | |
|---|---|---|---|
| H | 3.124971000 | 0.082307000 | 2.499062000 |
| C | 0.560801000 | -1.974900000 | 1.373706000 |
| H | 0.319431000 | -2.554886000 | 0.482120000 |
| H | 0.665558000 | -2.671644000 | 2.209610000 |
| H | -0.265936000 | -1.304762000 | 1.604190000 |
| C | -1.943336000 | 0.754840000 | 2.129186000 |
| H | -2.263335000 | -0.280881000 | 2.247194000 |
| H | -0.851150000 | 0.792793000 | 2.165341000 |
| H | -2.316716000 | 1.315884000 | 2.993183000 |
| C | -4.010239000 | 1.408852000 | 0.857814000 |
| H | -4.353986000 | 2.005820000 | 1.708956000 |
| H | -4.415574000 | 1.856758000 | -0.053624000 |
| H | -4.434705000 | 0.408896000 | 0.967569000 |
| C | -1.559266000 | -1.927881000 | -1.896681000 |
| H | -1.993445000 | -2.883901000 | -2.206546000 |
| H | -1.342060000 | -1.352628000 | -2.802150000 |
| H | -0.612798000 | -2.136664000 | -1.394739000 |
| H | 0.422007000 | 1.309020000 | -1.755844000 |
| H | 0.396078000 | 1.843328000 | -1.086134000 |
| **TS 2** (*i*1120 $cm^{-1}$) (Figure 3a) | | | |
| P | 1.647646000 | 0.030590000 | -0.142896000 |
| P | -1.906952000 | 0.503816000 | -0.716298000 |
| C | -2.607649000 | -1.230182000 | -0.967032000 |
| B | 0.056839000 | 0.541572000 | -0.696127000 |
| C | -2.440929000 | 1.439178000 | 0.829657000 |
| C | 1.896184000 | -1.142206000 | 1.293616000 |
| C | 3.863130000 | -0.293539000 | -1.815799000 |
| H | 3.194269000 | -0.658573000 | -2.598216000 |
| H | 4.733103000 | 0.163738000 | -2.296785000 |
| H | 4.215932000 | -1.145474000 | -1.235066000 |
| C | -1.869807000 | 2.857973000 | 0.675065000 |
| H | -2.225342000 | 3.340996000 | -0.238223000 |
| H | -2.192762000 | 3.462687000 | 1.527997000 |
| H | -0.775297000 | 2.857534000 | 0.669839000 |
| C | 3.175758000 | 0.771122000 | -0.944309000 |
| C | -3.939821000 | -1.038422000 | -1.714464000 |
| H | -3.791774000 | -0.527728000 | -2.668542000 |

| | | | |
|---|---|---|---|
| H | -4.376812000 | -2.021641000 | -1.915681000 |
| H | -4.662523000 | -0.467523000 | -1.127444000 |
| C | -2.861303000 | -2.018859000 | 0.319841000 |
| H | -3.591243000 | -1.528060000 | 0.966142000 |
| H | -3.272988000 | -2.996486000 | 0.048711000 |
| H | -1.950961000 | -2.192176000 | 0.890405000 |
| C | 4.132245000 | 1.279418000 | 0.145117000 |
| H | 4.510762000 | 0.475883000 | 0.777904000 |
| H | 4.995210000 | 1.739009000 | -0.345117000 |
| H | 3.660134000 | 2.034169000 | 0.778136000 |
| C | 2.763912000 | 1.950784000 | -1.834176000 |
| H | 2.242176000 | 2.731057000 | -1.274860000 |
| H | 3.675271000 | 2.391690000 | -2.247474000 |
| H | 2.151539000 | 1.635325000 | -2.682783000 |
| C | 3.048811000 | -2.115091000 | 1.011597000 |
| H | 4.015765000 | -1.614830000 | 0.947270000 |
| H | 3.103280000 | -2.821832000 | 1.844732000 |
| H | 2.883570000 | -2.686911000 | 0.096017000 |
| C | 2.173624000 | -0.333474000 | 2.571549000 |
| H | 1.345720000 | 0.337544000 | 2.809420000 |
| H | 2.291746000 | -1.034696000 | 3.403248000 |
| H | 3.087063000 | 0.257507000 | 2.501766000 |
| C | 0.599072000 | -1.936154000 | 1.464387000 |
| H | 0.385969000 | -2.557623000 | 0.592687000 |
| H | 0.706588000 | -2.594287000 | 2.331214000 |
| H | -0.248966000 | -1.278985000 | 1.653684000 |
| C | -1.925733000 | 0.829104000 | 2.134276000 |
| H | -2.279637000 | -0.190986000 | 2.288905000 |
| H | -0.833661000 | 0.834462000 | 2.165885000 |
| H | -2.281544000 | 1.434330000 | 2.974410000 |
| C | -3.974292000 | 1.506782000 | 0.835964000 |
| H | -4.294089000 | 2.152280000 | 1.659767000 |
| H | -4.364059000 | 1.927679000 | -0.094308000 |
| H | -4.430008000 | 0.526766000 | 0.992270000 |
| C | -1.632450000 | -1.990247000 | -1.875224000 |
| H | -2.089873000 | -2.941743000 | -2.162848000 |
| H | -1.415298000 | -1.435872000 | -2.793166000 |

| | | | |
|---|---|---|---|
| H | -0.688601000 | -2.206919000 | -1.371930000 |
| H | -0.616289000 | 1.259827000 | -1.672144000 |
| H | 0.384516000 | 1.398788000 | -1.607670000 |
| ***t*Bu$_2$P(H)–B(H)–P(*t*Bu)$_2$** (Figure 3a) | | | |
| P | -1.634493000 | -0.121056000 | 0.018755000 |
| P | 1.704418000 | 0.125697000 | -0.253478000 |
| C | 2.376051000 | 1.185331000 | 1.123594000 |
| B | 0.030141000 | -0.616618000 | 0.308429000 |
| C | 2.781330000 | -1.252294000 | -0.888765000 |
| C | -2.984850000 | -1.164664000 | 0.799255000 |
| C | -2.652919000 | 2.482980000 | -0.166115000 |
| H | -1.852985000 | 2.823244000 | 0.495519000 |
| H | -2.936867000 | 3.319424000 | -0.812612000 |
| H | -3.518028000 | 2.226623000 | 0.445226000 |
| C | 1.873676000 | -2.102597000 | -1.795207000 |
| H | 1.425991000 | -1.508219000 | -2.596692000 |
| H | 2.482103000 | -2.884266000 | -2.258460000 |
| H | 1.072766000 | -2.588410000 | -1.232728000 |
| C | -2.188460000 | 1.313464000 | -1.050370000 |
| C | 3.840137000 | 1.561363000 | 0.871977000 |
| H | 3.983917000 | 2.029498000 | -0.104924000 |
| H | 4.144608000 | 2.283067000 | 1.635134000 |
| H | 4.504622000 | 0.699419000 | 0.953170000 |
| C | 2.227891000 | 0.452428000 | 2.464550000 |
| H | 2.787760000 | -0.483648000 | 2.492795000 |
| H | 2.619266000 | 1.099503000 | 3.254290000 |
| H | 1.181637000 | 0.237495000 | 2.694825000 |
| C | -3.319807000 | 0.874242000 | -1.991544000 |
| H | -4.223180000 | 0.584784000 | -1.453571000 |
| H | -3.581505000 | 1.720592000 | -2.633771000 |
| H | -3.012681000 | 0.044418000 | -2.632247000 |
| C | -0.998511000 | 1.779762000 | -1.896840000 |
| H | -0.583650000 | 0.973520000 | -2.506797000 |
| H | -1.345215000 | 2.568197000 | -2.571091000 |
| H | -0.210954000 | 2.213843000 | -1.278766000 |
| C | -4.084704000 | -0.271806000 | 1.392223000 |
| H | -4.616162000 | 0.301395000 | 0.631316000 |

| | | | |
|---|---|---|---|
| H | -4.820633000 | -0.913297000 | 1.886294000 |
| H | -3.685512000 | 0.420203000 | 2.137554000 |
| C | -3.580606000 | -2.123628000 | -0.245291000 |
| H | -2.812597000 | -2.780328000 | -0.660274000 |
| H | -4.336336000 | -2.748048000 | 0.242220000 |
| H | -4.065722000 | -1.597757000 | -1.068151000 |
| C | -2.360938000 | -1.993001000 | 1.931597000 |
| H | -1.885248000 | -1.362516000 | 2.686122000 |
| H | -3.160946000 | -2.561271000 | 2.415435000 |
| H | -1.625891000 | -2.707692000 | 1.558277000 |
| C | 3.315473000 | -2.104332000 | 0.268022000 |
| H | 3.836328000 | -2.969347000 | -0.151525000 |
| H | 4.030913000 | -1.556091000 | 0.883955000 |
| H | 2.513090000 | -2.478231000 | 0.908636000 |
| C | 3.933234000 | -0.678227000 | -1.725675000 |
| H | 4.497858000 | -1.512931000 | -2.150500000 |
| H | 3.564555000 | -0.069323000 | -2.555209000 |
| H | 4.624918000 | -0.078652000 | -1.134379000 |
| C | 1.507308000 | 2.454742000 | 1.130993000 |
| H | 1.829199000 | 3.089017000 | 1.961251000 |
| H | 1.618828000 | 3.030513000 | 0.208561000 |
| H | 0.449187000 | 2.224500000 | 1.282163000 |
| H | 1.607436000 | 0.995130000 | -1.348093000 |
| H | 0.193711000 | -1.553451000 | 1.021316000 |
| **TS 1** (*i*432 $cm^{-1}$) (Figure 3b) | | | |
| P | -1.641532000 | 0.176849000 | 0.352036000 |
| P | 1.704973000 | 0.201199000 | -0.331936000 |
| C | 2.052444000 | 1.136718000 | 1.257418000 |
| B | -0.034334000 | -0.681617000 | -0.128242000 |
| C | 3.038473000 | -0.992144000 | -0.875769000 |
| C | -2.806998000 | -1.250180000 | 0.801222000 |
| C | -3.262606000 | 2.277626000 | -0.490157000 |
| H | -2.754384000 | 2.906590000 | 0.245270000 |
| H | -3.635057000 | 2.923550000 | -1.292279000 |
| H | -4.125729000 | 1.818274000 | -0.006468000 |
| C | 2.472949000 | -1.860433000 | -2.011634000 |
| H | 2.050902000 | -1.266059000 | -2.826207000 |

| | | | |
|---|---|---|---|
| H | 3.295854000 | -2.447575000 | -2.427407000 |
| H | 1.726513000 | -2.570975000 | -1.650384000 |
| C | -2.301984000 | 1.234053000 | -1.083573000 |
| C | 3.520412000 | 1.576921000 | 1.330353000 |
| H | 3.812345000 | 2.182122000 | 0.469198000 |
| H | 3.634673000 | 2.197444000 | 2.223365000 |
| H | 4.208996000 | 0.737785000 | 1.429031000 |
| C | 1.698158000 | 0.253103000 | 2.463800000 |
| H | 2.279866000 | -0.669424000 | 2.493302000 |
| H | 1.921628000 | 0.816267000 | 3.373874000 |
| H | 0.635855000 | 0.000276000 | 2.485100000 |
| C | -3.018870000 | 0.435040000 | -2.177729000 |
| H | -3.928492000 | -0.043638000 | -1.814056000 |
| H | -3.309088000 | 1.120889000 | -2.980888000 |
| H | -2.381948000 | -0.333559000 | -2.623840000 |
| C | -1.122397000 | 1.978483000 | -1.723346000 |
| H | -0.466731000 | 1.307116000 | -2.283745000 |
| H | -1.514155000 | 2.710208000 | -2.436592000 |
| H | -0.529427000 | 2.527834000 | -0.988943000 |
| C | -4.220402000 | -0.701989000 | 1.042864000 |
| H | -4.701701000 | -0.367324000 | 0.122361000 |
| H | -4.842123000 | -1.498342000 | 1.464700000 |
| H | -4.215403000 | 0.129431000 | 1.753219000 |
| C | -2.854275000 | -2.390350000 | -0.222729000 |
| H | -1.874185000 | -2.856094000 | -0.352004000 |
| H | -3.539516000 | -3.166723000 | 0.135145000 |
| H | -3.210099000 | -2.061891000 | -1.199621000 |
| C | -2.275639000 | -1.799629000 | 2.136737000 |
| H | -2.279003000 | -1.032217000 | 2.915127000 |
| H | -2.919762000 | -2.621818000 | 2.465669000 |
| H | -1.260016000 | -2.193913000 | 2.048639000 |
| C | 3.452897000 | -1.901048000 | 0.288297000 |
| H | 4.144140000 | -2.655964000 | -0.095794000 |
| H | 3.968582000 | -1.357730000 | 1.080688000 |
| H | 2.597476000 | -2.426500000 | 0.720690000 |
| C | 4.237163000 | -0.202818000 | -1.425067000 |
| H | 4.976853000 | -0.920251000 | -1.790579000 |

| | | | |
|---|---|---|---|
| H | 3.946545000 | 0.432514000 | -2.265516000 |
| H | 4.723686000 | 0.415278000 | -0.673130000 |
| C | 1.168144000 | 2.393801000 | 1.253330000 |
| H | 1.362473000 | 2.941196000 | 2.179790000 |
| H | 1.408426000 | 3.057018000 | 0.418386000 |
| H | 0.104173000 | 2.151931000 | 1.232347000 |
| H | 1.692277000 | 1.182958000 | -1.334160000 |
| H | 0.138120000 | -1.759453000 | 0.352406000 |
| H | -0.500522000 | -1.027718000 | -1.823530000 |
| H | -0.086800000 | -1.667080000 | -1.787535000 |
| **IP 1** (Figure 3b) | | | |
| P | 1.643906000 | -0.180028000 | 0.419491000 |
| P | -1.710839000 | -0.285478000 | -0.270302000 |
| C | -2.044042000 | -0.833361000 | 1.483280000 |
| B | 0.021675000 | 0.625271000 | -0.346535000 |
| C | -3.029958000 | 0.756707000 | -1.084261000 |
| C | 2.752819000 | 1.350298000 | 0.617046000 |
| C | 3.369415000 | -2.260677000 | -0.225713000 |
| H | 2.923056000 | -2.799112000 | 0.614464000 |
| H | 3.738705000 | -2.999394000 | -0.945068000 |
| H | 4.229746000 | -1.703502000 | 0.147107000 |
| C | -2.445650000 | 1.261772000 | -2.415847000 |
| H | -2.027447000 | 0.455126000 | -3.025775000 |
| H | -3.252196000 | 1.722220000 | -2.991712000 |
| H | -1.689130000 | 2.037213000 | -2.267498000 |
| C | 2.334443000 | -1.352696000 | -0.912078000 |
| C | -3.520987000 | -1.194436000 | 1.683214000 |
| H | -3.853950000 | -1.959216000 | 0.977503000 |
| H | -3.630524000 | -1.603910000 | 2.691079000 |
| H | -4.179384000 | -0.328074000 | 1.611234000 |
| C | -1.628091000 | 0.280150000 | 2.456132000 |
| H | -2.177093000 | 1.208186000 | 2.285973000 |
| H | -1.847654000 | -0.054937000 | 3.473439000 |
| H | -0.557689000 | 0.489266000 | 2.400783000 |
| C | 2.969254000 | -0.692849000 | -2.140596000 |
| H | 3.871897000 | -0.134250000 | -1.889817000 |
| H | 3.253580000 | -1.470100000 | -2.858287000 |

| | | | |
|---|---|---|---|
| H | 2.286457000 | -0.014514000 | -2.663380000 |
| C | 1.172038000 | -2.242712000 | -1.382727000 |
| H | 0.469851000 | -1.704814000 | -2.026583000 |
| H | 1.572927000 | -3.069279000 | -1.977514000 |
| H | 0.620479000 | -2.679958000 | -0.545674000 |
| C | 4.188431000 | 0.893879000 | 0.913665000 |
| H | 4.677935000 | 0.467500000 | 0.036125000 |
| H | 4.780258000 | 1.759582000 | 1.228760000 |
| H | 4.220476000 | 0.156554000 | 1.720922000 |
| C | 2.758858000 | 2.337240000 | -0.556887000 |
| H | 1.770514000 | 2.770030000 | -0.731010000 |
| H | 3.433648000 | 3.168428000 | -0.324122000 |
| H | 3.107280000 | 1.884866000 | -1.485529000 |
| C | 2.215785000 | 2.070358000 | 1.866790000 |
| H | 2.258953000 | 1.426147000 | 2.748839000 |
| H | 2.828523000 | 2.956540000 | 2.062959000 |
| H | 1.183882000 | 2.408646000 | 1.739323000 |
| C | -3.393743000 | 1.954824000 | -0.198591000 |
| H | -4.080598000 | 2.599113000 | -0.754152000 |
| H | -3.897713000 | 1.653254000 | 0.720876000 |
| H | -2.517350000 | 2.553527000 | 0.062425000 |
| C | -4.266386000 | -0.101321000 | -1.393119000 |
| H | -4.981639000 | 0.516444000 | -1.942977000 |
| H | -4.014068000 | -0.958206000 | -2.022778000 |
| H | -4.766766000 | -0.462969000 | -0.497192000 |
| C | -1.195249000 | -2.093421000 | 1.727760000 |
| H | -1.378249000 | -2.428515000 | 2.752660000 |
| H | -1.477489000 | -2.907478000 | 1.055119000 |
| H | -0.126803000 | -1.896337000 | 1.628644000 |
| H | -1.719390000 | -1.470192000 | -1.023225000 |
| H | -0.120824000 | 1.732349000 | 0.076563000 |
| H | 0.563023000 | 0.565726000 | -1.578260000 |
| H | -0.021228000 | 1.167750000 | -1.617817000 |
| **TS 2** (*i*30 $cm^{-1}$) (Figure 3b) | | | |
| P | 1.985756000 | 0.413819000 | -0.826836000 |
| P | -1.598483000 | 0.076678000 | 0.180606000 |
| C | -2.930178000 | 1.222017000 | -0.473595000 |

| | | | |
|---|---|---|---|
| B | 0.012605000 | 0.318246000 | -0.963671000 |
| C | -2.140461000 | -1.658612000 | 0.605716000 |
| C | 2.764482000 | -1.282642000 | -0.483565000 |
| C | 3.795568000 | 1.711957000 | 0.854058000 |
| H | 4.347345000 | 1.888414000 | -0.073286000 |
| H | 3.993569000 | 2.551870000 | 1.528019000 |
| H | 4.193171000 | 0.813360000 | 1.329672000 |
| C | -0.883438000 | -2.392702000 | 1.087760000 |
| H | -0.448084000 | -1.905901000 | 1.964700000 |
| H | -1.163368000 | -3.408973000 | 1.376856000 |
| H | -0.121376000 | -2.470041000 | 0.315255000 |
| C | 2.281047000 | 1.614478000 | 0.610882000 |
| C | -4.259677000 | 1.020553000 | 0.263862000 |
| H | -4.148230000 | 1.083843000 | 1.348321000 |
| H | -4.941139000 | 1.817229000 | -0.046583000 |
| H | -4.731630000 | 0.071886000 | 0.004975000 |
| C | -3.138457000 | 0.996727000 | -1.978409000 |
| H | -3.412625000 | -0.033356000 | -2.213892000 |
| H | -3.957856000 | 1.639285000 | -2.311227000 |
| H | -2.260203000 | 1.271317000 | -2.565772000 |
| C | 1.580674000 | 1.290882000 | 1.936307000 |
| H | 1.758713000 | 0.270728000 | 2.274747000 |
| H | 1.947867000 | 1.970215000 | 2.713763000 |
| H | 0.504053000 | 1.449891000 | 1.865042000 |
| C | 1.789204000 | 2.986586000 | 0.119400000 |
| H | 0.710265000 | 2.998542000 | -0.055661000 |
| H | 2.001036000 | 3.740207000 | 0.885126000 |
| H | 2.291035000 | 3.289230000 | -0.803157000 |
| C | 4.242039000 | -1.177443000 | -0.913491000 |
| H | 4.811129000 | -0.487077000 | -0.290517000 |
| H | 4.710162000 | -2.163841000 | -0.825395000 |
| H | 4.332038000 | -0.851297000 | -1.952810000 |
| C | 2.707531000 | -1.813882000 | 0.950743000 |
| H | 1.694345000 | -1.844057000 | 1.347380000 |
| H | 3.108583000 | -2.833345000 | 0.977792000 |
| H | 3.316230000 | -1.208572000 | 1.625410000 |
| C | 2.099745000 | -2.275199000 | -1.451228000 |

| | | | |
|---|---|---|---|
| H | 2.115110000 | -1.905094000 | -2.481677000 |
| H | 2.650079000 | -3.220983000 | -1.436022000 |
| H | 1.067035000 | -2.511412000 | -1.185261000 |
| C | -2.739241000 | -2.353650000 | -0.622153000 |
| H | -2.957926000 | -3.392480000 | -0.360662000 |
| H | -3.676806000 | -1.889909000 | -0.935073000 |
| H | -2.056034000 | -2.368640000 | -1.475360000 |
| C | -3.146133000 | -1.648977000 | 1.768931000 |
| H | -3.281903000 | -2.682684000 | 2.098637000 |
| H | -2.774924000 | -1.076224000 | 2.622214000 |
| H | -4.123626000 | -1.266141000 | 1.487085000 |
| C | -2.436170000 | 2.656707000 | -0.218653000 |
| H | -3.190489000 | 3.350113000 | -0.600113000 |
| H | -2.304827000 | 2.857195000 | 0.847451000 |
| H | -1.501484000 | 2.881058000 | -0.735446000 |
| H | -1.288297000 | 0.618215000 | 1.435597000 |
| H | -0.212151000 | 1.367939000 | -1.505687000 |
| H | -0.014607000 | -0.710702000 | -1.823976000 |
| H | -0.375500000 | -0.029298000 | -2.198067000 |
| **IP 2** (Figure 3b) | | | |
| P | -1.936845000 | 0.531373000 | -0.781815000 |
| P | 1.553010000 | 0.067954000 | 0.126313000 |
| C | 2.100788000 | -1.597716000 | 0.774843000 |
| B | -0.072999000 | -0.118736000 | -0.983088000 |
| C | 2.871216000 | 1.122834000 | -0.689852000 |
| C | -2.052978000 | 1.537442000 | 0.820960000 |
| C | -4.340189000 | -0.818345000 | -0.206324000 |
| H | -4.789476000 | 0.005316000 | -0.767649000 |
| H | -4.950095000 | -1.711508000 | -0.377569000 |
| H | -4.395416000 | -0.587784000 | 0.859394000 |
| C | 2.207192000 | 2.369708000 | -1.301535000 |
| H | 1.615208000 | 2.930556000 | -0.576246000 |
| H | 3.004689000 | 3.030093000 | -1.651170000 |
| H | 1.587218000 | 2.152705000 | -2.173105000 |
| C | -2.900717000 | -1.106723000 | -0.655840000 |
| C | 3.476598000 | -1.521508000 | 1.448829000 |
| H | 3.520229000 | -0.750433000 | 2.220929000 |

| | | | |
|---|---|---|---|
| H | 3.661841000 | -2.483880000 | 1.933797000 |
| H | 4.282556000 | -1.358443000 | 0.732109000 |
| C | 2.128863000 | -2.645586000 | -0.345545000 |
| H | 2.801603000 | -2.371138000 | -1.160092000 |
| H | 2.490955000 | -3.585189000 | 0.079982000 |
| H | 1.138651000 | -2.835255000 | -0.761580000 |
| C | -2.309202000 | -2.190089000 | 0.249648000 |
| H | -2.200435000 | -1.855940000 | 1.282492000 |
| H | -2.974441000 | -3.060706000 | 0.253840000 |
| H | -1.338949000 | -2.535977000 | -0.114019000 |
| C | -2.951958000 | -1.644265000 | -2.097008000 |
| H | -1.956025000 | -1.846967000 | -2.501239000 |
| H | -3.507731000 | -2.587895000 | -2.110269000 |
| H | -3.455247000 | -0.942647000 | -2.767133000 |
| C | -3.438010000 | 2.209798000 | 0.866359000 |
| H | -4.250774000 | 1.497256000 | 0.997334000 |
| H | -3.467685000 | 2.901265000 | 1.715282000 |
| H | -3.629531000 | 2.784931000 | -0.043382000 |
| C | -1.824844000 | 0.758364000 | 2.119100000 |
| H | -0.874847000 | 0.219368000 | 2.122837000 |
| H | -1.816246000 | 1.450823000 | 2.968276000 |
| H | -2.621810000 | 0.033790000 | 2.297640000 |
| C | -1.023782000 | 2.672050000 | 0.698311000 |
| H | -1.109125000 | 3.195209000 | -0.258810000 |
| H | -1.195498000 | 3.402497000 | 1.495045000 |
| H | 0.002137000 | 2.323607000 | 0.808275000 |
| C | 3.579256000 | 0.317504000 | -1.786438000 |
| H | 4.276425000 | 0.979406000 | -2.306881000 |
| H | 4.156045000 | -0.514043000 | -1.378791000 |
| H | 2.884223000 | -0.076618000 | -2.532958000 |
| C | 3.873656000 | 1.602573000 | 0.373075000 |
| H | 4.585746000 | 2.274065000 | -0.114013000 |
| H | 3.378761000 | 2.163232000 | 1.170043000 |
| H | 4.442927000 | 0.790063000 | 0.818484000 |
| C | 1.053592000 | -1.982632000 | 1.833626000 |
| H | 1.273952000 | -2.994339000 | 2.184457000 |
| H | 1.088049000 | -1.313105000 | 2.696766000 |

| | | | |
|---|---|---|---|
| H | 0.039077000 | -1.988885000 | 1.435008000 |
| H | 1.256602000 | 0.774871000 | 1.299837000 |
| H | -0.106731000 | -1.238777000 | -1.405235000 |
| H | 0.174804000 | 0.791663000 | -1.978080000 |
| H | 0.403981000 | 0.042136000 | -2.258892000 |
| **TS 3** (*i*768 $cm^{-1}$) (Figure 3b) | | | |
| P | -1.977658000 | 0.530750000 | -0.747376000 |
| P | 1.559970000 | 0.065410000 | 0.121654000 |
| C | 2.143755000 | -1.580009000 | 0.788525000 |
| B | -0.010549000 | -0.220965000 | -0.995619000 |
| C | 2.846478000 | 1.122141000 | -0.731799000 |
| C | -2.017537000 | 1.534740000 | 0.852911000 |
| C | -4.362967000 | -0.793878000 | -0.233368000 |
| H | -4.789301000 | 0.044386000 | -0.790438000 |
| H | -4.985958000 | -1.673208000 | -0.423283000 |
| H | -4.424138000 | -0.577516000 | 0.834869000 |
| C | 2.138180000 | 2.331999000 | -1.365389000 |
| H | 1.607896000 | 2.937052000 | -0.627704000 |
| H | 2.904871000 | 2.967081000 | -1.816921000 |
| H | 1.444648000 | 2.055136000 | -2.160514000 |
| C | -2.923962000 | -1.114477000 | -0.669533000 |
| C | 3.508679000 | -1.454584000 | 1.476745000 |
| H | 3.523492000 | -0.667004000 | 2.233805000 |
| H | 3.717628000 | -2.401946000 | 1.981384000 |
| H | 4.315931000 | -1.280857000 | 0.763829000 |
| C | 2.214706000 | -2.638561000 | -0.320562000 |
| H | 2.894242000 | -2.355970000 | -1.126154000 |
| H | 2.590802000 | -3.565452000 | 0.120934000 |
| H | 1.235929000 | -2.850965000 | -0.751591000 |
| C | -2.358890000 | -2.199718000 | 0.247989000 |
| H | -2.257851000 | -1.861942000 | 1.280151000 |
| H | -3.045366000 | -3.052744000 | 0.245534000 |
| H | -1.392692000 | -2.567005000 | -0.100440000 |
| C | -2.958063000 | -1.632743000 | -2.116611000 |
| H | -1.959185000 | -1.850442000 | -2.502828000 |
| H | -3.531357000 | -2.564815000 | -2.143667000 |
| H | -3.439376000 | -0.916889000 | -2.787432000 |

| | | | |
|---|---|---|---|
| C | -3.404517000 | 2.202076000 | 0.934040000 |
| H | -4.212352000 | 1.486197000 | 1.076836000 |
| H | -3.411267000 | 2.880183000 | 1.793356000 |
| H | -3.617429000 | 2.790076000 | 0.037956000 |
| C | -1.758714000 | 0.736455000 | 2.131785000 |
| H | -0.821563000 | 0.178739000 | 2.096138000 |
| H | -1.707418000 | 1.422499000 | 2.983713000 |
| H | -2.565686000 | 0.028628000 | 2.331299000 |
| C | -0.992423000 | 2.667374000 | 0.698534000 |
| H | -1.110471000 | 3.196425000 | -0.251230000 |
| H | -1.142593000 | 3.391363000 | 1.504735000 |
| H | 0.035557000 | 2.319762000 | 0.774477000 |
| C | 3.548908000 | 0.304948000 | -1.823359000 |
| H | 4.218627000 | 0.968687000 | -2.376659000 |
| H | 4.154879000 | -0.501392000 | -1.406850000 |
| H | 2.842041000 | -0.122742000 | -2.539667000 |
| C | 3.858817000 | 1.650061000 | 0.296870000 |
| H | 4.537936000 | 2.336280000 | -0.216406000 |
| H | 3.366575000 | 2.207660000 | 1.097795000 |
| H | 4.463580000 | 0.862246000 | 0.741088000 |
| C | 1.093192000 | -1.987935000 | 1.835218000 |
| H | 1.339935000 | -2.988745000 | 2.199615000 |
| H | 1.093047000 | -1.310546000 | 2.693122000 |
| H | 0.085668000 | -2.029727000 | 1.419243000 |
| H | 1.279191000 | 0.781029000 | 1.294629000 |
| H | -0.195158000 | -1.352331000 | -1.289993000 |
| H | -0.559193000 | 0.691316000 | -1.734807000 |
| H | 0.380088000 | 0.222575000 | -2.105963000 |
| ***t*Bu$_2$P(H)–B(H$_2$)–(H)P*t*Bu$_2$** (Figure 3b) | | | |
| P | -1.706429000 | 0.292399000 | -0.278679000 |
| P | 1.630868000 | 0.048792000 | 0.186967000 |
| C | 2.752385000 | -1.388565000 | 0.585341000 |
| B | -0.027261000 | -0.648143000 | -0.541107000 |
| C | 2.367324000 | 1.391250000 | -0.878476000 |
| C | -2.049248000 | 0.793426000 | 1.488089000 |
| C | -4.300149000 | 0.138972000 | -1.351770000 |
| H | -4.064806000 | 1.026322000 | -1.945378000 |

| | | | |
|---|---|---|---|
| H | -5.017429000 | -0.460105000 | -1.920376000 |
| H | -4.793616000 | 0.455488000 | -0.434968000 |
| C | 1.204794000 | 2.295352000 | -1.321818000 |
| H | 0.663054000 | 2.721160000 | -0.472726000 |
| H | 1.615979000 | 3.129653000 | -1.896788000 |
| H | 0.501191000 | 1.763628000 | -1.965375000 |
| C | -3.051139000 | -0.718701000 | -1.098070000 |
| C | 4.166058000 | -0.922219000 | 0.952057000 |
| H | 4.159381000 | -0.188466000 | 1.762058000 |
| H | 4.736487000 | -1.790035000 | 1.295585000 |
| H | 4.697876000 | -0.500257000 | 0.098000000 |
| C | 2.801518000 | -2.370623000 | -0.593511000 |
| H | 3.246638000 | -1.926461000 | -1.485138000 |
| H | 3.419168000 | -3.225861000 | -0.304575000 |
| H | 1.809022000 | -2.744577000 | -0.851404000 |
| C | -3.392171000 | -1.945366000 | -0.241831000 |
| H | -3.868875000 | -1.675270000 | 0.701986000 |
| H | -4.095641000 | -2.572101000 | -0.797379000 |
| H | -2.506948000 | -2.548796000 | -0.026729000 |
| C | -2.502554000 | -1.186476000 | -2.457997000 |
| H | -1.686371000 | -1.900399000 | -2.345945000 |
| H | -3.314340000 | -1.680294000 | -2.999477000 |
| H | -2.152012000 | -0.350970000 | -3.070619000 |
| C | -3.526184000 | 1.121716000 | 1.738353000 |
| H | -4.168350000 | 0.243863000 | 1.661012000 |
| H | -3.621045000 | 1.506378000 | 2.757959000 |
| H | -3.894449000 | 1.892698000 | 1.058025000 |
| C | -1.594955000 | -0.330181000 | 2.430828000 |
| H | -0.527861000 | -0.540836000 | 2.338566000 |
| H | -1.784787000 | -0.020965000 | 3.462403000 |
| H | -2.139303000 | -1.260028000 | 2.254579000 |
| C | -1.230251000 | 2.069710000 | 1.748799000 |
| H | -1.559922000 | 2.895990000 | 1.114083000 |
| H | -1.373940000 | 2.368113000 | 2.790949000 |
| H | -0.159627000 | 1.927875000 | 1.600034000 |
| C | 3.031551000 | 0.783830000 | -2.120648000 |
| H | 3.331663000 | 1.597682000 | -2.786813000 |

| | | | |
|---|---|---|---|
| H | 3.929007000 | 0.215184000 | -1.871736000 |
| H | 2.347084000 | 0.135567000 | -2.673787000 |
| C | 3.371787000 | 2.230328000 | -0.076173000 |
| H | 3.727467000 | 3.047112000 | -0.710989000 |
| H | 2.908417000 | 2.673435000 | 0.809319000 |
| H | 4.241748000 | 1.656191000 | 0.240533000 |
| C | 2.121376000 | -2.078410000 | 1.808411000 |
| H | 2.726325000 | -2.953695000 | 2.061218000 |
| H | 2.106977000 | -1.419930000 | 2.681361000 |
| H | 1.104805000 | -2.423991000 | 1.609939000 |
| H | 1.512742000 | 0.671892000 | 1.442133000 |
| H | -0.208553000 | -1.738227000 | -0.066025000 |
| H | -1.766375000 | 1.512800000 | -0.975097000 |
| H | 0.168088000 | -0.701173000 | -1.726714000 |

**Table S3**. The values calculated at ωB97XD/6-311++(d,p) level of theory for fluoride ion affinities (FIA in kJ/mol; ΔE/ΔH/ΔG ($F^-$)), hydride ion affinities (HIA in kJ/mol; ΔE/ΔH/ΔG ($H^-$)) and proton affinities (PA in kJ/mol; ΔE/ΔH/ΔG ($H^+$) as well as HOMO and LUMO energies (in eV) and HOMO-LUMO GAPs (in eV).

| molecule | **ΔE/ΔH/ΔG** ($F^-$) | **ΔE/ΔH/ΔG** ($H^-$) | **ΔE/ΔH/ΔG** ($H^+$) | HOMO | LUMO | HOMO-LUMO **GAP** |
|---|---|---|---|---|---|---|
| [$2^+$] | **792.8/790.2/729.3** | **831.7/816.1/761.4** | **601.2/586.1/545.4** | -11.89 | -3.32 | **8.57** |
| $(iPr_2N)_2B^+$ | **766.3/762.0/715.1** | **790.1/771.1/729.2** | **462.4/439.0/397.8** | -13.66 | -2.12 | **11.55** |
| $(Mes)_2B^+$ | **807.8/801.5/755.2** | **817.8/804.2/750.5** | **544.9/526.4/501.3** | -12.32 | -4.39 | **7.93** |

# References


1 A. M. Borys, *Organometallics*, 2023, **42**, 182–196.

2 G. R. Fulmer, A. J. M. Miller, N. H. Sherden, H. E. Gottlieb, A. Nudelman, B. M. Stoltz, J. E. Bercaw and K. I. Goldberg, *Organometallics*, 2010, **29**, 2176–2179.

3 A. Ordyszewska, N. Szynkiewicz, J. Chojnacki and R. Grubba, *Inorg. Chem.*, 2022, **61**, 4361–4370.

4 N. Kuhn and T. Kratz, *Synthesis (Stuttg).*, 1993, **6**, 561–562.

5 I. Krossing, *Chem. - A Eur. J.*, 2001, **7**, 490–502.

6 O. V Dolomanov, L. J. Bourhis, R. J. Gildea, J. A. K. Howard and H. Puschmann, *J. Appl. Crystallogr.*, 2009, **42**, 339–341.

7 G. M. Sheldrick, *Acta Cryst. A*, 2015, **71**, 3–8.

8 G. M. Sheldrick, *Acta Cryst. C*, 2015, **71**, 3–8.

9 J. Da Chai and M. Head-Gordon, *Phys. Chem. Chem. Phys.*, 2008, **10**, 6615–6620.

10 R. Krishnan, J. S. Binkley, R. Seeger and J. A. Pople, *J. Chem. Physicsournal Chem. Phys.*, 1980, **72**, 650–654.

11 S. Miertuš, E. Scrocco and J. Tomasi, *Chem. Phys.*, 1981, **55**, 117–129.

12 P. Erdmann, J. Leitner, J. Schwarz and L. Greb, *ChemPhysChem.*, 2020, **21**, 987–994.

13 H. Böhrer, N. Trapp, D. Himmel, M. Schlepp and I. Krossing, *Dalton Trans.*, 2015, **44**, 7489–7499.

14 D. J. Goebbert and P. G. Wenthold, *Int. J. Mass Spectrom.*, 2006, **257**, 1–11.

15 *'proton affinity' in IUPAC Compendium of Chemical Terminology, 5th ed. International Union of Pure and Applied Chemistry; 2025. Online version 5.0.0, 2025. doi:10.1351/goldbook.P04907*

16 E. P. L. Hunter and S. G. Lias, *J. Phys. Chem. Ref. Data*, 1998, **27**, 413–656.

17 E. D. Glendening, J. K. Badenhoop, A. E. Reed, J. E. Carpenter, J. A. Bohmann, C. M. Morales, P. Karafiloglou, C. R. Landis and F. Weinhold, *NBO 7.0.*, Theoretical Chemistry Institute, University of Wisconsin, Madison, 2018.

18 M. J. Frisch, G. W. Trucks, H. B. Schlegel, G. E. Scuseria, M. A. Robb, J. R. Cheeseman, G. Scalmani, V. Barone, B. Mennucci, G. A. Petersson, H. Nakatsuji, M. Caricato, X. Li, H. P. Hratchian, A. F. Izmaylov, J. Bloino, G. Zheng, J. L. Sonnenberg, M. Hada, M. Ehara, K. Toyota, R. Fukuda, J. Hasegawa, M. Ishida, T. Nakajima, Y. Honda, O. Kitao, H. Nakai, T. Vreven, J. A. Montgomery, Jr., J. E. Peralta, F. Ogliaro, M. Bearpark, J. J. Heyd, E. Brothers, K. N. Kudin, V. N. Staroverov, R. Kobayashi, J. Normand, K. Raghavachari, A. Rendell, J. C. Burant, S. S. Iyengar, J. Tomasi, M. Cossi, N. Rega, J. M. Millam, M. Klene, J. E. Knox, J. B. Cross, V. Bakken, C. Adamo, J. Jaramillo, R. Gomperts, R. E. Stratmann, O. Yazyev, A. J. Austin, R. Cammi, C. Pomelli, J. W. Ochterski, R. L. Martin, K. Morokuma, V. G. Zakrzewski, G. A. Voth, P. Salvador, J. J. Dannenberg, S. Dapprich, A. D. Daniels, Ö. Farkas, J. B. Foresman, J. V. Ortiz, J. Cioslowski and D. J. Fox, *Gaussian 16*, Gaussian, Inc., Wallingford CT, 2016.
